# Comparison of the diagnostic accuracy of shear wave elastography with transient elastography in adult nonalcoholic fatty liver disease: a systematic review and network meta-analysis of diagnostic test accuracy

**DOI:** 10.1007/s00261-024-04546-8

**Published:** 2024-09-06

**Authors:** Ruri Yamaguchi, Tetsuro Oda, Kengo Nagashima

**Affiliations:** 1 Department of Investigative Pathology, Tohoku University Graduate School of Medicine, 2-1 Seiryomachi, Aobaku, Sendai 980-8575 Japan; 2 Chugai Pharmaceutical Co., Ltd., Tokyo, Japan; 3 Biostatistics Unit, Clinical and Translational Research Center, Keio University Hospital, Tokyo, 160-8582 Japan; 4 Department of Bioregulation, Graduate School of Medicine, Nippon Medical School, Tokyo, 113-8602 Japan; 5 Division of Cancer Therapeutics, National Cancer Center Research Institute, Tokyo, 104-0045 Japan

**Keywords:** Network meta-analysis, Nonalcoholic fatty liver disease, Shear wave elastography, Transient elastography

## Abstract

**Purpose:**

To compare the diagnostic test accuracy (DTA) of shear wave elastography (SWE) to that of transient elastography (TE) for liver fibrosis grade assessment in nonalcoholic fatty liver disease adults.

**Methods:**

MEDLINE, The Cochrane Library, and Web of Science were searched. Inclusion criteria were primary studies examining DTA of TE, point SWE (pSWE), two-dimensional SWE (2D-SWE), or magnetic resonance elastography (MRE) with liver biopsy. Network meta-analysis was conducted using a Bayesian bivariate mixed-effects model.

**Results:**

For fibrosis grade 2 or higher, 15 studies with 25 observations (16 observations for TE, 1 for MRE, 4 for pSWE and 2D-SWE; 2,066 patients) were included; the pooled sensitivity and specificity were 0.79 (95% credible interval (CrI) 0.70–0.86; 95% prediction interval (PI) 0.36–0.96) and 0.73 (95% CrI 0.62–0.82; 95% PI 0.23–0.96) for TE, 0.68 (95% CrI 0.48–0.83; 95% PI 0.23–0.94) and 0.75 (95% CrI 0.53–0.88; 95% PI 0.24–0.97) for pSWE, 0.85 (95% CrI 0.70–0.93; 95% PI 0.40–0.98) and 0.72 (95% CrI 0.49–0.86; 95% PI 0.20–0.96) for 2D-SWE, respectively. The proportion of studies classified as unclear in QUADAS-2 was high, and the results were heterogeneous.

**Conclusion:**

2D-SWE could be recommended as TE is for liver fibrosis assessment.

The protocol of this systematic review and network meta-analysis has been registered in PROSPERO (CRD42022327249). All included primary papers have already been published and the information and data can be used freely.

**Supplementary Information:**

The online version contains supplementary material available at 10.1007/s00261-024-04546-8.

## Aims and introduction

Nonalcoholic fatty liver disease (NAFLD) is common worldwide. The estimated global prevalence of NAFLD was 25.2% in 2018 [1], and it was reported that 30% of NAFLD patients go on to develop nonalcoholic steatohepatitis (NASH) [2]. NAFLD is asymptomatic and the fact that few treatment options are available is the biggest concern. Identifying the intermediate/high risk group of NAFLD, and intervening in associated risk factors to prevent cirrhosis, is crucial. The clinical care pathway recommends blood tests followed by elastography, to detect fibrosis in NAFLD patients in the early stages [3]. Therefore, elastographic imaging techniques are becoming pivotal tools in the non-invasive quantitative assessment of fibrosis.

Transient elastography (TE) and shear wave elastography (SWE) is used in the clinical setting to detect liver fibrosis with ease, compared to liver biopsy (LB). TE is recommended in some guidelines [4, 5]. However, point SWE (pSWE) and 2-dimensional-SWE (2D-SWE) were marketed later than TE and so far, only 2 primary studies [6, 7] directly compared diagnostic performance of all three (TE, pSWE and 2D-SWE) in hospital setting. As such, evidence synthesis using meta-analysis (MA) alone would be inadequate for clinicians to understand differences in the diagnostic performance of all of these non-invasive methods. Considering the American Association for the Study of Liver Diseases (AASLD) guidance [8], elastographic imaging techniques are keys to detecting ‘at risk’ NASH patients in hospitals after finding patients with suspected NAFLD at a primary/non hepatology care. If SWE can be used as an alternative to TE, it could improve timely patient access to assessment for liver fibrosis.

Network meta-analysis (NMA) enables comparisons, with some assumptions, that were not made in previous studies as indirect evidence, as opposed to direct evidence that contrasts interventions in 1 study. This method has been used to compare diagnostic accuracy of urinary biological tests to diagnose non-invasive bladder cancer [9], biomarkers to detect pancreatic cancer [10], and imaging methods to assess ischemic stroke [11]. However, having conducted a search of the database, it appears that no study has been reported for ultrasonographic elastography in NAFLD patients.

In this study, the aim was to clarify whether the diagnostic accuracy of SWE particularly for significant liver fibrosis was similar to TE in adult NAFLD by quantifying differences using NMA in the hospital setting.

## Methods

This study was reported in accordance with the Preferred Reporting Items for Systematic Reviews and Meta-Analyses (PRIMSA)-the diagnostic test accuracy statement (Supplementary information [SI]1) [12], and the protocol was registered in the International Prospective Register of Systematic Reviews (PROSPERO) (CRD42022327249).

### Study selection and bias assessment

A systematic review was conducted from electronic bibliographic databases including MEDLINE, The Cochrane Library, and Web of Science. Studies published from January 2010 to May 2022 were included. Medical subject headings with combinations for the literature search were used as follows: defined diseases (nonalcoholic fatty liver disease, NAFLD, nonalcoholic steatohepatitis and NASH), and elastographic methods (elastography, transient elastography, TE, magnetic resonance elastography (MRE), MR elastography, MRE, shear wave elastography, SWE, acoustic radiation force impulse imaging and ARFI) (SI.2). Two reviewers (RY and TO) applied the eligibility criteria and selected studies independently using the PRISMA flow diagram [13]. When decisions differed, discussions were held on whether the studies should be included or not until an agreement was reached. When selected papers included patients from the same clinics and hospitals, papers were chosen to include the most studies possible conducted in a single country.

The 2 reviewers independently assessed and determined risk of bias in each study in the same way using Quality Assessment of Diagnostic Accuracy Studies (QUADAS-2) (http://www.bris.ac.uk/quadas/). Each question was categorized as yes, no, or unclear following discussion.

### Inclusion and exclusion criteria

As the purpose of our study, we extracted studies which included all of the following criteria: (a) adults with NAFLD diagnosed by LB as a gold standard; (b) use of elastographic imaging; (c) primary papers which conducted cohort or case–control studies; (d) duration of the diagnosis between elastographic imaging and LB no longer than 3 months considering a lifestyle change which might affect the results of the examinations; and (e) a 2-by-2 table could be constructed. Exclusion criteria were (a) under 18 years; (b) NAFLD patients with other causes of liver diseases; (c) NAFLD patients were not diagnosed by LB, or patients were pathologically diagnosed as a normal liver; (d) studies written in languages other than English and Japanese or unpublished, as well as reviews, case reports, gray literature or letters; (e) the time periods of the studies from the same hospital or clinic overlapped; and (f) NAFLD subgroup data was not gained from studies which analyzed as chronic liver disease or NAFLD patients with normal liver. We removed 1 inclusion criteria; primary papers published by middle- or high- income countries written in our registered PROSPERO because no study was excluded by the criteria.

### Data extraction

Data was extracted as follows: patient characteristics (age, sex, body mass index [BMI], and diabetes mellitus [DM]), liver biopsy fibrosis stages, success rate and the interquartile range/median value of each elastographic method, fibrosis stages with the cutoff, accuracy measures (sensitivity and specificity), and the numbers of patients in 2-by-2 tables (the numbers of true positive, false positive, false negative, and true negative in addition to the overall sample sizes). Fibrosis stages were divided into 5 stages by Brunt/Kleiner classification [14, 15]: F0, no fibrosis; F1, perisinusoidal or portal; F2, perisinusoidal and portal or periportal; F3, septal or bridging fibrosis; and F4, cirrhosis. Additional information about study design, methodology, and the prevalence of NAFLD in each study was also collected.

The count data used in MA was cell counts in 2-by-2 tables. Some were calculated from the extracted values of sensitivity and specificity because some literature did not report or misreported all the essential values. The 95% confidence intervals for the accuracy measures of included studies were re-calculated from the 2-by-2 tables using the exact binomial method.

### Outcome measures

The pre-specified primary outcomes to be synthesized were sensitivity and specificity of a diagnosis for ≥ F2. As a set of secondary outcomes, the same measures for ≥ F3 and ≥ F1 were also synthesized, which were added after the protocol was registered because of the large number of studies and its clinical importance. Cirrhosis was out of scope in this study.

### Statistical analysis

A Bayesian random-effects bivariate normal model was fitted to the data for each ultrasonographic method separately before a mixed-effects bivariate normal model for NMA for all ultrasonographic methods, including MRE, to estimate pooled sensitivity and specificity and corresponding 95% credible intervals (CrIs) and prediction intervals (PIs) and 95% credible and prediction regions. Note that 1 result per each study was included in separate MA while multiple results with varying thresholds from 1 study were included in NMA when available. The inclusion of multiple thresholds was possible because of the hierarchy in the NMA model including study-level random effects. Results with a cutoff chosen to be 90% of sensitivity or specificity were excluded, which resulted in the exclusion of 1 study from MA and NMA [6]. This exclusion was needed because such sensitivity or specificity would have biased upwards our pooled estimates even though we used the bivariate normal models to jointly treat them. Hierarchical summary receiver operating characteristic curves were also drawn (see SI.2 for detailed statistical methods, SI.3 and SI.4 for fitting/convergence results of our main and inconsistency models, respectively). Posterior and prediction distributions of differences in sensitivity and specificity were sampled to calculate posterior/prediction probabilities that the differences were equal to or greater than 0%. The same set of analyses was done using a −5% margin.

Model fit was visually assessed in plots of prediction distributions and observed data points. Heterogeneity was assessed by prediction distributions and a pre-specified sub-group analysis by running a meta-regression including a covariate for published countries of study for its common effect across the different methods. A leave-one-out analysis was done to determine whether there was any influential study and if the result was robust. To assess the consistency assumption, the design-by-treatment-interaction model was used adding the inconsistency parameters into the main model for ≥ F2 [16]. Characteristics that might influence accuracy measures were evaluated visually to check the homogeneity and transitivity assumption. Deeks’ funnel plots were used to assess publication bias [17]. In a post-hoc manner, prediction intervals by plugging in posterior means of variance parameters were generated to see whether prediction intervals resulted from the main analysis were influenced by the posterior uncertainty in variance parameters. Posterior medians were used for point estimates because it was anticipated that the accuracy measures skewed distributions, and equally-tailed intervals for 95% CrIs and PIs were chosen. All analyses were done by R (version 4.1.2) and Stan (version 2.21.0) through the rstan package. The datasets, Stan and R codes for model fitting are available (https://github.com/tetsuroda/dta_nma_nafld_2024).

## Results

### Features of included studies and bias

**Fig. 1:**
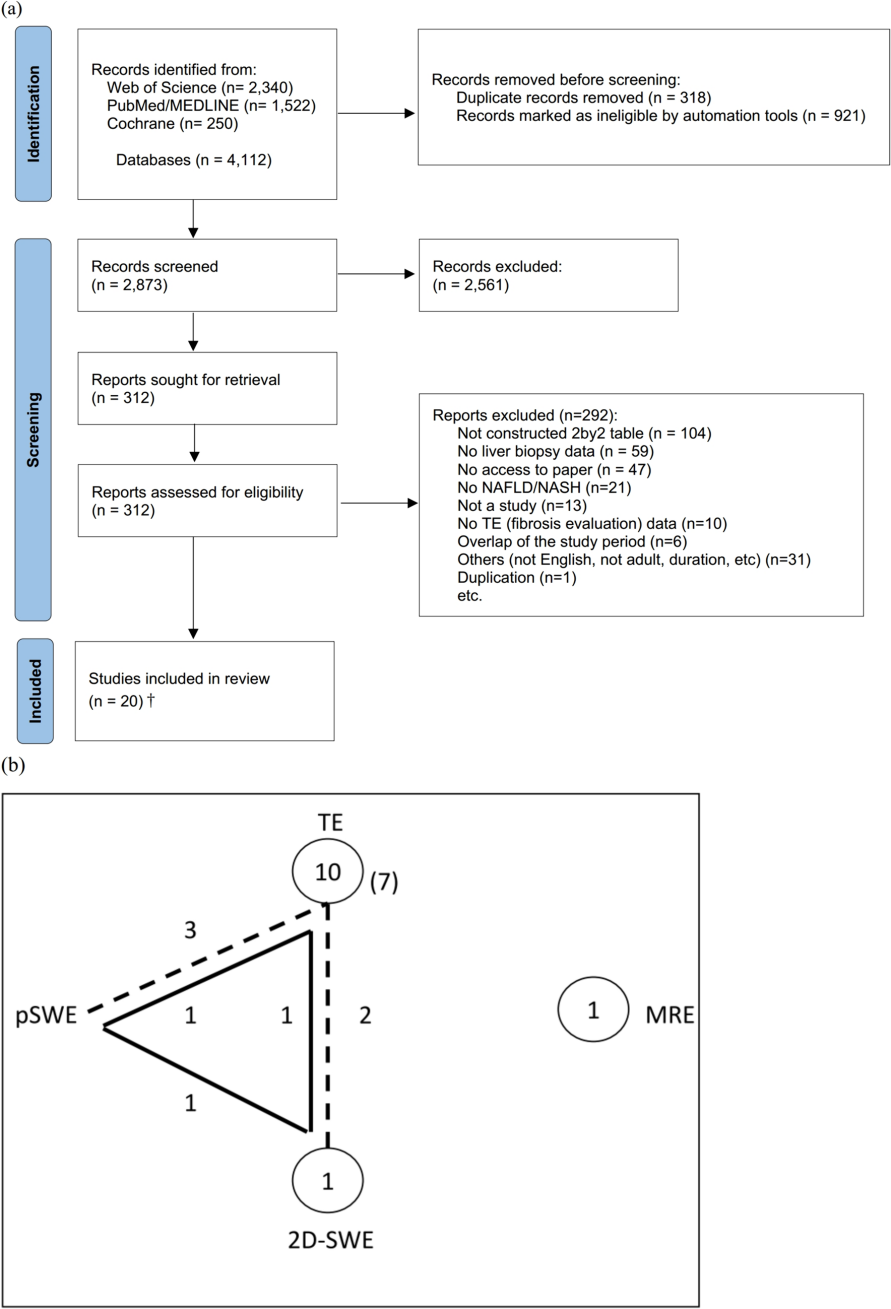
**a** Study selection from the PRISMA flow diagram, **b** network plots of the 4 elastographic methods. Fifteen studies with 25 observations for fibrosis stage ≥ 2. Vertices represent different echoic methods. Diagnoses connected by lines indicate a comparison in 1 study. Dashed lines represent studies comparing 2 diagnoses in 1 study whereas solid lines represent studies comparing 3 diagnoses in 1 study. Numbers indicate the number of comparisons made between the 2 vertices connected by the line. The numbers in circles represent the number of observations as a single diagnosis. Note that for TE, the numbers in circles include multiple results due to multiple thresholds for cutoff while the numbers in brackets show the number of studies. *MA* meta-analysis, *NMA* network meta-analysis, *MRE* magnetic resonance elastography, *pSWE* point shear wave elastography, *TE* transient elastography, *2D-SWE* two-dimensional shear wave elastography. ^†^One study was not included in MA and NMA because its cutoffs with 90% of sensitivity or with 90% of specificity were only reported in this study [6]

There were 4,112 studies primarily extracted, with 19 primary studies [7, 18–35] included in this review. Reasons and the number of studies excluded are shown in Fig. 1. The summary of each study and the bias of each study assessed by QUADAS-2 are shown in Table 1, Table S1 and Fig. S1 (SI.2), respectively. Three studies involved multiple hospitals [23, 31, 33]. The smallest number of patients in a study was 37 [35] and the largest was 251 [31]. Ten studies were from Asian countries [7, 19–23, 25, 27, 28, 31] and 7 studies were from European countries [18, 24, 26, 29, 30, 32, 35]. One was from the US [34] and Brazil [33]. All but 3 studies [28, 31, 34] conducted a prospective cohort study. In all studies [7, 18–35], the means or medians of age were middle ages. Most studies had a relatively even gender percentage [7, 19, 20, 23–32, 34, 35] but some included a higher percentage of men [18] or women [21, 22, 33]. The means or medians of BMI in all studies were above 25 [7, 18–35] and 7 studies were above 30 [20, 24, 30, 32–35]. Fig. S2 (SI.2) did not show trends between observed accuracy measures and characteristics, such as DM, cirrhosis, non-alcoholic steatohepatitis (NASH), LB needle gauge, LB methods (percutaneous or operation), and fasting before elastographic methods [36]. Seventeen studies conducted TE [7, 18–21, 24–35], 4 studies with pSWE [7, 20, 26, 29] and 2D-SWE [7, 23, 25, 30] and 2 studies with MRE [22, 35]. Ten studies only assessed the diagnostic accuracy of TE [18, 19, 21, 24, 27, 28, 31–34]. Three studies compared TE with pSWE [20, 26, 29], 2 studies TE with 2D-SWE [25, 30], 1 study with TE, pSWE and 2D-SWE [7], 1 study with 2D-SWE [23], MRE [22] or TE and MRE [35] (Table 1). All included studies used Brunt/Kleiner’s classification to assess fibrosis stages and were conducted in a hospital setting. Seven studies [18, 21, 24, 29–31, 33] were categorized in more than 2 categories as high in bias and applicability, especially studies which conducted TE in either category; patient selection, index test, reference standard and flow and timing. Other studies were assessed in most categories as low or unclear (Fig. S1 and Table S2 in SI.2).

**Table 1:** Characteristics of selected 19 papers

F stages	Author	Published year	Country	N (Pt/Hp)	Study design	Age (years)	Men (%)	BMI (kg/m^2^)	Obesity (%) BMI ≥ 25, ≥ 30	DM (%)	Cirrhosis (%)	NASH (%)	ALT (IU/L)	P/L, Gauge	Elastography/fasting (hours)
1, 2, 3	Lupşor et al. [18]^†^	2010	Romania	72/1	P, CS	42 (20–69)	70.8	28.7 (21.0–41.5)	NA, NA	NA	0.0	100.0	80 (15–343)	P, 14	TE: NA
1, 2, 3	Chan et al. [19]	2015	Malaysia	101/1	P, CS	50.3 ± 11.3	51.5	29.6 ± 3.9	87.1, NA	52.5	3.0	46.5	71 (44–115)	P, 18	TE: NA
1, 2, 3	Leong et al. [20]	2020	Malaysia	100/1	P, CS	57.1 ± 10.2	46.0	30.8 ± 4.8	92.0, NA	NA	4.0	80.0	36 (24–53)	P, 18	TE & pSWE: 2
1, 2, 3	Shi et al. [21]	2020	China	158/1	P, CS	48.9 ± 13.7	30.4	25.9 ± 3.2	57.0, NA	26.6	16.5	53.3	61 (34–110)	P, NA	TE: 3
1, 2, 3	Kim et al. [22]	2020	Korea	47/1	P, CS	51.0 ± 12.7	34.0	28.3 ± 6.2	NA, NA	NA	2.1	42.6	80 ± 43	P, 18	MRE: NA
1, 2, 3	Sugimoto et al. [23]	2020	Japan	111/M	P, CS	53.0 ± 18.0	51.4	27.2 ± 4.3	NA, NA	NA	13.5	81.1	80 ± 53	P, 16/18	2D-SWE: NA
1, 2, 3	Mikolasevic et al. [24]	2021	Croatia	179/1	P, CS	59 (50–67)	50.9	32.3 (29.3–37.2)	NA, NA	48.0	10.6	54.7	45 (30–67)	P, 16	TE: 3
1, 2, 3	Kuroda et al. [25]	2021	Japan	202/1	P, CS	55.2 ± 12.9	49.0	28.8 (25.7–33.3)	NA, NA	40.6	13.4	63.9	41 (21–69)	P, 14	TE and 2D-SWE: 4
1, 2, 3	Argalia et al. [26]	2022	Italy	50/1	P, CS	52.2 ± 13.0	64.0	29.4 ± 4.1	90.0, NA	NA	4.0	68.0	61 (52–69)	P, 18	TE & pSWE: 6
2, 3	Mahadeva et al. [27]	2013	Malaysia	131/1	P, CS	49.9 ± 12.3	52.7	NA	84.0, 32.8	47.3	6.1	54.9	NA	P, 18	TE: NA
2, 3	Loong et al. [28]	2017	China	215/1	R, CS	52 (41–57)	55.3	26.8 (24.8–29.3)	NA, NA	54.9	9.3	46.7	58 (37–85)	P, 16	TE: NA
2, 3	Lee et al. [7]	2017	Korea	94/1	P, CS	55.5 (52.9–58.1)	43.6	27.1 (26.4–27.9)	68.1, 25.5	39.4	14.9	31.9	50 (42–58)	P, NA	TE, pSWE & 2D-SWE: 2
2, 3	Taibbi et al. [29]	2021	Italy	46/1	P, CS	54.7 ± 9.1	58.7	29.4 ± 4.5	NA, NA	39.1	28.2	NA	NA^§^	NA, NA	TE: 4/pSWE: 2
2, 3	Mendoza et al. [30]	2022	Switzerland	104/1	P, CS	53.4 ± 12.6	58.7	30.9 ± 7.2	92.3, 56.7	47.1	7.7	82.7	76 ± 45	NA, NA	TE & 2D-SWE: NA
2, 3	Lee et al. [31]	2022	Korea	251/5	R, CS	44 (34–56)	52.6	28.6 (25.8–31.5)	81.7, 37.8	46.6^¶^	5.6	46.6	58 (33–110)	P, 19	TE: overnight
3	Labenz et al. [32]	2018	Germany	261/1	P, CS	51 (19–93)	52.5	30.9 (22.8–50.4)	NA, NA	29.9	0.0	48.7	60 (5–720)	P/L, 16	TE: NA
3	Tovo et al. [33]	2019	Brazil	104/2	P, CS	55.3 ± 10.0	26.0	33.0 ± 5.1	NA, NA	64.4	10.6	76.0	NA	P, NA	TE: 2
3	Trowell et al. [34]	2021	US	92/1	R, CS	55.3 ± 12.6	43.5	33.0 ± 5.72	NA, NA	43.5	24.0	72.8	56 ± 48	NA, NA	TE: 3
3	Troelstra et al. [35]	2021	Netherlands	37/1	P, CS^‡^	49.0 ± 13.2	62.2	33.2 ± 3.8	NA, NA	43.2	2.7	59.5	59 (47–79)	P, NA	MRE: 4/TE: 2

F stages, fibrosis stages analyzed in each study (1, ≥ F1; 2, ≥ F2; 3, ≥ F3) from Brunt/Kleiner

*N* number, *Pt* patients, *Hp* hospitals, *M* multiple hospitals, *P in study design* prospective study, *R* retrospective study, *CS* cross sectional study, *BMI* body mass index, *DM* diabetes mellitus, *NASH* nonalcoholic steatohepatitis, *ALT* alanine transaminase, *P* percutaneous, *L* laparoscopic, *Gauge* gauge size of liver biopsy, *NA* not applicable, *TE* transient elastography, *pSWE* point shear wave elastography, *2D-SWE* two-dimensional shear wave elastography, *MRE* magnetic resonance elastography

^†^Study population: NASH (others were NAFLD)

^‡^Longitudinal study

^§^Excluded patients with more than five times of aspartate transaminase (AST) and/or ALT level. ^¶^ Patients with DM or impaired fasting glucose. The figures of age, BMI, and ALT were reported as mean ± standard deviation or median (range) [those in [7] as mean (95% CI)]. [19]: using training cohort data

### Separate meta-analysis for ≥ F2

Thirteen, 4, and 4 studies for TE, pSWE and 2D-SWE were included in this analysis for ≥ F2 with 1,668, 279, and 470 patients, respectively. TE and 2D-SWE had a similar pooled sensitivity and specificity while pSWE had a relatively lower pooled sensitivity, although its specificity was similar to the others (Table S3, Figs. S3 and S4 in SI.2). Sensitivity and specificity were 0.79 (95% CrI 0.68–0.87; 95% PI 0.33–0.97) and 0.76 (95% CrI 0.66–0.84; 95% PI 0.35–0.95) for TE, 0.67 (95% CrI 0.37–0.86; 95% PI 0.12–0.97) and 0.74 (95% CrI 0.33–0.92; 95% PI 0.04–0.99) for pSWE, and 0.84 (95% CrI 0.63–0.92; 95% PI 0.34–0.97) and 0.71 (95% CrI 0.38–0.88; 95% PI 0.09–0.98) for 2D-SWE, respectively. The PIs were much wider than the corresponding CrIs, particularly for SWEs.

### Network meta-analysis for ≥ F2

**Fig. 2:**
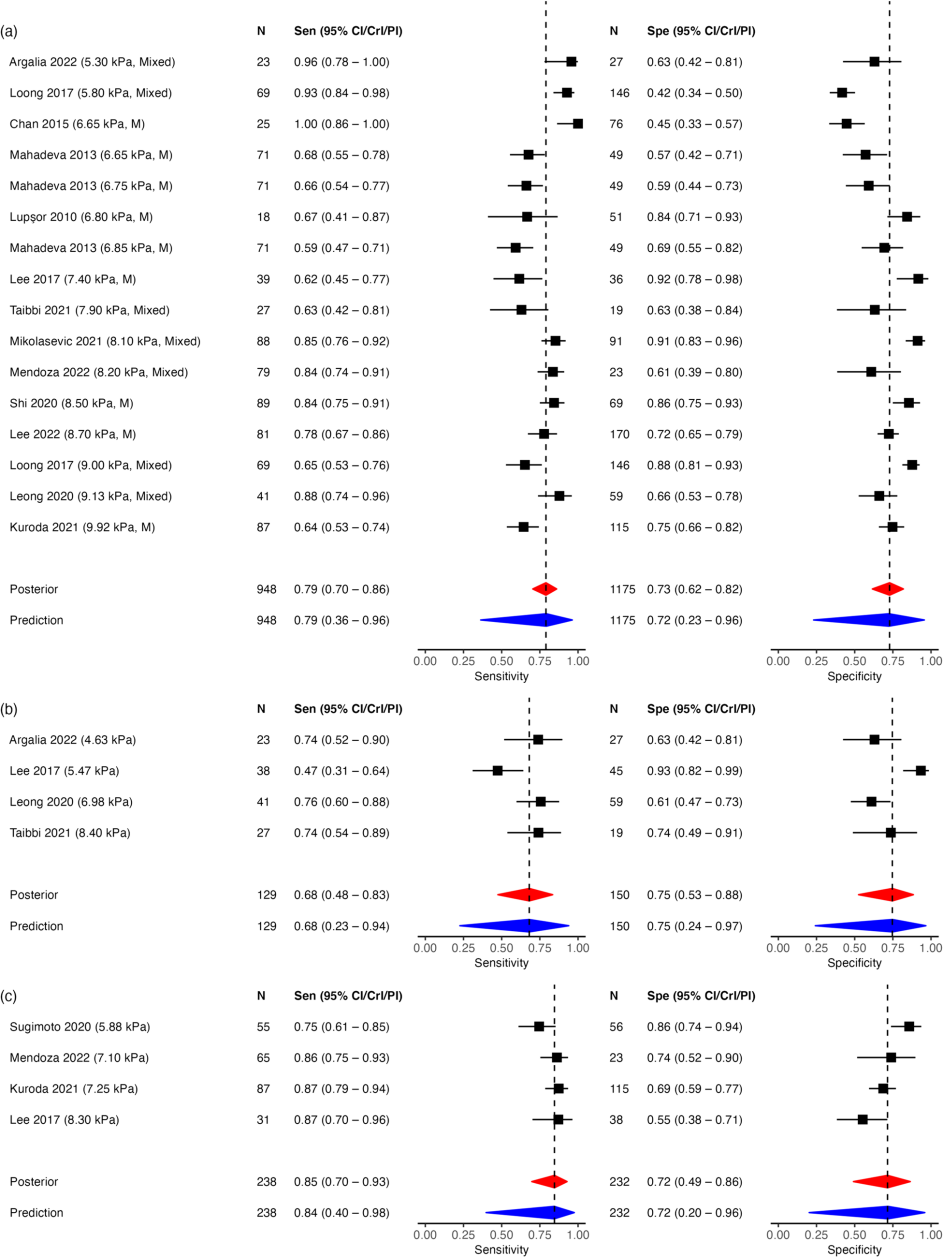
Forest plots of observed and pooled sensitivity and specificity values by network meta-analysis (Fibrosis stage ≥ 2). **a** TE, **b** pSWE, **c** 2D-SWE. The left column represents first authors, published year (cutoff values, probe size [only for TE; medium (M) or mixed of different sizes (Mixed)]). The 95% confidence intervals for the include studies were re-calculated from the data using the exact binomial method. Red diamonds indicate 95% credible intervals and blue diamonds indicate 95% prediction intervals. The dashed line is set at a posterior median. In each method, the studies are sorted with lower cutoff values to those with higher. *CI* confidence interval, *CrI* credible interval, *kPa *kilopascal, *N* number of patients included, *PI* prediction interval, *Sen* sensitivity, *Spe* specificity

**Fig. 3:**
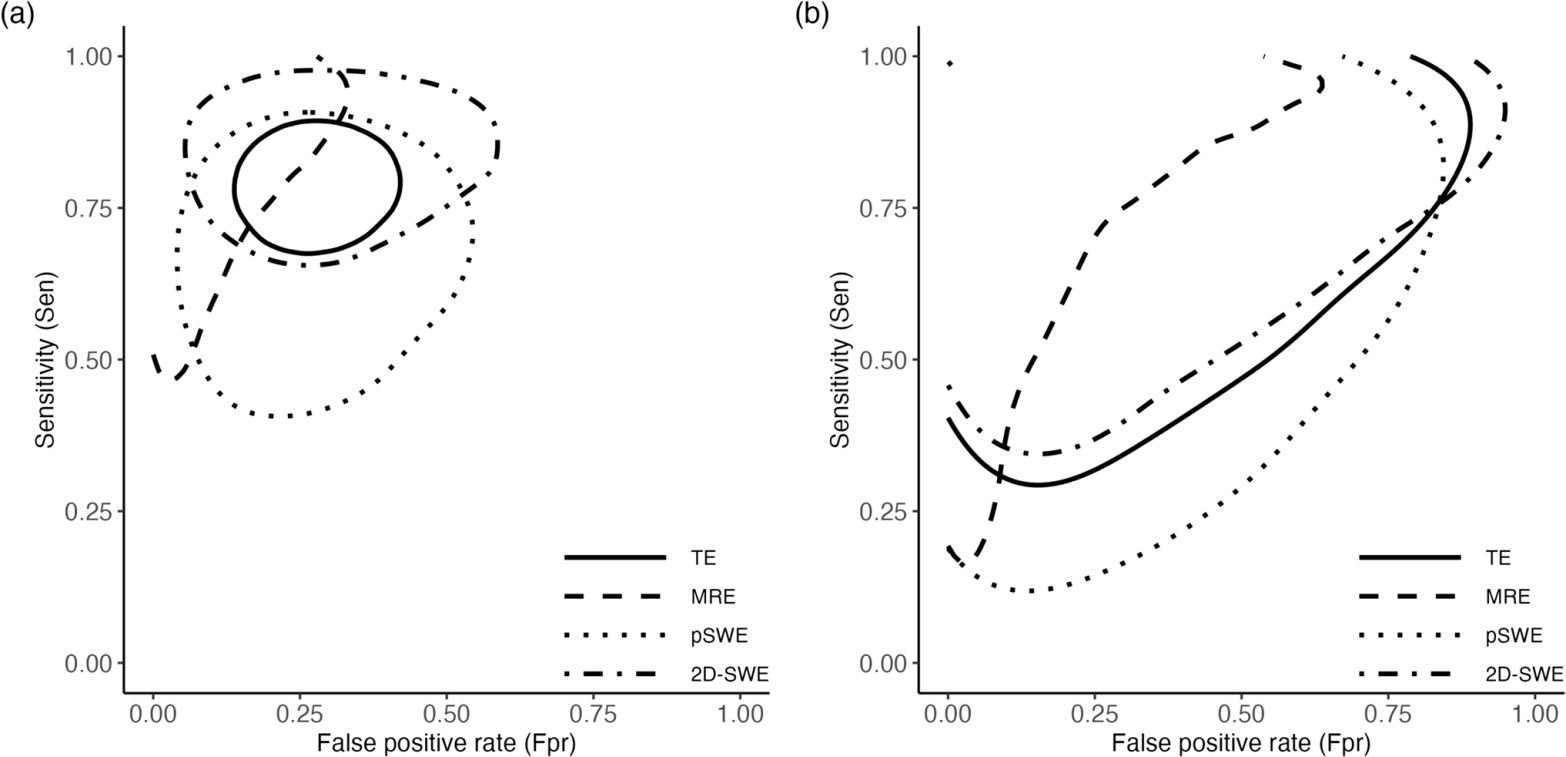
Overlayed 95% credible and prediction regions of each ultrasonographic method from the network meta-analysis: (**a**) 95% credible regions (**b**) 95% prediction regions. The solid line is TE. The dashed line is MRE. The dotted line is pSWE. The dot-dashed line is 2D-SWE. *Fpr* false positive rate, *MRE* magnetic resonance elastography, *pSWE* point shear wave elastography, *Sen* sensitivity, *TE* transient elastography, *2D-SWE* two-dimensional shear wave elastography

Fifteen studies with 25 observations (16 observations for TE, 1 for MRE, 4 for pSWE and 2D-SWE) with 2,066 patients were included in the NMA for ≥ F2. Network plots following Veroniki et al. [37] for ≥ F2 was shown in Fig. 1. Convergence diagnostics were detailed in SI.3. NMA results showed the same results as in the separate MA (Fig. 2), and SWEs had reduced uncertainty in posterior and prediction estimates, particularly in specificity (approximately 10 to 20 points), due to information borrowing across the hierarchy. However, predictions still implied heterogeneity. The posterior and prediction sensitivity and specificity were 0.79 (95% CrI 0.70–0.86; 95% PI 0.36–0.96) and 0.73 (95% CrI 0.62–0.82; 95% PI 0.23–0.96) for TE, 0.68 (95% CrI 0.48–0.83; 95% PI 0.23–0.94) and 0.75 (95% CrI 0.53–0.88; 95% PI 0.24–0.97) for pSWE, and 0.85 (95% CrI 0.70–0.93; 95% PI 0.40–0.98) and 0.72 (95% CrI 0.49–0.86; 95% PI 0.20–0.96) for 2D-SWE, respectively. MRE had the best accuracy among the 4 methods; sensitivity and specificity were 0.91 (95% CrI 0.58–0.99; 95% PI 0.40–0.99) and 0.96 (95% CrI 0.73–1.00; 95% PI 0.53–1.00). Fig. S5 (SI.2) showed that the 95% credible region for TE was narrower than the others, however, its 95% prediction region almost coincided with that for 2D-SWE, and that of pSWE covered the area of lower sensitivity compared to the others (Fig. 3).

All the 95% CrIs and PIs of pairwise differences in the accuracy measures comparing pSWE and 2D-SWE with TE covered 0% (Fig. S6 in SI.2). The league tables of the probability of pairwise difference in the posterior and prediction distributions of sensitivity and specificity equal to or greater than 0% or − 5% were shown in Figs. S7 and S8 (SI.2), respectively. In the prediction distribution of specificity, the probability greater than 0% was 53.2% between pSWE and TE (pSWE minus TE) and 48.8% between 2D-SWE and TE while in that of sensitivity, it was 32.5% between pSWE and TE and 61.1% between 2D-SWE and TE. When using a − 5% margin, the probability in the prediction distribution of difference in sensitivity between pSWE and TE increased to 41.3%, and that between 2D-SWE and TE was 72.0%.

The meta-regression model to check heterogeneity by studied countries yielded that the common odds ratio of non-Asian countries for sensitivity was 1.38 (95% CrI 0.61–3.16), and that for specificity was 1.16 (95% CrI 0.47–2.85). Table S4 (SI.2) showed the pooled accuracy measures in Asian and non-Asian countries, respectively.

Publication bias was not detected by Deeks’ funnel plots (Fig. S9 in SI.2). Forest plots of posterior and prediction estimates produced from the leave-one-out analysis were shown in Figs. S10 and S11 (SI.2). In 2 studies, the posterior/prediction estimates of SWEs appeared to be influenced more than the others, however, they did not change the major findings. With different priors, which are wider than those used in our main analysis, sensitivity analysis produced almost the same results as the main results except for MRE (Table S5, Figs. S11 and S12 in SI.2). MRE had 0.96 (95% CrI 0.67–1.00; 95% PI 0.49–1.00) for sensitivity and 1.00 (95% CrI 0.89–1.00; 95% PI 0.82–1.00) for specificity. The simpler model with no random-effects using the same prior distributions as the main analysis yielded equivalent results, though their 95% prediction intervals and regions were narrower compared to those in the main analysis (Table S5, Figs. S12 and S13 in SI.2). No large inconsistencies were detected in the main model because inconsistency parameters had wide 95% CrIs covering 0 and − 0.05, and the estimates were similar between consistency and inconsistency models in most parameters, though the pooled sensitivity of TE and the pooled specificity of 2D-SWE were lower than those in the consistency model (Table S6 in SI.2). The plug-in prediction intervals did not differ from those in the main analysis (Table S7 in SI.2).

### Synthesis of accuracy measures for ≥ F3 and ≥ F1

For ≥ F3 (19 studies with 35 observations [25 observations for TE, 2 for MRE, 4 for pSWE and 2D-SWE] with 2,460 patients) and ≥ F1 (9 studies with 12 observations [7 observations for TE, 1 for MRE, 2 for pSWE and 2D-SWE] with 1,017 patients), TE appeared to be slightly more accurate than the SWEs given the point estimates and their 95% prediction regions. For ≥ F1, pSWE had a roughly 10% lower sensitivity for both outcomes and a 10% lower specificity. Nonetheless, the probability of difference for both measures in the prediction distributions accounting for a − 5% margin indicated that the 3 methods were comparable at least in specificity (there was at least more than 40% prediction probability of pSWE or 2D-SWE had greater specificity than TE), and the results of corresponding sensitivity analyses were consistent (Table S8–S10 and Figs. S14–S33 in SI.2).

## Discussion

This study compared sensitivity and specificity of 4 elastographic methods for liver fibrosis in adult NAFLD patients by NMA. For ≥ F2, the diagnostic accuracy was comparable between TE and 2D-SWE given their 95% PIs and prediction regions, however, sensitivity of pSWE was slightly lower than the other 2 methods while specificity was similar. For ≥ F3 and ≥ F1, TE was slightly more accurate than SWEs, and pSWE had the lowest accuracy. Nonetheless, SWEs had relatively similar accuracy to TE in a prediction probability given a − 5% margin for all the outcomes, except for sensitivity of pSWE.

The purpose of potential ultrasonographic elastography use is to identify patients with “at risk” NASH [8] and to reduce unnecessary LB to patients without fibrosis at hepatology care after NAFLD patients screening with blood tests and/or abdominal ultrasonography in the general population at a primary/non-hepatology care. Two-dimensional SWE could be recommended as a diagnostic tool more officially in ≥ F2. Considering the increasing number of patients with NAFLD, it is reasonable to assess liver fibrosis at an earlier stage by TE and 2D-SWE as a choice of diagnostic methods because more facilities would then have the capability to assess fibrosis at an earlier stage. This would result in better management of NAFLD, even though MRE has the best accuracy when conducted in limited facilities.

Our results were consistent with some other existing MA studies comparing only part of all 4 elastographic methods: a MA with diagnostic accuracy of fibrosis stages to TE and pSWE [38], a MA to TE and SWE [39] and an individual participant data meta-analysis (IPDMA) comparing the diagnostic accuracy of TE and MRE [40]. Selvaraj’s review [41] compared to TE, pSWE, 2D-SWE and MRE by MA, and the aggregated diagnostic accuracy of TE was similar, but that of 2D-SWE was lower while that of pSWE was higher, compared to ours. These differences might come from study population in the present study; restricted patients with NAFLD adults not including a non-NAFLD population or other etiology of liver diseases; and the duration within 3 months between LB and elastography reducing influence of patients with lifestyle changes while 6 months are often employed. The stringent inclusion criteria of this study should reduce spectrum bias. The published year of included studies was also different and only literature [7] was included in both analyses. As such, we expected that the studies included in our analysis would be more homogenous and revealing of relevant data that could be applied to current practices.

The present study is the first NMA comparing the diagnostic accuracy of all available ultrasonographic methods of liver fibrosis. The NMA analysis enabled more precise estimation than the separate MA, though the number of studies included was relatively small due to stringent inclusion criteria. Our analysis also estimated the prediction intervals/regions that show a range of possible accuracy values in the future, which would be more useful than posterior estimates when clinicians think what level of accuracy would be obtained in the next diagnosis. The wider prediction intervals/regions indicated that any measures to reduce heterogeneity would be needed. In addition, probabilities where differences are equal to or greater than 0 or the margin allowed us more intuitive evaluation of accuracy compared to the dichotomized notion of null hypothesis testing because probability closer to 50% means the difference between the 2 diagnosis methods compared were due to random chance. Although careful interpretation of the results is needed, no obvious concerns have been found; characteristics of included studies’ samples appear to be relatively homogeneous within and between studies or the different diagnosis methods, and the consistency and inconsistency models provided similar results, as did the separate MA and a set of other sensitivity analyses.

This study does have some limitations. First, it was not possible to examine patient characteristics, which might affect the diagnostic accuracy, including the stages of steatosis and inflammation, DM and obesity. Further, the included studies had variations or unknown status (Table 1) in some characteristics including the type of probe (M and/or XL) [42] and transaminase level in TE study [43]. Therefore, the homogeneity and transitivity assumptions might not hold. However, the observed estimates of sensitivity and specificity against various factors (DM, cirrhosis, NASH, fasting hours, and needle gauge) did not show qualitatively obvious trends between them (Figs. S2, S15 and S26 in SI.2). We could not conduct a subgroup analysis for obesity because of its missingness, and we considered that the result obtained in the meta-regression between Asian and non-Asian countries would be a proxy of an analysis for obesity. However, it turned out to be inadequate because the average BMI values in the studies conducted in Malaysia were higher and close to those in non-Asian countries (Table S4 in SI.2). Considering the increase of obese patients in the world, it is becoming more important to screen such patients from the general population but there are currently few existing studies which clarify the features of this subgroup. In ultrasonographic elastography, the success rate of patients with BMI ≥ 30 kg/m^2^ decreased as BMI increased, and they showed the different trends; relatively young (43.3 ± 4.0 years), common in females, a lower percentage of ≥ F3, and a lower ALT level compared to non-obese NAFLD [6, 44]. It means that obesity patients need long-term follow-up, even though some have a normal ALT level, and the unreliable assessment of elastography might affect the adequate timing of the intervention. The recent metabolic dysfunction-associated steatotic liver disease diagnostic criteria includes BMI ≥ 25 kg/m^2^ (23 in Asia) and suspected or diagnosed DM [45], and we expected that further study might clarify the more detailed relationship of NAFLD with such factors and the diagnostic performance by using elastography, and what characteristics of NAFLD patients should require careful follow-up and treatment in primary and tertiary hospitals. Second, in the QUADAS assessment, some studies lack information on patient selection; failure rates of elastography; refusal rate of LB and the number of patients diagnosed pathologically as non-NAFLD, and/or lack criteria; the quality assessment of LB; diagnostic reliability [46–48] and diagnostic concordance among pathologists [48, 49]; and pre-specified thresholds of fibrosis stages for elastographic diagnosis. Such high-bias study settings and unclear information might be attributed to the heterogenous estimates and potentially the violation of homogeneity assumption although the leave-one-out analysis showed that there were no particular influential studies. Third, the heterogeneity might also come from our diffused prior distributions [50] though the sensitivity analysis yielded almost identical results, and the results might differ if each diagnosis method had uncommon heterogeneity unlike our assumption. Finally, we did not know the percentage of patients with cirrhosis who had been excluded from LB in the included studies. This caused selection bias and the results may be different when elastography is conducted in the general population. However, from the AASLD guidance [8], screening ‘at risk’ NASH is conducted only in hepatology care, not at a primary care level. Therefore, our results may be similar to clinical practice in hospitals. Further studies are needed to clarify whether the diagnostic accuracy of elastography is different in general population screening or hospital settings.

In conclusion, we conducted NMA to compare the diagnostic accuracy of 4 elastographic methods, and the results showed that 2D-SWE could be recommended as an alternative to TE for the assessment of liver fibrosis before LB. However, caution must be exercised in the use of pSWE because of low sensitivity. Further research is needed to reduce the heterogeneity in diagnostic accuracy, and to evaluate diagnostic accuracy for NAFLD.

## Supplementary Information

Below is the link to the electronic supplementary material.

Supplementary file1 (PDF 98 KB)

Supplementary file2 (DOCX 5065 KB)

Supplementary file3 (PDF 6698 KB)

Supplementary file4 (PDF 6326 KB)

## Data Availability

The datasets, Stan and R codes for model fitting are available at https://github.com/tetsuroda/dta_nma_nashnafld_2023. All primary papers included in this study are included in the reference section and the results are provided in this paper and in the supplementary information files. The protocol of this systematic review and network meta-analysis has been registered in PROSPERO (CRD42022327249). All included primary papers have already been published and the information and data can be used freely.

## Abbreviations

NAFLD: Nonalcoholic fatty liver disease
NASH: Nonalcoholic steatohepatitis
LB: Liver biopsy
TE: Transient elastography
SWE: Shear wave elastography
pSWE: Point shear wave elastography
2D-SWE: Two-dimensional shear wave elastography
MA: Meta-analysis
NMA: Network meta-analysis
MRE: Magnetic resonance elastography
BMI: Body mass index
DM: Diabetes mellitus
F: Fibrosis stages from Brunt/Kleiner classification
CrIs: Credible intervals
PIs: Prediction intervals
