# Supplementary material for: Comparison of the diagnostic accuracy of shear wave elastography with transient elastography in adult nonalcoholic fatty liver disease: a systematic review and network meta-analysis of diagnostic test accuracy
Source: Abdom Radiol (NY). 2024 Sep 6;50(2):734–46. doi: 10.1007/s00261-024-04546-8 (PMC11794403; doi:10.1007/s00261-024-04546-8)
Supplement: Supplementary file 2 — Supplementary file2 (DOCX 5065 KB) [file 261_2024_4546_MOESM2_ESM.docx]

**Comparison of the diagnostic accuracy of SWE with TE in adult NAFLD: a systematic review and network meta-analysis of diagnostic test accuracy.**

Table of Contents

[Supplementary Methods 2](#_Toc173687615)

[Meta-analysis and network meta-analysis 2](#_Toc173687616)

[Literature searching 8](#_Toc173687617)

[Supplementary Tables 9](#_Toc173687618)

[Results for ≥F2 12](#_Toc173687619)

[Results for ≥F3 15](#_Toc173687620)

[Results for ≥F1 16](#_Toc173687621)

[Supplementary Fig.s 17](#_Toc173687622)

[Result of bias assessment 17](#_Toc173687623)

[Results for ≥F2 18](#_Toc173687624)

[Results for ≥F3 29](#_Toc173687625)

[Results for ≥F1 39](#_Toc173687626)

# **Supplementary Methods**

## **Meta-analysis and network meta-analysis**

**Separate meta-analysis**

We fitted a Bayesian random-effects bivariate normal model for each diagnosis method, TE, pSWE, and 2D-SWE, to estimate pooled sensitivity and specificity and corresponding 95% credible intervals (CrIs) and prediction intervals (PIs) and 95% credible and predictive regions. This model assumes a bivariate normal distribution for sensitivity and specificity in a logit scale. This analysis was not performed for MRE because we included only one study examining MRE for the outcome of ≥F2. We also draw a hierarchical summary receiver operating characteristic (HSROC) curve and its 95% credible band for each diagnosis in a range of observed false positive rates, using the method proposed by Harbord et al[1]. Note that the most accurate results per diagnosis (the largest sum of sensitivity and specificity) in each study were included in the analysis. For ≥F1, separate meta-analysis was not conducted due to its small sample size.

**Network meta-analysis**

We performed a Bayesian network meta-analysis, adopting a mixed-effects bivariate normal model, to summarize the two accuracy measures using studies for TE, pSWE, 2D-SWE, and MRE. To evaluate quantitatively the similarity of accuracy among the different echoic methods, we sampled posterior and prediction distributions of differences in sensitivity and specificity to calculate posterior/prediction probabilities that the differences are equal to or greater than 0%. The probability of 50% means that the studies compared to each other have similar accuracy. We also did the same analysis using a -5% margin. To visualize the results, we produced league tables.

This method was a modified model of Owen et al. by replacing the fixed effects of a combination of diagnosis and threshold categories with that of diagnoses[2]. Diagnosis methods were included as a fixed effect, and studies and their interactions with diagnosis methods were included as random effects in a logit scale of the accuracy measures. Multiple thresholds can be modeled because of the study-level random effect. We did not adopt Owen's model because the cutoff values for diagnoses used in the included studies were not pre-specified values but those chosen by the Youden index. Therefore, we considered threshold effects would not be reasonably identifiable because cutoff values were likely related to patient characteristics or diagnosis techniques. Note that our analysis included multiple observations for each diagnosis per study, unlike the separate meta-analysis, because our model had random effects for studies and thresholds, and that we excluded results using thresholds set to have sensitivity or specificity of 90% as such accuracy results were considered to be qualitatively different from the others.

The covariance structure for the bivariate distribution was a common variance and correlation parameters across different types of diagnosis. All the random effects were modelled as independent normal distributions with mean 0 and different variance parameters.

**Details of NMA Models**

The first modelling assumption is that each of the numbers of true positive and true negative in tested positive and tested negative cases follow a binomial distribution, respectively, for an $i$-th observation:

$$Y_{pos, i}\sim Bin\left( {TP}_{i},P_{1, i} \right),$$

$$Y_{neg, i}\sim Bin\left( {TN}_{i},P_{2, i} \right),$$

where $P_{1,i}$ is sensitivity and $P_{2,i}$ is $1-specificity$. This way, we do not need to do continuity correction when there is/are zero count(s) because we directly modeled the probabilities, not by using cell counts in a two-by-two table.

We, next, assume that the logit-transformed $P_{1,i}$ and $P_{2,i}$ follows a bivariate normal distribution for an $i$-th observation in study $s_{i}$ investigating diagnosis method $t_{i}$:

$$\left[ \begin{matrix} \theta_{1,i} \\ \theta_{2,i} \end{matrix} \right]\sim N\left[ \left( \begin{matrix} \mu_{1,i} \\ \mu_{2,i} \end{matrix} \right),\left( \begin{matrix} \sigma_{se}^{2} & \rho\sigma_{se}\sigma_{sp} \\ \rho\sigma_{se}\sigma_{sp} & \sigma_{sp}^{2} \end{matrix} \right) \right],$$

$$\mathrm{where}\left[ \begin{matrix} \theta_{1,i} \\ \theta_{2,i} \end{matrix} \right]=\left[ \begin{matrix} \mathrm{logit}\left( P_{1, i} \right) \\ \mathrm{logit}\left( P_{2, i} \right) \end{matrix} \right] \mathrm{and},$$

$$\mu_{1,i}=\mu_{se,t_{i}}+\gamma_{0, se,s_{i}}+\gamma_{1, se,t_{i},s_{i}},$$

$$\mu_{2,i}=\mu_{sp,t_{i}}+\gamma_{0, sp,s_{i}}+\gamma_{1, sp,t_{i},s_{i}},$$

$$\mathrm{where} \gamma_{0, se,s_{i}}\sim N\left( 0,\tau_{0, se}^{2} \right), \gamma_{0, sp,s_{i}} \sim N\left( 0,\tau_{0, sp}^{2} \right),$$

$$\gamma_{1, se,s_{i}} \sim N\left( 0,\tau_{1, se}^{2} \right), \gamma_{1, sp,s_{i}}\sim N\left( 0,\tau_{1, sp}^{2} \right).$$

To improve the convergence, we used non-centered parameterization:

$$\gamma_{0, se,s_{i}}=\tau_{0, se}Z_{i}, \mathrm{where} Z_{i}\sim N\left( 0, 1 \right),$$

$$\gamma_{0, sp,s_{i}}=\tau_{0, sp}Z_{i}, \mathrm{where} Z_{i}\sim N\left( 0, 1 \right),$$

$$\gamma_{1, se,s_{i}}=\tau_{1, se}Z_{i}, \mathrm{where} Z_{i}\sim N\left( 0, 1 \right),$$

$$\gamma_{1, sp,s_{i}}=\tau_{1, sp}Z_{i}, \mathrm{where} Z_{i}\sim N\left( 0, 1 \right).$$

We did similar re-parameterization for the covariance matrix:

$$\left[ \begin{matrix} \theta_{1,i} \\ \theta_{2,i} \end{matrix} \right]=\left[ \begin{matrix} \mu_{1,i} \\ \mu_{2,i} \end{matrix} \right]+\left( \begin{matrix} \sigma_{se} & 0 \\ 0 & \sigma_{sp} \end{matrix} \right)L_{\Omega}Z_{i}, \mathrm{where} Z_{i}\sim N\left( 0, 1 \right),$$

$$\left( \begin{matrix} \sigma_{se}^{2} & \rho\sigma_{se}\sigma_{sp} \\ \rho\sigma_{se}\sigma_{sp} & \sigma_{sp}^{2} \end{matrix} \right)=\left( \begin{matrix} \sigma_{se} & 0 \\ 0 & \sigma_{sp} \end{matrix} \right)L_{\Omega}{L_{\Omega}}^{T}\left( \begin{matrix} \sigma_{se} & 0 \\ 0 & \sigma_{sp} \end{matrix} \right).$$

$L_{\Omega}$ is the Cholesky factors of the correlation matrix for the covariance matrix, which is of unit length.

For priors in both meta-analyses, we use wide priors as follows. A normal distribution with a mean of 0 and a standard deviation of 2 for $\mu_{1}$ and $\mu_{2}$. For all the variance parameters, we used a uniform distribution from 0 to 5; the prior for $\mu_{1}$ and $\mu_{2}$ covers from 2% to 98% with 95% probability. The uniform distribution almost covers the boundary between 0% and 100%. Lastly, for the Cholesky factor of the correlation matrix, we used a Lewandowski-Kurowicka-Joe (LKJ) correlation distribution with a shape parameter of 4. According to the Stan user guide, when the shape parameter $\eta=1$, the result is uniform correlations; when $\eta>1$ it favors less correlation and when $\eta<1$, it favors more correlation[3].

$$\mu_{1,i}, \mu_{2,i}\sim N\left( 0, 2 \right),$$

$$\sigma_{se},\sigma_{sp},\tau_{0,se}, \tau_{0,sp}, \tau_{1,se}, \tau_{1,sp}\sim U\left( 0, 5 \right),$$

$$L_{\Omega}\sim LkjCorr\left( 4 \right).$$

**Markov chain Monte Carlo sampling**

We used four Markov chains of 30,000 iterations after discarding 5,000 iterations while thinning by 10 iterations. In the sampling function of {rstan} package, we set 10 to the thinning argument, 0.99 to adapt_delta, and 12 to max_treedepth to eliminate possible divergence transitions[4]. The convergence of Markov chain Monte Carlo (MCMC) samples was checked by pairs plots, trace plots as well as autocorrelation plots. We also checked if the R hat was less than 1.01.

**Assessment of NMA model assumptions**

We visually assessed model fit in plots of predictive distributions and observed data points and assessed heterogeneity by predictive distributions. The prediction interval accounts for heterogeneity among studies, therefore, it can be interpreted as a distribution of future results given the model assumptions used. To investigate heterogeneity, pre-specified sub-group analysis was performed by running a meta-regression including a covariate for published countries of study for its common effect across the different methods. Other variables were listed as covariates in the planning stage, but we did not perform a meta-regression for them because they were very imbalanced among different levels, missingness, or group-level aggregated variables. The country of study could be a good aggregated variable summarizing the distributions of effect modifiers. We did a leave-one-out analysis to see if there was any influential study and if the result was robust. This analysis was also used to identify any study contributing to heterogeneity by comparing prediction intervals/regions before and after removing one study. We used the design-by-treatment-interaction model to assess the consistency assumption, adding the inconsistency parameters into the main model for ≥F2[5]. For the inconsistency model, we changed the parameters $\mu_{1,i}$ and $\mu_{2,i}$ in the main model to

$$\mu_{1,i}=\mu_{se,t_{i}}+\omega_{se,D_{i},t_{i}}+\gamma_{0, se,s_{i}}+\gamma_{1, se,t_{i},s_{i}}$$

and

$$\mu_{2,i}=\mu_{sp,t_{i}}+\omega_{sp,D_{i},t_{i}}+\gamma_{0, sp,s_{i}}+\gamma_{1, sp,t_{i},s_{i}},$$

where $\omega_{se,D_{i},t_{i}}$ and $\omega_{sp,D_{i},t_{i}}$ are inconsistency parameters for design $D_{i}=1,\ldots,4$ and diagnosis method $t_{i}$. We set the pSWE as the reference diagnosis method. For studies that do not include a reference diagnosis method, we applied a quasi-small dataset, 0.001 counts for a binary outcome, into the reference diagnosis method. From the network structure of the main model, we treated $D_{i}=1$ as a design containing TE, pSWE, and 2D-SWE, $D_{i}=2$ as TE and pSWE, $D_{i}=3$ as pSWE and 2D-SWE and $D_{i}=4$ as pSWE and MRE. For the reference diagnosis method $t_{i}=\mathrm{pSWE}$ and direct comparisons, we set $\omega_{se,D_{i},t_{i}}=0$ and $\omega_{sp,D_{i},t_{i}}=0$. For $D_{i}=4$, we omitted inconsistency parameters since pSWE and MRE had only an indirect comparison. Finally, our inconsistency model included $\omega_{se,2,\mathrm{TE}}$, $\omega_{sp,2,\mathrm{TE}}$, $\omega_{se,3,2D\text{-}\mathrm{SWE}}$, and $\omega_{sp,3,2D\text{-}\mathrm{SWE}}$.

The relationship between observed accuracy measures and characteristics that might influence them were also evaluated visually as to check the homogeneity and transitivity assumption. We used Deeks’ funnel plots to assess publication bias for each outcome[6].

**Sensitivity analyses**

We did (1) the same analysis using different priors and (2) the same analysis using a simpler model which does not contain random effects for the interaction between studies and diagnosis methods. As the different priors, we used a wider prior distribution; a normal distribution with a mean of 0 and standard deviation of 5 for mean parameters; a uniform distribution ranging 0 to 10 for variance parameters. An LKJ prior with a parameter of 6.

For ≥F1, the simpler model was used for the main network meta-analysis because the above model did not converge due to the small number of studies included.

**Convergence diagnostics**

The separate meta-analysis for pSWE and 2D-SWE had some divergence transitions; for ≥F2, 5% of included iterations and 1% of those for our sensitivity analysis had the transitions while for ≥F3, more or less 10% in the main analysis and 5% in the sensitivity analysis. Although, the transitions emerged across the samples, their effective sample sizes (ESSs) were larger than 400 (100 times four chains), and no parameters had $\hat{R}$ greater than 1.01, which is proposed as a condition to ensure that the transitions did not cause any problems. No divergence transitions appeared in the samples of network meta-analysis in both our main and sensitivity analysis for ≥F2 and ≥F3 though approximately 3% of iterations experienced divergence transitions in two analyses for ≥F1, namely, the main analysis using the simpler model or the sensitivity analysis using the different priors, respectively. Fitting results of the main and inconsistency model for ≥F2 and the main (simpler model) for ≥F1 are provided in the supporting information.

**Posterior and prediction regions**

The bivariate posterior and prediction regions were drawn as highest density regions by kernel density estimation using {ggdensity} package. To obtain smooth regions that are less likely to overfit, we chosen 3 for their adjust parameter in a post-hoc manner.

**References**

[1] Harbord RM, Deeks JJ, Egger M, Whiting P, Sterne JAC. A unification of models for meta-analysis of diagnostic accuracy studies. *Biostatistics* 2007; 8: 239–251.

[2] Owen RK, Cooper NJ, Quinn TJ, Lees R, Sutton AJ. Network meta-analysis of diagnostic test accuracy studies identifies and ranks the optimal diagnostic tests and thresholds for health care policy and decision-making. *J Clin Epidemiol* 2018; 99: 64–74.

[3] Stan Development Team. 1.13 Multivariate priors for hierarchical models | Stan User’s Guide, https://mc-stan.org/docs/stan-users-guide/multivariate-hierarchical-priors.html (2021, accessed 30 August 2023).

[4] Stan Development Team. Runtime warnings and convergence problems, https://mc-stan.org/misc/warnings.html (2022, accessed 30 August 2023).

[5] Jackson D, Barrett JK, Rice S, White IR, Higgins JPT. A design‐by‐treatment interaction model for network meta‐analysis with random inconsistency effects. *Stat Med* 2014; 33: 3639–3654.

[6] Deeks JJ, Macaskill P, Irwig L. The performance of tests of publication bias and other sample size effects in systematic reviews of diagnostic test accuracy was assessed. *J Clin Epidemiol* 2005; 58: 882–893.

## **Literature searching**

To regard with our literature search

Literature from January 2010 to May 2022 was collected through electronic databases,

Keywords for searching are as follows:

Disease:

Nonalcoholic liver disease (NAFLD), Nonalcoholic steatohepatitis (NASH)

Diagnosis methods:

elastography, transient elastography (TE), shear wave elastography (SWE), acoustic radiation force impulse (ARFI) imaging, or magnetic resonance elastography (MRE).

An example:

**Web of Science (1/5/2022)**

1 (non-alcoholic or nonalcoholic) adj (steatohepatitid* or liver*) (40,984)

2 nonalcoholic fatty liver (21,367)

3 NAFLD or NASH (85,705)

4 #1 OR #2 OR #3 (103,377)

5 magnetic resonance elastography or elastography or transient elastography or shear wave elastography (19,742)

6 TE or transient elastography (325,818)

7 SWE or share wave elastography (7,328)

8 acoustic radiation force impulse imaging or ARFI (2,303)

9 MRE or MR elastography or magnetic resonance elastography (9,509)

10 #5 OR #6 OR #7 OR #8 OR #9 (350,614)

11 #4 AND #10 (3,024)

12 #4 AND #10 and 2022 or 2021 or 2020 or 2019 or 2018 or 2017 or 2016 or 2015 or 2014 or 2013 or 2012 or 2011 or 2010 (2,340)

# **Supplementary Tables**

**Table S1: The reported sensitivity and specificity of the included studies.**

| Author, machine type  (Probe/company): N | FS | Cutoff  (kPa) | | N of  ≥ FS | N of  < FS | Sen  (%) | Spe  (%) | | Incl.  MA/ NMA |
| --- | --- | --- | --- | --- | --- | --- | --- | --- | --- |
| TE | | | | | | | | | |
| Lupşor 2010,  Fibroscan (M): 69 | F1 | 5.3 | 46 | | 23 | 0.93 | 0.78 | N/Y | |
|  | F2 | 6.8 | 18 | | 51 | 0.67 | 0.84 | Y/Y | |
|  | F3 | 10.4 | 5 | | 64 | 1.00 | 0.97 | Y/Y | |
| Mahadeva 2013,  Fibroscan (M): 120 | F2 | 6.7 | 71 | | 49 | 0.68 | 0.57 | N/Y | |
|  |  | 6.8 | 71 | | 49 | 0.66 | 0.60 | N/Y | |
|  |  | 6.9 | 71 | | 49 | 0.59 | 0.69 | Y/Y | |
|  | F3 | 7.0 | 27 | | 93 | 0.70 | 0.65 | N/Y | |
|  |  | 7.1 | 27 | | 93 | 0.70 | 0.67 | Y/Y | |
|  |  | 7.3 | 27 | | 93 | 0.67 | 0.68 | N/Y | |
| Chan2015,  Fibroscan 502 (M): 101 | F1 | 5.6 | 70 | | 31 | 0.90 | 0.42 | N/Y | |
|  | F2 | 6.7 | 25 | | 76 | 1.00 | 0.45 | Y/Y | |
|  | F3 | 8.0 | 19 | | 82 | 0.95 | 0.66 | Y/Y | |
| Casinotto 2016^†^,  Fibroscan (M/XL): 223 | F2 | 6.2 | 156 | | 67 | 0.90 | 0.45 | N/N | |
|  |  | 9.8 | 156 | | 67 | 0.60 | 0.90 | N/N | |
|  | F3 | 8.2 | 92 | | 131 | 0.90 | 0.61 | N/N | |
|  |  | 12.5 | 92 | | 131 | 0.57 | 0.90 | N/N | |
| Loong 2017,  Fibroscan (M/XL): 215 | F2 | 5.8 | 69 | | 146 | 0.93 | 0.42 | N/Y | |
|  |  | 9.0 | 69 | | 146 | 0.65 | 0.88 | Y/Y | |
|  | F3 | 7.9 | 43 | | 172 | 0.98 | 0.76 | Y/Y | |
|  |  | 9.6 | 43 | | 172 | 0.84 | 0.87 | N/Y | |
| Lee 2017,  Fibroscan (M): 75 | F2 | 7.4 | 39 | | 36 | 0.63 | 0.92 | Y/Y | |
|  | F3 | 8.0 | 23 | | 52 | 0.83 | 0.85 | Y/Y | |
|  |  | 11.7 | 23 | | 52 | 0.61 | 0.90 | N/N | |
| Labenz 2018,  Fibroscan402 (M/XL): 126 | F3 | 8.0 | 21 | | 105 | 0.91 | 0.70 | Y/Y | |
|  |  | 9.0 | 21 | | 105 | 0.81 | 0.74 | N/Y | |
|  |  | 10.0 | 21 | | 105 | 0.76 | 0.80 | N/Y | |
|  |  | 11.0 | 21 | | 105 | 0.52 | 0.83 | N/Y | |
|  |  | 12.0 | 21 | | 105 | 0.48 | 0.87 | N/Y | |
| Tovo 2019,  Fibroscan (M/XL): 104 | F3 | 7.9 | 20 | | 84 | 0.95 | 0.58 | N/Y | |
|  |  | 8.7 | 20 | | 84 | 0.90 | 0.64 | N/N | |
|  |  | 9.6 | 20 | | 84 | 0.85 | 0.69 | Y/Y | |
| Leong 2020,  Fibrscan (M/XL): 100 | F1 | 7.7 | 84 | | 16 | 0.83 | 0.81 | N/Y | |
|  | F2 | 9.1 | 41 | | 59 | 0.88 | 0.66 | Y/Y | |
|  | F3 | 9.3 | 33 | | 67 | 0.91 | 0.64 | Y/Y | |
| Shi 2020,  Fibroscan502,402 (M): 158 | F1 | 7.5 | 131 | | 27 | 0.71 | 0.89 | N/Y | |
|  | F2 | 8.5 | 89 | | 69 | 0.84 | 0.86 | Y/Y | |
|  | F3 | 10.8 | 54 | | 104 | 0.83 | 0.84 | Y/Y | |
| Trowell 2021,  Fibroscan 502(M/XL): 92 | F3 | 11.9 | 34 | | 58 | 0.85 | 0.69 | Y/Y | |
| Mikolasevic 2021,  Fibroscan502 (M/XL): 179 | F1 | 4.4 | 143 | | 36 | 0.90 | 0.17 | N/N | |
|  |  | 6.7 | 143 | | 36 | 0.75 | 0.92 | N/Y | |
|  |  | 8.1 | 143 | | 36 | 0.76 | 0.90 | N/N | |
|  | F2 | 5.8 | 88 | | 91 | 0.90 | 0.47 | N/N | |
|  |  | 8.1 | 88 | | 91 | 0.85 | 0.91 | Y/Y | |
|  |  | 8.1 | 88 | | 91 | 0.85 | 0.90 | N/N | |
|  | F3 | 9.5 | 43 | | 136 | 1.00 | 0.90 | N/N | |
|  |  | 10.0 | 43 | | 136 | 0.98 | 0.93 | Y/Y | |
|  |  | 10.6 | 43 | | 136 | 0.90 | 0.96 | N/N | |
| Kuroda 2021,  Fibroscan502 (M): 202 | F1 | 7.7 | 131 | | 71 | 0.60 | 0.79 | N/Y | |
|  | F2 | 9.9 | 87 | | 115 | 0.65 | 0.75 | Y/Y | |
|  | F3 | 11.9 | 55 | | 147 | 0.89 | 0.75 | Y/Y | |
| Taibbi 2021,  Fibroscan (M/XL): 46 | F2 | 7.9 | 27 | | 19 | 0.63 | 0.63 | Y/Y | |
|  | F3 | 8.5 | 18 | | 28 | 0.78 | 0.79 | Y/Y | |
| Troelstra 2021,  Fibroscan (M/XL): 37 | F3 | 9.9 | 8 | | 29 | 0.88 | 0.69 | Y/Y | |
| Mendoza 2022,  Fibroscan 502 (M/XL): 102 | F2 | 8.2 | 79 | | 23 | 0.83 | 0.62 | Y/Y | |
|  | F3 | 9.7 | 44 | | 58 | 0.73 | 0.53 | Y/Y | |
| Argalia 2022,  Fibroscan 402 (M/XL): 50 | F1 | 5.2 | 29 | | 21 | 0.90 | 0.67 | N/Y | |
|  | F2 | 5.3 | 23 | | 27 | 0.96 | 0.63 | Y/Y | |
|  | F3 | 8.8 | 8 | | 42 | 0.88 | 0.86 | Y/Y | |
| Lee 2022,  Fibroscan (M): 251 | F2 | 8.7 | 81 | | 170 | 0.78 | 0.72 | Y/Y | |
|  | F3 | 9.8 | 47 | | 204 | 0.96 | 0.79 | Y/Y | |
| pSWE | | | | | | | | | |
| Casinotto 2016^†^,  Acuson S2000  (Siemens): 236 | F2 | 2.7 | 160 | | 76 | 0.90 | 0.41 | N/N | |
|  |  | 5.2 | 160 | | 76 | 0.56 | 0.91 | N/N | |
|  | F3 | 4.0 | 93 | | 143 | 0.90 | 0.63 | N/N | |
|  |  | 7.0 | 93 | | 143 | 0.59 | 0.90 | N/N | |
| Lee 2017,  Acuson S2000 (Siemens): 83 | F2 | 5.0 | 38 | | 45 | 0.49 | 0.90 | N/N | |
|  |  | 5.5 | 38 | | 45 | 0.46 | 0.93 | Y/Y | |
|  | F3 | 5.6 | 20 | | 63 | 0.91 | 0.90 | N/N | |
|  |  | 6.1 | 20 | | 63 | 0.70 | 0.94 | Y/Y | |
| Leong 2020,  EPIQ7 (Philips): 100 | F1 | 6.8 | 84 | | 16 | 0.66 | 0.81 | N/Y | |
|  | F2 | 7.0 | 41 | | 59 | 0.76 | 0.61 | Y/Y | |
|  | F3 | 7.0 | 33 | | 67 | 0.76 | 0.58 | Y/Y | |
| Taibbi 2021,  RS80A (Samsung): 46 | F2 | 8.4 | 27 | | 19 | 0.74 | 0.74 | Y/Y | |
|  | F3 | 9.1 | 18 | | 28 | 0.72 | 0.79 | Y/Y | |
| Argalia 2022,  Affinity70 (Philips): 50 | F1 | 4.2 | 29 | | 21 | 0.83 | 0.57 | N/Y | |
|  | F2 | 4.6 | 23 | | 27 | 0.74 | 0.63 | Y/Y | |
|  | F3 | 7.4 | 8 | | 42 | 0.88 | 0.88 | Y/Y | |
| 2D-SWE | | | | | | | | | |
| Casinotto 2016^†^,  Aixplorer (SSI): 232 | F2 | 6.3 | 164 | | 68 | 0.90 | 0.50 | N/N | |
|  |  | 8.7 | 164 | | 68 | 0.71 | 0.90 | N/N | |
|  | F3 | 8.3 | 100 | | 132 | 0.91 | 0.71 | N/N | |
|  |  | 10.7 | 100 | | 132 | 0.71 | 0.90 | N/N | |
| Lee 2017,  Aixplorer (SSI): 69 | F2 | 8.3 | 31 | | 38 | 0.87 | 0.55 | Y/Y | |
|  |  | 20.5 | 31 | | 38 | 0.29 | 0.90 | N/N | |
|  | F3 | 10.7 | 20 | | 49 | 0.90 | 0.61 | Y/Y | |
|  |  | 23.2 | 20 | | 49 | 0.35 | 0.90 | N/N | |
| Sugimoto 2020,  Aplio i800 (Canon): 111 | F1 | 5.3 | 90 | | 21 | 0.64 | 0.90 | N/Y | |
|  | F2 | 5.9 | 55 | | 56 | 0.75 | 0.86 | Y/Y | |
|  | F3 | 5.9 | 40 | | 71 | 0.85 | 0.79 | Y/Y | |
| Kuroda 2021,  LOGIQE9XD (GE): 202 | F1 | 6.4 | 131 | | 71 | 0.83 | 0.66 | N/Y | |
|  | F2 | 7.3 | 87 | | 115 | 0.87 | 0.69 | Y/Y | |
|  | F3 | 8.4 | 55 | | 147 | 0.88 | 0.76 | Y/Y | |
| Mendoza 2022,  Aixplorer (SSI): 88 | F2 | 7.1 | 65 | | 23 | 0.86 | 0.73 | Y/Y | |
|  | F3 | 9.2 | 36 | | 52 | 0.65 | 0.86 | Y/Y | |
| MRE | | | | | | | | | |
| Kim2020,  (Siemens): 47 | F1 | 2.6 | 33 | | 14 | 0.97 | 1.00 | N/Y | |
|  | F2 | 3.1 | 20 | | 27 | 0.97 | 1.00 | N/Y | |
|  | F3 | 4.3 | 8 | | 39 | 1.00 | 0.92 | N/Y | |
| Troelstra 2021,  (Philips): 35 | F3 | 2.3 | 7 | | 28 | 1.00 | 0.79 | N/Y | |

Abbreviation: N, total number included in each study; FS, fibrosis stage; Sen, sensitivity; Spe, specificity; Incl, included; MA, meta-analysis; NMA, network meta-analysis; Y, Yes; N, no; TE, transient elastography; pSWE, point shear wave elastography; 2D-SWE, two-dimensional shear wave elastography; and MRE, magnetic resonance elastography.

All TE studies used Fibroscan® produced by Echosens, and M probe and/or XL probe.

† Not included in MA and NMA.

**Table S2: Details of the QUADAS-2 assessments in each study.** (A) Bias assessments of all studies in each category and (B) Applicability assessments of all studies in each category.

1. **(B)**

| **Author** | **PATIENT SELECTION** | **INDEX TEST** | **REFERENCE STANDARD** | **FLOW AND TIMING** | **Author** | **PATIENT SELECTION** | **INDEX TEST** | **REFERENCE STANDARD** |
| --- | --- | --- | --- | --- | --- | --- | --- | --- |
| **TE** | | | | | **TE** | | | |
| Argalia et al. | Low | Unclear | Unclear | Low | Argalia et al. | Low | Unclear | Unclear |
| Cassinotto et al. | Low | Unclear | Unclear | Unclear | Cassinotto et al. | Low | Unclear | Unclear |
| Chan et al. | Low | Low | Unclear | Unclear | Chan et al. | Low | Low | Unclear |
| Kuroda et al. | Low | Unclear | Low | Unclear | Kuroda et al. | Low | Unclear | Low |
| Labenz et al. | Unclear | Unclear | Unclear | Unclear | Labenz et al. | Unclear | Unclear | Unclear |
| Lee et al. (2017) | Low | Unclear | Unclear | Unclear | Lee et al. (2017) | Low | Unclear | Unclear |
| Lee et al. (2022) | High | Unclear | High | Unclear | Lee et al. (2022) | High | Unclear | High |
| Leong et al. | Low | Unclear | Unclear | Unclear | Leong et al. | Low | Unclear | Unclear |
| Loong et al. | Unclear | Low | Low | Unclear | Loong et al. | Unclear | Low | Low |
| Lupşor et al | Unclear | High | Unclear | Unclear | Lupşor et al. | Unclear | High | Unclear |
| Mahadeva et al. | Low | Unclear | Unclear | Unclear | Mahadeva et al. | Low | Unclear | Unclear |
| Mendoza et al. | Low | Unclear | High | Unclear | Mendoza et al. | Low | Unclear | High |
| Mikolasevic et al. | Unclear | High | Low | High | Mikolasevic et al. | Unclear | High | Low |
| Taibbi et al. | Low | High | Unclear | Unclear | Taibbi et al. | Low | High | Unclear |
| Tovo et al. | High | Low | Unclear | High | Tovo et al. | High | Low | Unclear |
| Troelstra et al. | Unclear | Unclear | Unclear | Unclear | Troelstra et al. | Unclear | Unclear | Unclear |
| Trowell et al. | Unclear | Unclear | Unclear | Unclear | Trowell et al. | Unclear | Unclear | Unclear |
| Shi et al. | Unclear | Unclear | High | Unclear | Shi et al. | Unclear | Unclear | High |
| **pSWE** | | | | | **pSWE** | | | |
| Argalia et al. | Low | Unclear | Unclear | Low | Argalia et al. | Low | Low | Low |
| Cassinotto et al. | Low | Unclear | Unclear | Unclear | Cassinotto et al. | Low | Low | Low |
| Taibbi et al. | Low | Unclear | Unclear | Unclear | Lee et al. (2017) | Low | Low | Low |
| Lee et al. (2017) | Low | Unclear | Unclear | Unclear | Leong et al. | Low | Low | Low |
| Leong et al. | Low | Unclear | Unclear | Unclear | Taibbi et al. | Low | Low | Low |
| **2D-SWE** | | | | | **2D-SWE** | | | |
| Cassinotto et al. | Low | Unclear | Unclear | Unclear | Cassinotto et al. | Low | Low | Low |
| Kuroda et al. | Low | Unclear | Low | Unclear | Kuroda et al. | Low | Low | Low |
| Lee et al. (2017) | Low | Unclear | Unclear | Unclear | Lee et al. (2017) | Low | Low | Low |
| Mendoza et al. | Low | Unclear | High | Unclear | Mendoza et al. | Low | Low | Low |
| Sugimoto et al. | Low | Unclear | Unclear | High | Sugimoto et al. | Low | Low | Low |
| **MRE** | | | | | **MRE** | | | |
| Kim et al. | High | Unclear | Unclear | Unclear | Kim et al. | Low | Low | Low |
| Troelstra et al. | Unclear | Unclear | Unclear | Unclear | Troelstra et al. | Low | Low | Low |

Abbreviation: TE, transient elastography; pSWE, point shear wave elastography; 2D-SWE, two-dimensional shear wave elastography; and MRE, magnetic resonance elastography.

## Results for ≥F2

**Table S3: Posterior and prediction medians and associated 95% credible intervals and prediction intervals in the separate meta-analyses (Fibrosis stage** ≥**2).**

| Method | Posterior sen | Posterior spe | Prediction sen | Prediction spe |
| --- | --- | --- | --- | --- |
| TE  (N = 13) | 0.79  (0.68 – 0.87) | 0.76  (0.66 – 0.84) | 0.78  (0.33 – 0.97) | 0.76  (0.35 – 0.95) |
| pSWE  (N = 4) | 0.67  (0.37 – 0.86) | 0.74  (0.33 – 0.92) | 0.67  (0.12 – 0.97) | 0.74  (0.04 – 0.99) |
| 2D-SWE  (N = 4) | 0.84  (0.63 – 0.92) | 0.71  (0.38 – 0.88) | 0.84  (0.34 – 0.97) | 0.71  (0.09 – 0.98) |

Abbreviation: CrIs, credible intervals; PIs, prediction intervals; TE, transient elastography; MRE, magnetic resonance elastography; pSWE, point shear wave elastography; 2D-SWE, two-dimensional shear wave elastography; sen, sensitivity; and spe, specificity.

N represents the number of studies included. 95% CrIs or PIs are expressed in the brackets for posterior or prediction estimates, respectively.

**Table S4: Pooled posterior medians of accuracy measures of studies conducted in Asian or non-Asian countries, estimated by the meta-regression model (Fibrosis stage** ≥**2).**

|  | Asian countries | Non-Asian countries |
| --- | --- | --- |
| Sensitivity | | |
| TE | 0.77 (0.66 – 0.85) | 0.82 (0.69 – 0.91) |
| pSWE | 0.64 (0.41 – 0.82) | 0.71 (0.48 – 0.87) |
| 2D-SWE | 0.83 (0.65 – 0.92) | 0.87 (0.69 – 0.95) |
| MRE | 0.89 (0.53 – 0.99) | 0.92 (0.57 – 0.99) |
| Specificity | | |
| TE | 0.72 (0.58 – 0.82) | 0.74 (0.56 – 0.87) |
| pSWE | 0.73 (0.49 – 0.88) | 0.75 (0.50 – 0.90) |
| 2D-SWE | 0.70 (0.46 – 0.86) | 0.73 (0.44 – 0.90) |
| MRE | 0.96 (0.66 – 1.00) | 0.96 (0.65 – 1.00) |

Abbreviation: TE, transient elastography; pSWE, point shear wave elastography; 2D-SWE, two-dimensional shear wave elastography; MRE, magnetic resonance elastography; and CrI, credible interval.

95% CrIs are expressed in the brackets.

**Table S5: Posterior and prediction medians and associated 95% credible intervals and prediction intervals in the separate meta-analyses and network meta-analysis (sensitivity analysis) (Fibrosis stage** ≥**2).**

| Method | Model | Posterior sen | Posterior spe | Prediction sen | Prediction spe |
| --- | --- | --- | --- | --- | --- |
| TE | Separate MA, different priors | 0.79  (0.68 – 0.88) | 0.76  (0.66 – 0.84) | 0.78  (0.33 – 0.97) | 0.76  (0.35 – 0.95) |
| (N = 16) | NMA, different priors | 0.79  (0.71 – 0.87) | 0.73  (0.62 – 0.82) | 0.79  (0.36 – 0.96) | 0.73  (0.26 – 0.96) |
|  | NMA, simpler model | 0.79  (0.71 – 0.85) | 0.73  (0.62 – 0.81) | 0.79  (0.43 – 0.95) | 0.73  (0.30 – 0.94) |
| pSWE | Separate MA, different priors | 0.68  (0.34 – 0.90) | 0.75  (0.26 – 0.96) | 0.68  (0.08 – 0.98) | 0.75  (0.02 – 1.00) |
| (N = 4) | NMA, different priors | 0.68  (0.47 – 0.84) | 0.76  (0.54 – 0.89) | 0.68  (0.22 – 0.94) | 0.75  (0.24 – 0.97) |
|  | NMA, simpler model | 0.68  (0.49 – 0.83) | 0.75  (0.54 – 0.88) | 0.68  (0.27 – 0.92) | 0.75  (0.29 – 0.96) |
| 2D-SWE | Separate MA, different priors | 0.84  (0.65 – 0.93) | 0.72  (0.36 – 0.93) | 0.84  (0.35 – 0.98) | 0.72  (0.08 – 0.99) |
| (N = 4) | NMA, different priors | 0.85  (0.70 – 0.93) | 0.72  (0.49 – 0.87) | 0.85  (0.44 – 0.98) | 0.72  (0.22 – 0.96) |
|  | NMA, simpler model | 0.85  (0.71 – 0.93) | 0.72  (0.52 – 0.86) | 0.85  (0.50 – 0.97) | 0.72  (0.26 – 0.95) |
| MRE | Separate MA, different priors | Not performed | Not performed | Not performed | Not performed |
| (N = 1) | NMA, different priors | 0.96  (0.67 – 1.00) | 1.00  (0.89 – 1.00) | 0.96  (0.49 – 1.00) | 1.00  (0.82 – 1.00) |
|  | NMA, simpler model | 0.92  (0.62 – 0.99) | 0.96  (0.74 – 1.00) | 0.92  (0.48 – 0.99) | 0.97  (0.61 – 1.00) |

Abbreviation: CrIs, credible intervals; PIs, prediction intervals; TE, transient elastography; pSWE, point shear wave elastography; 2D-SWE, two-dimensional shear wave elastography; MRE, magnetic resonance elastography; MA, meta-analysis; NMA, network meta-analysis; sen, sensitivity; and spe, specificity.

N represents the number of studies included. 95% CrIs or PIs are expressed in the brackets for posterior or prediction estimates, respectively.

**Table S6: The posterior medians and 95% credible intervals of inconsistency parameters and comparisons of pooled accuracy measures in the main (consistency) model and inconsistency model (Fibrosis stage** ≥**2).**

| Parameter | Main model | Inconsistency model |
| --- | --- | --- |
| Inconsistency parameters |  |  |
| $\boldsymbol{\omega}_{\boldsymbol{se,2,}\mathbf{TE}}$^†^ | NA | 0.59 (-0.45 – 1.68) |
| $\boldsymbol{\omega}_{\boldsymbol{se,3,}\mathbf{2D}\text{-}\mathbf{SWE}}$^‡^ | NA | -0.58 (-2.36 – 1.26) |
| $\boldsymbol{\omega}_{\boldsymbol{sp,2,}\mathbf{TE}}$^§^ | NA | -0.19 (-1.40 – 1.05) |
| $\boldsymbol{\omega}_{\boldsymbol{sp,3,}\mathbf{2D}\text{-}\mathbf{SWE}}$^¶^ | NA | 0.92 (-1.09 – 2.88) |
| Sensitivity |  |  |
| TE | 0.79 (0.70 – 0.86) | 0.71 (0.48 – 0.86) |
| pSWE | 0.68 (0.48 – 0.83) | 0.68 (0.46 – 0.83) |
| 2D-SWE | 0.85 (0.70 – 0.93) | 0.86 (0.68 – 0.94) |
| MRE | 0.91 (0.59 – 0.99) | 0.91 (0.55 – 0.99) |
| Specificity |  |  |
| TE | 0.73 (0.62 – 0.82) | 0.76 (0.51 – 0.90) |
| pSWE | 0.75 (0.53 – 0.89) | 0.74 (0.52 – 0.89) |
| 2D-SWE | 0.71 (0.49 – 0.86) | 0.66 (0.40 – 0.85) |
| MRE | 0.96 (0.71 – 1) | 0.96 (0.69 – 1.00) |

Abbreviation: CrIs, credible intervals; TE, transient elastography; pSWE, point shear wave elastography; 2D-SWE, two-dimensional shear wave elastography; se, sensitivity; sp, specificity; MRE, magnetic resonance elastography; and NA, not applicable.

95% CrIs are expressed in the brackets.

† The inconsistency parameter of TE sensitivity for a study design comparing TE and pSWE.

‡ The inconsistency parameter of 2D-SWE sensitivity for a study design comparing pSWE and 2D-SWE.

§ The inconsistency parameter of TE specificity for a study design comparing TE and pSWE.

¶ The inconsistency parameter of 2D-SWE specificity for a study design comparing pSWE and 2D-SWE.

**Table S7: Prediction intervals (PIs) obtained by plugging in posterior means of heterogeneity parameters (Fibrosis stage** ≥**2).**

| Method | Prediction sen | Prediction spe |
| --- | --- | --- |
| TE  (N = 16) | 0.79  (95% PI, 0.39 – 0.95) | 0.73  (95% PI, 0.28 – 0.95) |
| pSWE  (N = 4) | 0.68  (95% PI, 0.25 – 0.93) | 0.75  (95% PI, 0.27 – 0.96) |
| 2D-SWE  (N = 4) | 0.84  (95% PI, 0.44 – 0.97) | 0.71  (95% PI, 0.24 – 0.95) |

Abbreviation: TE, transient elastography; pSWE, point shear wave elastography; and 2D-SWE, two-dimensional shear wave elastography.

N represents the number of studies included. 95% PIs are expressed in the brackets for prediction estimates.

## Results for ≥F3

**Table S8: Posterior and prediction estimates and associated 95% credible intervals and prediction intervals in the separate meta-analyses (Fibrosis stage** ≥**3).**

| Method | Posterior sen | Posterior spe | Prediction sen | Prediction spe |
| --- | --- | --- | --- | --- |
| TE  (N = 17) | 0.89  (0.83 – 0.93) | 0.77  (0.69 – 0.83) | 0.89  (0.62 – 0.98) | 0.77  (0.43 – 0.93) |
| pSWE  (N = 4) | 0.75  (0.52 – 0.88) | 0.80  (0.39 – 0.95) | 0.75  (0.26 – 0.96) | 0.80  (0.05 – 1.00) |
| 2D-SWE  (N = 4) | 0.82  (0.49 – 0.94) | 0.76  (0.47 – 0.89) | 0.82  (0.14 – 0.99) | 0.76  (0.18 – 0.97) |

Abbreviation: CrIs, credible intervals; PIs, prediction intervals; TE, transient elastography; pSWE, point shear wave elastography; and 2D-SWE, two-dimensional shear wave elastography.

N represents the number of studies included. 95% CrIs and PIs are expressed in the brackets for posterior or prediction estimates, respectively.

**Table S9: Posterior and prediction estimates and associated 95 % credible intervals and prediction intervals in the separate meta-analyses and network meta-analysis (sensitivity analysis) (Fibrosis stage** ≥**3).**

| Method | Model | Posterior sen | Posterior spe | Prediction sen | Prediction spe |
| --- | --- | --- | --- | --- | --- |
| TE | Separate MA, different priors | 0.89  (0.84 – 0.93) | 0.77  (0.70 – 0.83) | 0.89  (0.63 – 0.98) | 0.77  (0.44 – 0.94) |
| (N = 25) | NMA, different priors | 0.87  (0.80 – 0.92) | 0.78  0.71 – 0.84) | 0.87  (0.41 – 0.99) | 0.78  (0.32 – 0.96) |
|  | NMA, simpler model | 0.86  (0.79 – 0.91) | 0.78  (0.71 – 0.83) | 0.86  (0.48 – 0.98) | 0.78  (0.45 – 0.94) |
| pSWE | Separate MA, different priors | 0.76  (0.52 – 0.91) | 0.82  (0.31 – 0.98) | 0.75  (0.26 – 0.97) | 0.82  (0.02 – 1.00) |
| (N = 4) | NMA, different priors | 0.77  (0.56 – 0.91) | 0.82  (0.67 – 0.92) | 0.77  (0.23 – 0.97) | 0.82  (0.36 – 0.98) |
|  | NMA, simpler model | 0.75  (0.56 – 0.88) | 0.81  (0.68 – 0.90) | 0.76  (0.29 – 0.96) | 0.81  (0.47 – 0.95) |
| 2D-SWE | Separate MA, different priors | 0.83  (0.46 – 0.96) | 0.77  (0.50 – 0.91) | 0.83  (0.11 – 1.00) | 0.77  (0.22 – 0.98) |
| (N = 4) | NMA, different priors | 0.84  (0.67 – 0.93) | 0.78  (0.61 – 0.89) | 0.84  (0.33 – 0.98) | 0.78  (0.29 – 0.97) |
|  | NMA, simpler model | 0.84  (0.69 – 0.93) | 0.77  (0.64 – 0.87) | 0.84  (0.41 – 0.98) | 0.77  (0.42 – 0.94) |
| MRE | Separate MA, different priors | Not performed | Not performed | Not performed | Not performed |
| (N = 2) | NMA, different priors | 1.00  (0.87 – 1.00) | 0.87  (0.64 – 0.96) | 1.00  (0.78 – 1.00) | 0.87  (0.40 – 0.99) |
|  | NMA, simpler model | 0.96  (0.73 – 1.00) | 0.85  (0.65 – 0.94) | 0.96  (0.57 – 1.00) | 0.85  (0.48 – 0.97) |

Abbreviation: CrIs, credible intervals; PIs, prediction intervals; TE, transient elastography; pSWE, point shear wave elastography; 2D-SWE, two-dimensional shear wave elastography; MRE, magnetic resonance elastography; MA, meta-analysis; NMA, network meta-analysis; sen, sensitivity; and spe, specificity.

N represents the number of studies included. 95% CrIs or PIs are expressed in the brackets for posterior or prediction estimates, respectively.

## Results for ≥F1

**Table S10: Posterior and prediction estimates and associated 95 % credible intervals and prediction intervals in the separate meta-analyses and network meta-analysis (sensitivity analysis) (Fibrosis stage** ≥**1).**

| Method | Model | Posterior sen | Posterior spe | Prediction sen | Prediction spe |
| --- | --- | --- | --- | --- | --- |
| TE | Separate MA, different priors | Not performed | Not performed | Not performed | Not performed |
| (N = 7) | NMA, different priors | 0.81  (0.62 – 0.92) | 0.78  (0.53 – 0.92) | 0.81  (0.22 – 0.98) | 0.78  (0.12 – 0.99) |
|  | NMA, simpler model^†^ | NA | NA | NA | NA |
| pSWE | Separate MA, different priors | Not performed | Not performed | Not performed | Not performed |
| (N = 2) | NMA, different priors | 0.73  (0.34 – 0.94) | 0.70  (0.25 – 0.94) | 0.73  (0.11 – 0.98) | 0.70  (0.06 – 0.99) |
|  | NMA, simpler model^†^ | NA | NA | NA | NA |
| 2D-SWE | Separate MA, different priors | Not performed | Not performed | Not performed | Not performed |
| (N = 2) | NMA, different priors | 0.77  (0.39 – 0.95) | 0.79  (0.39 – 0.97) | 0.77  (0.15 – 0.99) | 0.79  (0.11 – 0.99) |
|  | NMA, simpler model^†^ | NA | NA | NA | NA |
| MRE | Separate MA, different priors | Not performed | Not performed | Not performed | Not performed |
| (N = 1) | NMA, different priors | 0.97  (0.60 – 1.00) | 1.00  (0.68 – 1.00) | 0.97  (0.38 – 1.00) | 1.00  (0.42 – 1.00) |
|  | NMA, simpler model^†^ | NA | NA | NA | NA |

Abbreviation: CrIs, credible intervals; PIs, prediction intervals; TE, transient elastography; pSWE, point shear wave elastography; 2D-SWE, two-dimensional shear wave elastography; MRE, magnetic resonance elastography; MA, meta-analysis; NMA, network meta-analysis; sen, sensitivity; spe, specificity; and NA, not applicable.

N represents the number of studies included. 95% CrIs or PIs are expressed in the brackets for posterior or prediction estimates, respectively.

† Simpler model is the main model for ≥F1, therefore, it was not performed as a sensitivity analysis.

# **Supplementary Fig.s**

## Result of bias assessment


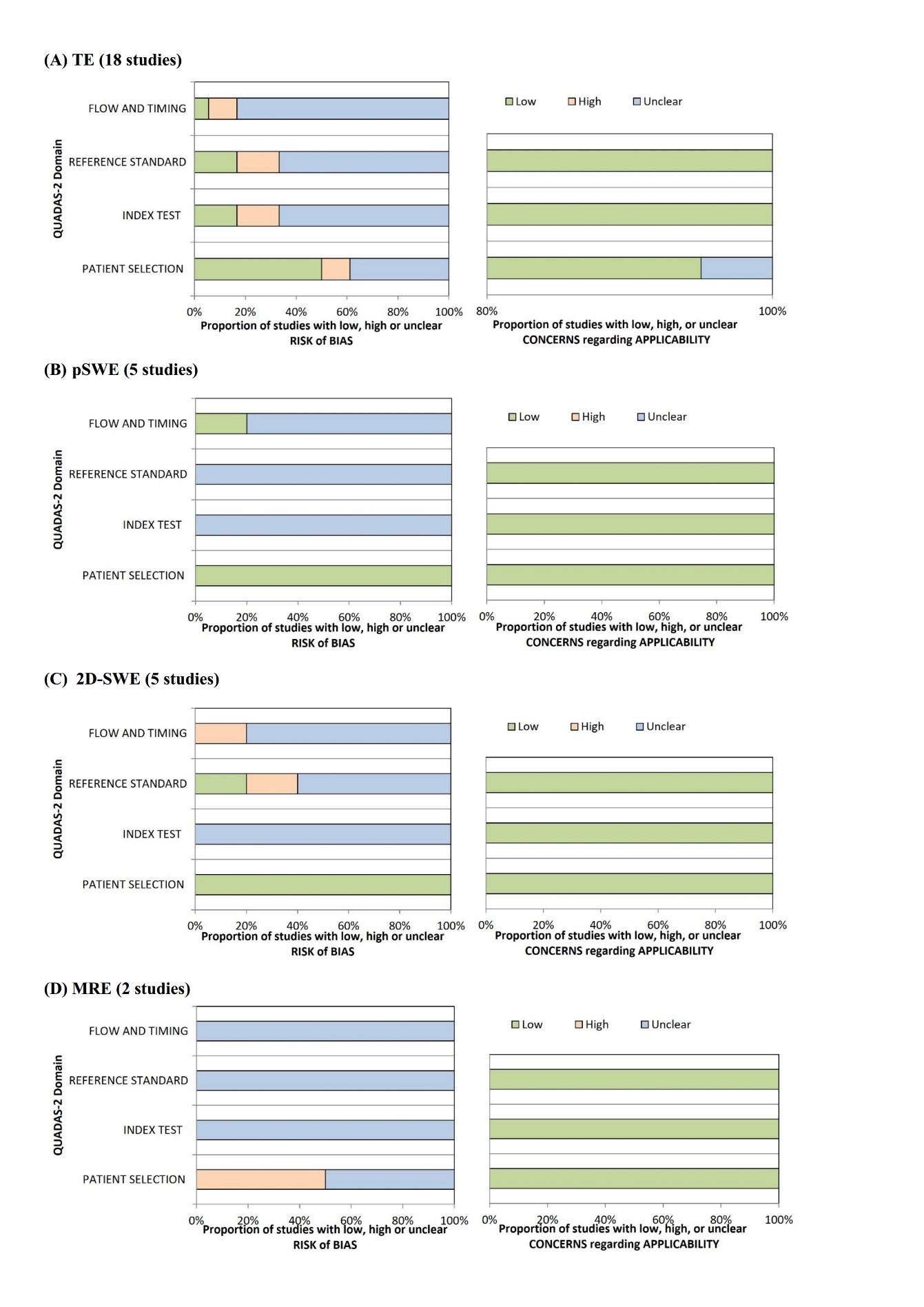


**Fig. S1: Summary of QUADAS-2 assessment.** (A) TE (18 studies), (B) pSWE (5 studies), (C) 2D-SWE (5 studies) and (D) MRE (2 studies). The colors, green, blue and orange, show the percentage of categorized low, unclear and high, respectively.

Abbreviation: TE, transient elastography; pSWE, point shear wave elastography; 2D-SWE, two-dimensional shear wave elastography; and MRE, magnetic resonance elastography.

## Results for ≥F2


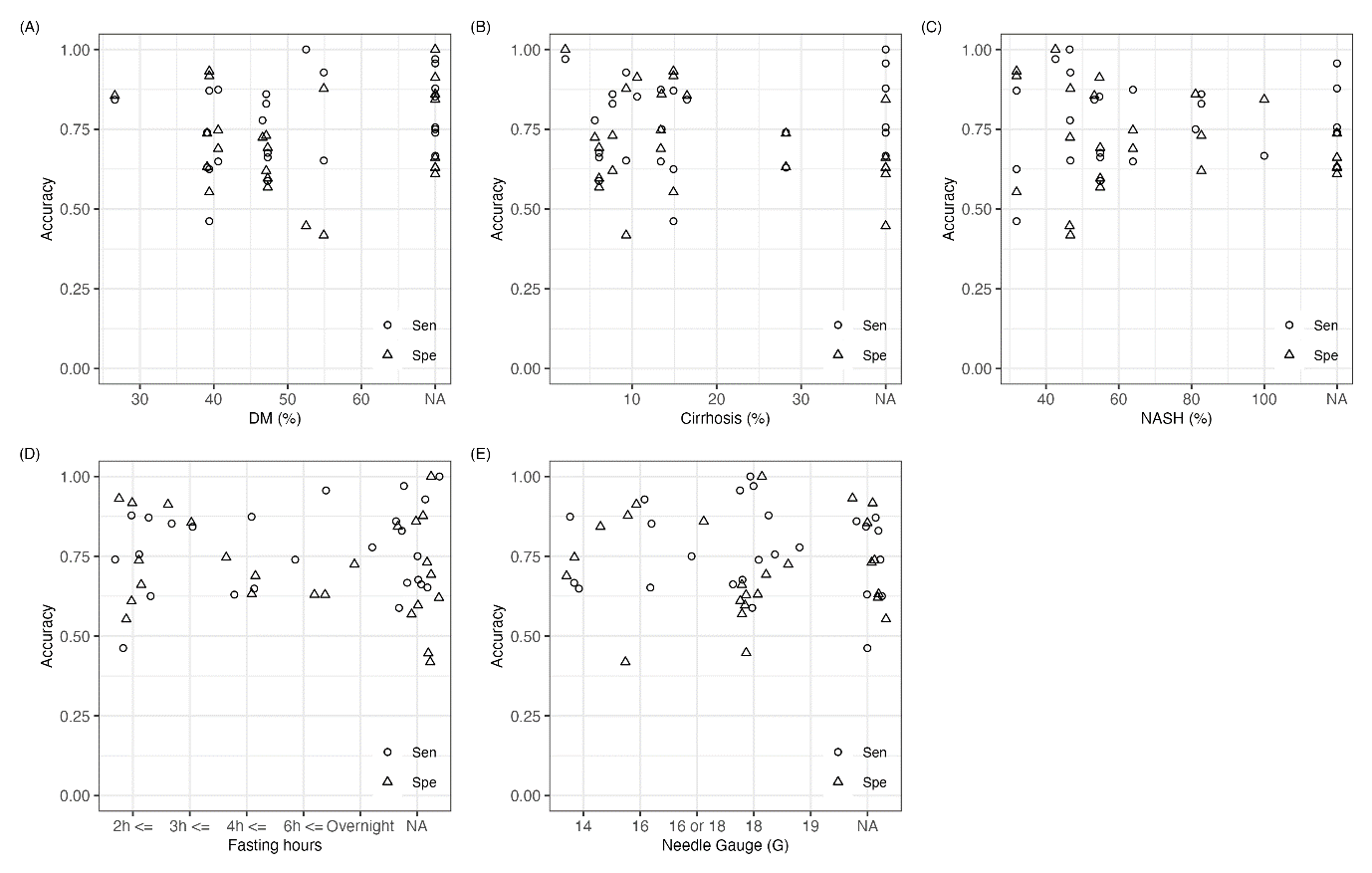


**Fig. S2:  Scattered plots of observed sensitivity and specificity of included studies over each characteristic (Fibrosis stage** ≥**2).**

Abbreviation: DM, diabetes mellitus; NASH, nonalcoholic steatohepatitis; h, hours; G, G needle gauge; Sen, sensitivity; Spe, specificity; and NA, not applicable.


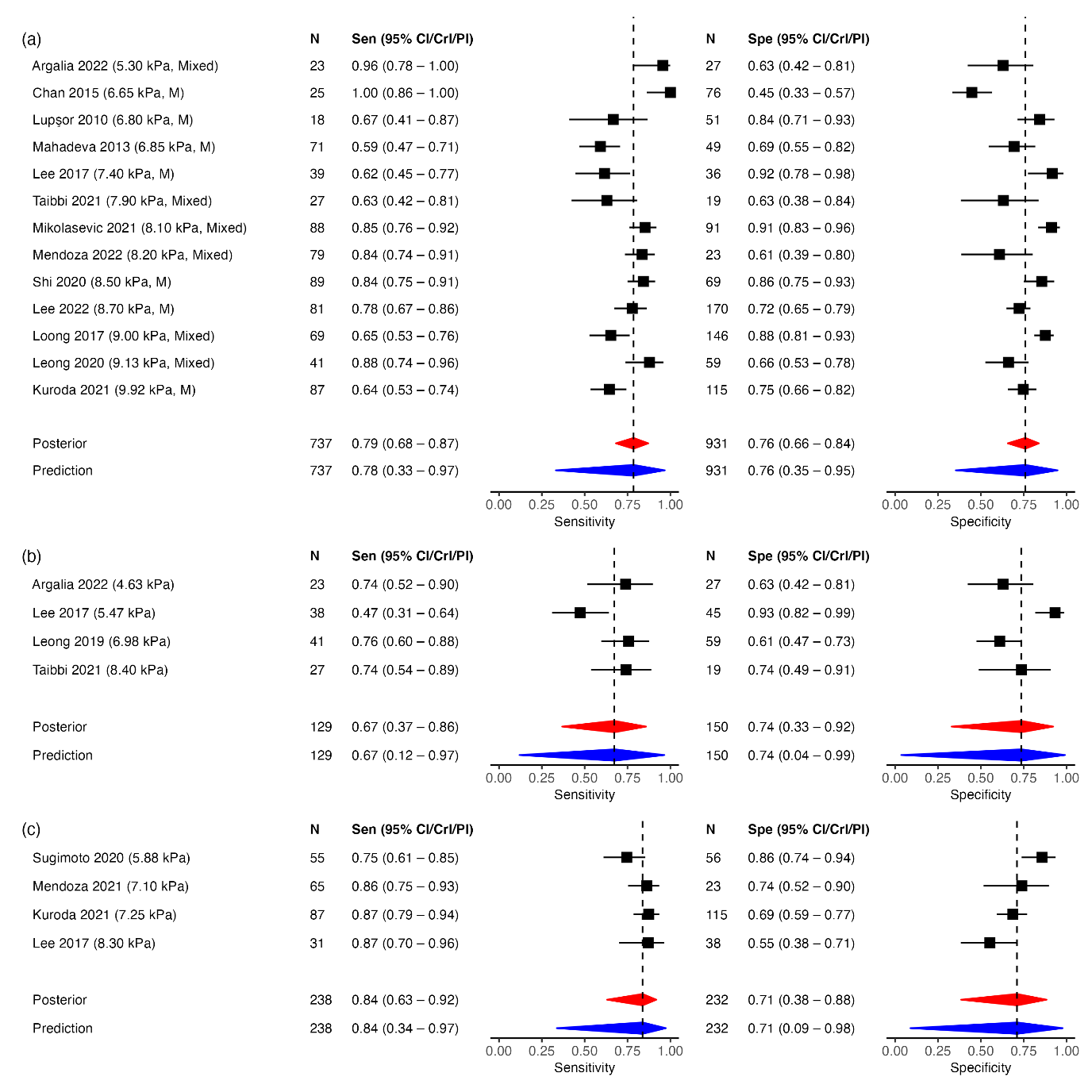


**Fig. S3: Forest plots of observed and pooled sensitivity and specificity values by separate meta-analysis (Fibrosis stage** ≥**2).** (A) TE (B) pSWE and (C) 2D-SWE. The left column represents first authors, published year (cutoff values, probe size [only for TE; medium (M) or mixed of different sizes (Mixed)]). The 95% confidence intervals for the include studies were re-calculated from the data using the exact binomial method. Red diamonds indicate 95% credible intervals and blue diamonds indicate 95% prediction intervals. The dashed line is set at a posterior median. In each method, the studies are sorted with lower cutoff values to those with higher.

Abbreviation: TE, transient elastography; pSWE, point shear wave elastography; 2D-SWE, two-dimensional shear wave elastography; Sen, sensitivity, Spe, specificity; CI, confidence interval; CrI, credible interval; and PI, prediction interval.


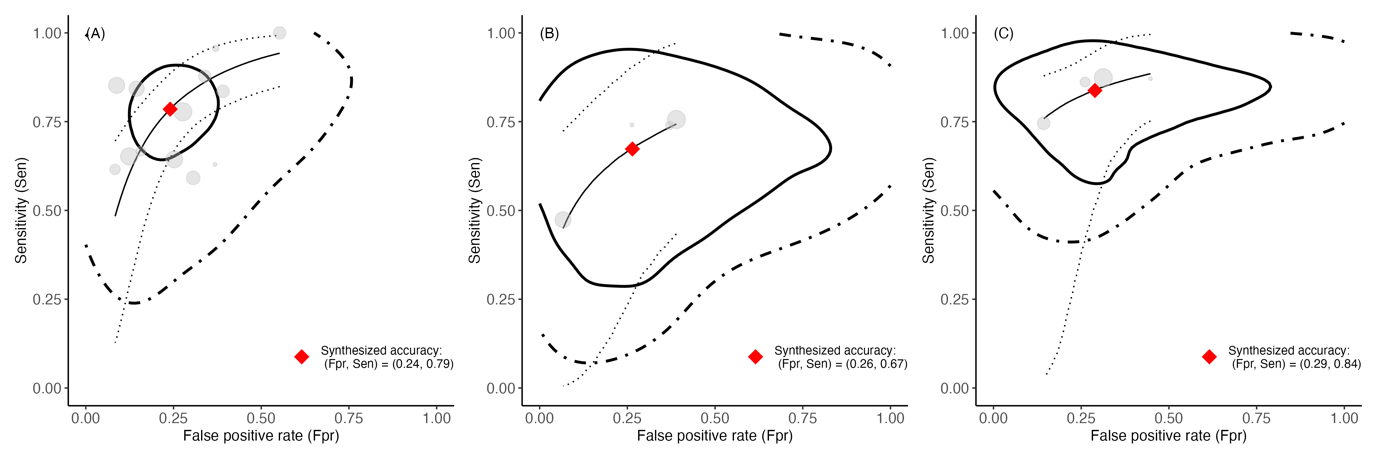


**Fig. S4: Hierarchical Summary Receiver Operator Characteristic (HSROC) curves with 95% credible and prediction intervals and regions from separate meta-analysis for each echoic method (Fibrosis stage** ≥**2).** (A) TE, (B) pSWE, and (C) 2D-SWE. The posterior estimates represent medians of sensitivity and false positive rate. The dotted grey bands show 95% credible intervals of the HSROC curve. The solid black lines show 95% credible regions. The dot-dashed lines show 95% prediction regions. The grey bubbles indicate observed data points with the bubble size proportional to the root of sample size of each study.

Abbreviation: TE, transient elastography; pSWE, point shear wave elastography; 2D-SWE, two-dimensional shear wave elastography; Sen, sensitivity; and Fpr, false positive rate.

**Fig. S5: 95% credible and prediction regions from the network meta-analysis for each echoic method (Fibrosis stage** ≥**2).** The posterior estimates represent medians of sensitivity and false positive rate. The solid black lines show 95% credible regions. The dashed lines show 95% prediction regions. The gray bubbles indicate observed data points with the bubble size proportional to the root of sample size of each study.

Abbreviation: TE, transient elastography; MRE, magnetic resonance elastography; pSWE, point shear wave elastography; 2D-SWE, two-dimensional shear wave elastography; Sen, sensitivity; and Fpr, false positive rate.


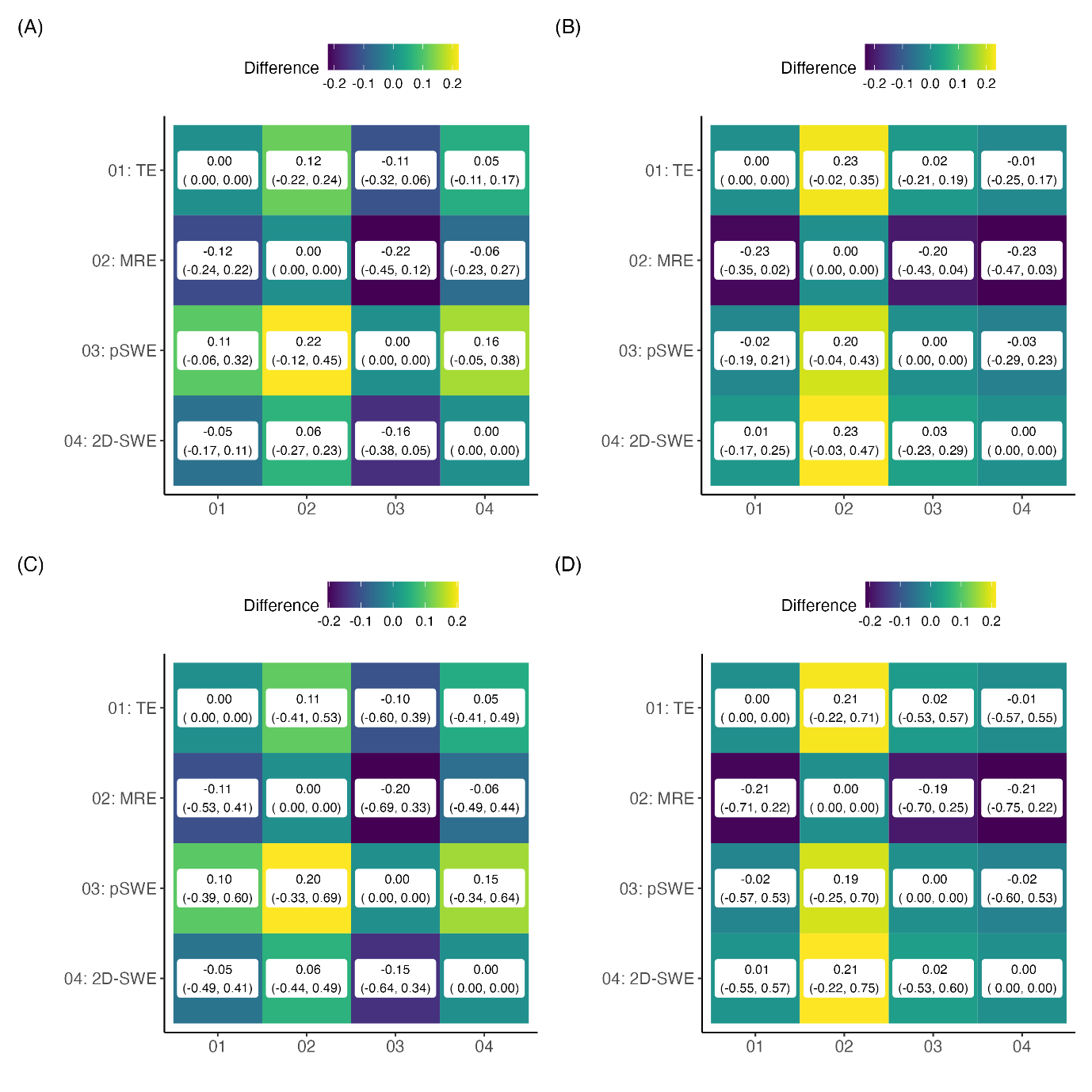
 **Fig. S6: League tables of the pairwise differences in sensitivity and specificity in posterior and predictive distributions for the main network meta-analysis (Fibrosis stage** ≥**2).** Each cell reads the column method minus row method. (A) Posterior, sensitivity (B) Posterior, specificity (C) Prediction, sensitivity and (D) Prediction, specificity.

Abbreviation: TE, transient elastography; MRE, magnetic resonance elastography; pSWE, point shear wave elastography; and 2D-SWE, two-dimensional shear wave elastography.

**Fig. S7: League tables of probabilities (%) being the pairwise differences are equal to or greater than a margin in sensitivity in posterior and predictive distributions obtained from the network meta-analysis:** Each cell reads the column method minus the row method. (A) Posterior, margin = 0% (B) Posterior, margin = −5% (C) Prediction, margin = 0% and (D) Prediction, margin = −5%.

Abbreviation: TE, transient elastography; MRE, magnetic resonance elastography; pSWE, point shear wave elastography; and 2D-SWE, two-dimensional shear wave elastography.

**Fig. S8: League tables of probabilities (%) being the pairwise differences are equal to or greater than a margin in specificity in posterior and predictive distributions obtained from the network meta-analysis:** Each cell reads the column method minus the row method. (A) Posterior, margin = 0% (B) Posterior, margin = −5% (C) Prediction, margin = 0% and (D) Prediction, margin = −5%.

Abbreviation: TE, transient elastography; MRE, magnetic resonance elastography; pSWE, point shear wave elastography; and 2D-SWE, two-dimensional shear wave elastography.


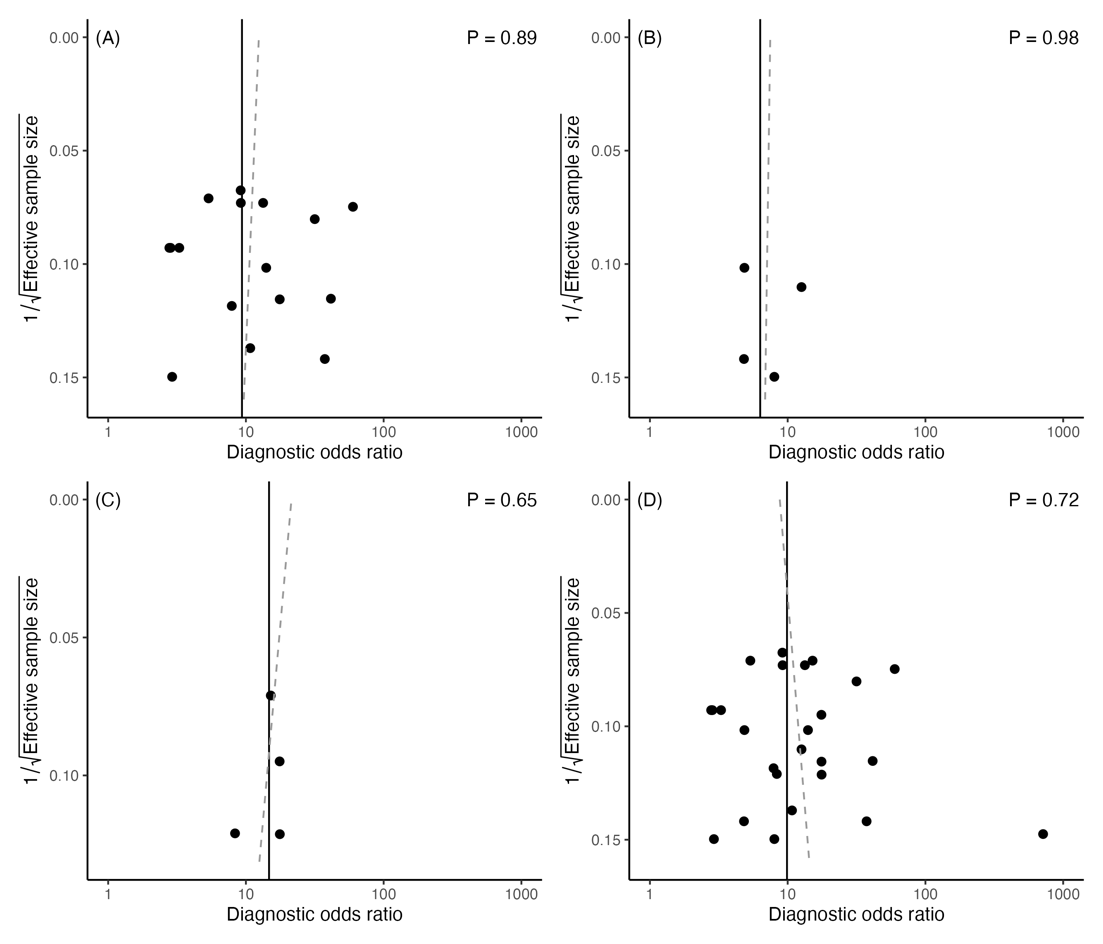


**Fig. S9: Deeks’ funnel plots with P-values from their asymmetry tests (Fibrosis stage** ≥**2).** (A) TE (B) pSWE (C) 2D-SWE and (D) All. The dot represents each observation. The vertical solid line represents the pooled means of a random-effects model. The dashed grey line represents the weighted regression line of log (diagnostic odds ratio) on the inverse of root of effective sample size (ESS) with ESS as weights. Continuity correction was used for all cell counts used. Two-sided P-value, which is calculated using the slope divided by its standard error following a t distribution with number of studies - 2 degrees of freedom, is noted at the top right in each graph.

Abbreviation: TE, transient elastography; pSWE, point shear wave elastography; and 2D-SWE, two-dimensional shear wave elastography.


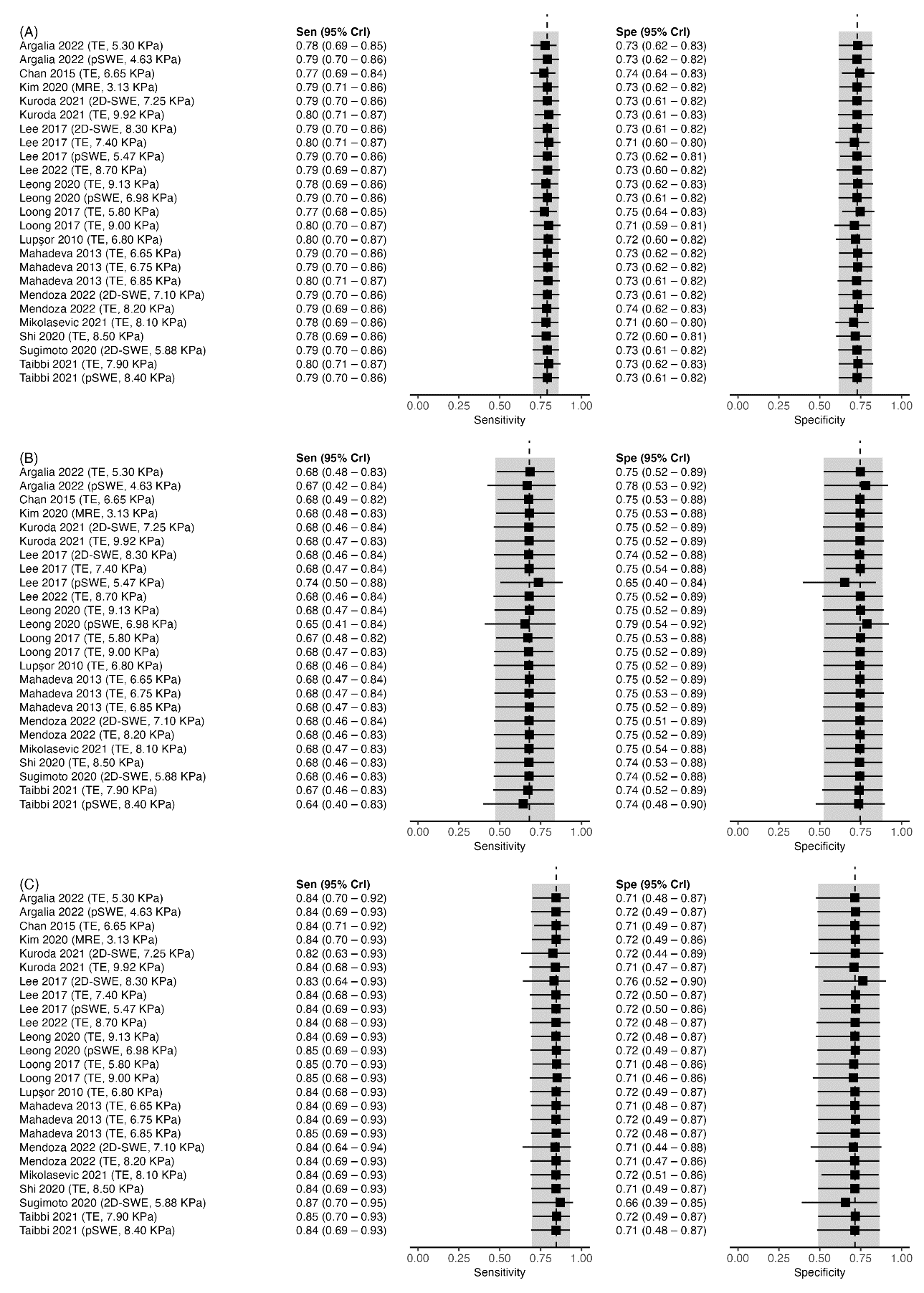


**Fig. S10: Forest plots of posterior estimates from the leave-one-out analysis for the main network meta-analysis (Fibrosis stage** ≥**2).** (A) TE (B) pSWE and (C) 2D-SWE. The left label indicates which study was excluded for each set of estimates, sorted by an ascending order of the first authors, published years, types of diagnosis, and cutoff thresholds. The dashed line is set at the posterior median of the main analysis. The grey band indicates the 95% credible interval of the main analysis.

Abbreviation: TE, transient elastography; pSWE, point shear wave elastography; 2D-SWE, two-dimensional shear wave elastography; and CrI, credible interval.


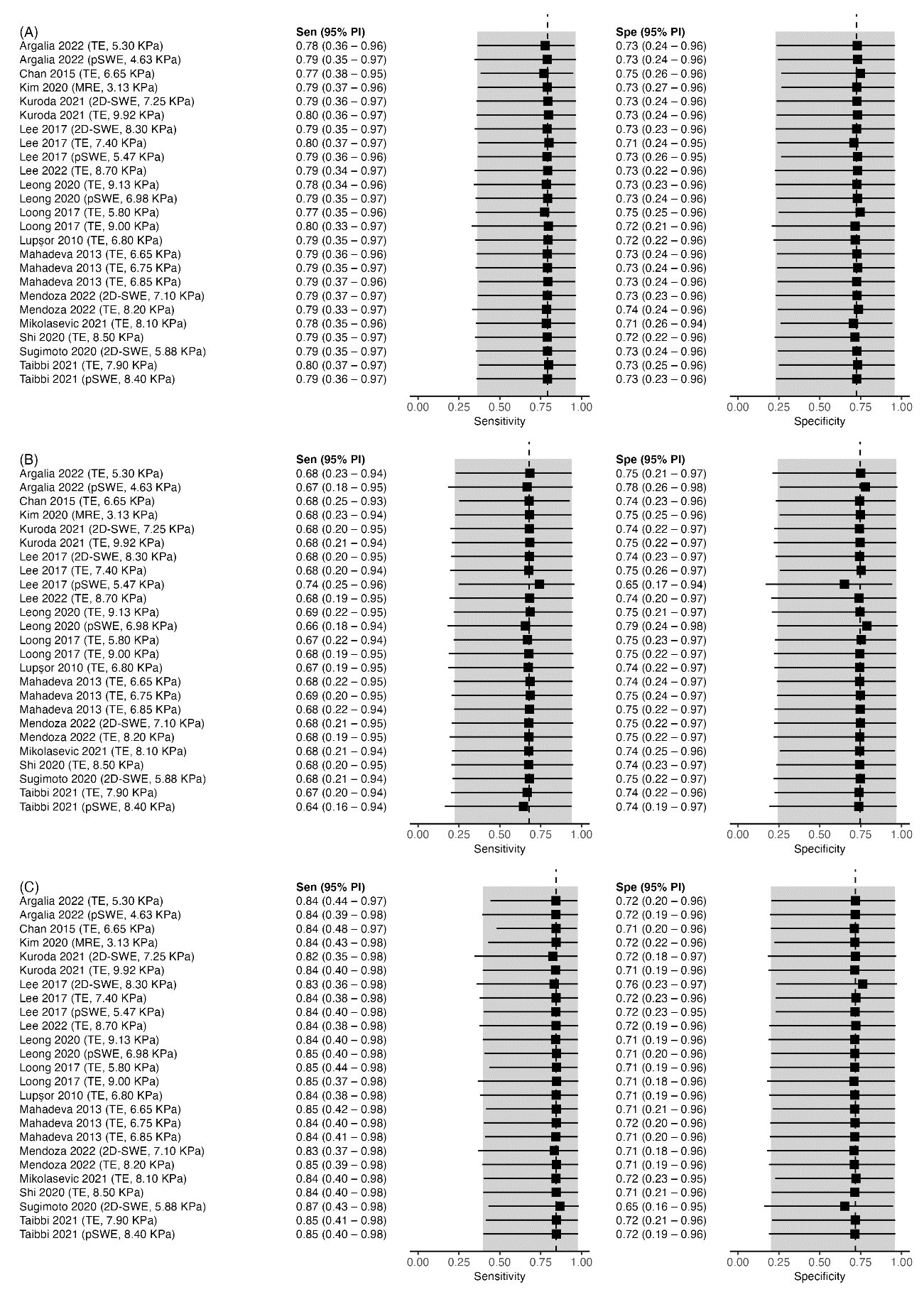


**Fig. S11: Forest plots of prediction estimates from the leave-one-out analysis for the main network meta-analysis (Fibrosis stage** ≥**2).** (A) TE (B) pSWE and (C) 2D-SWE. The left label indicates which study was excluded for each set of estimates, sorted by an ascending order of the first authors, published years, types of diagnosis, and cutoff thresholds. The dashed line is set at the posterior median of the main analysis. The grey band indicates the 95% prediction interval of the main analysis.

Abbreviation: TE, transient elastography; pSWE, point shear wave elastography; 2D-SWE, two-dimensional shear wave elastography; and PI, prediction interval.


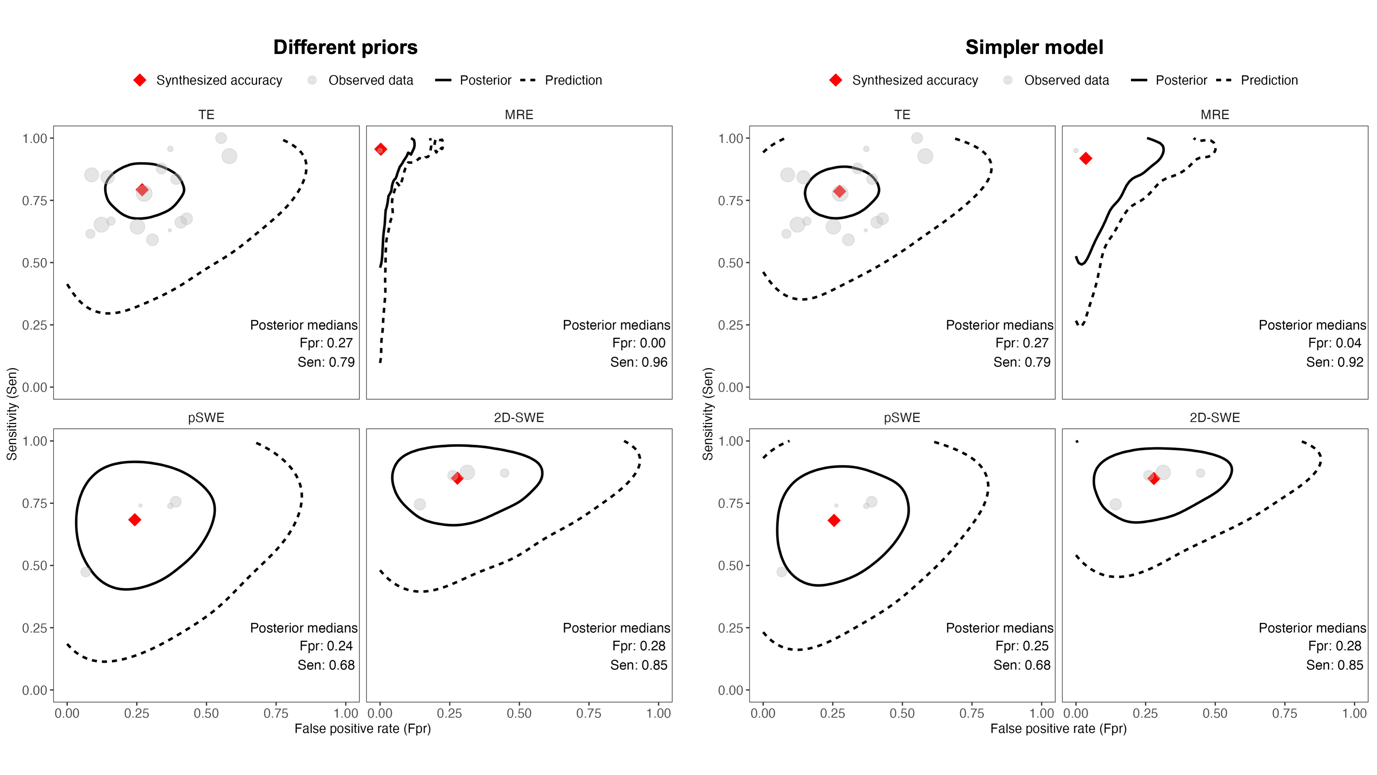


**Fig. S12: 95% credible and prediction regions from the sensitivity analysis of network meta-analysis for each echoic method (Fibrosis stage** ≥**2).** (Left) The different priors (Right) The simpler model. The posterior estimates represent medians of sensitivity and false positive rate. The solid black lines show 95% credible regions. The dashed lines show 95% prediction regions. The grey bubbles indicate observed data points with the bubble size proportional to the root of sample size of each study.

Abbreviation: TE, transient elastography; MRE, magnetic resonance elastography; pSWE, point shear wave elastography; 2D-SWE, two-dimensional shear wave elastography; Sen, sensitivity; and Fpr, false positive rate.

Fig. S13: Overlayed 95% credible and prediction regions of each echoic method from the sensitivity analysis of network meta-analysis (Fibrosis stage ≥2). (Top) The different priors (Bottom) The simpler model. (A) 95% credible regions and (B) 95% prediction regions. The solid line is TE. The dashed line is MRE. The dotted line is pSWE. The dot-dashed line is 2D-SWE.

Abbreviation: TE, transient elastography; MRE, magnetic resonance elastography; pSWE, point shear wave elastography; and 2D-SWE, two-dimensional shear wave elastography.

## Results for ≥F3


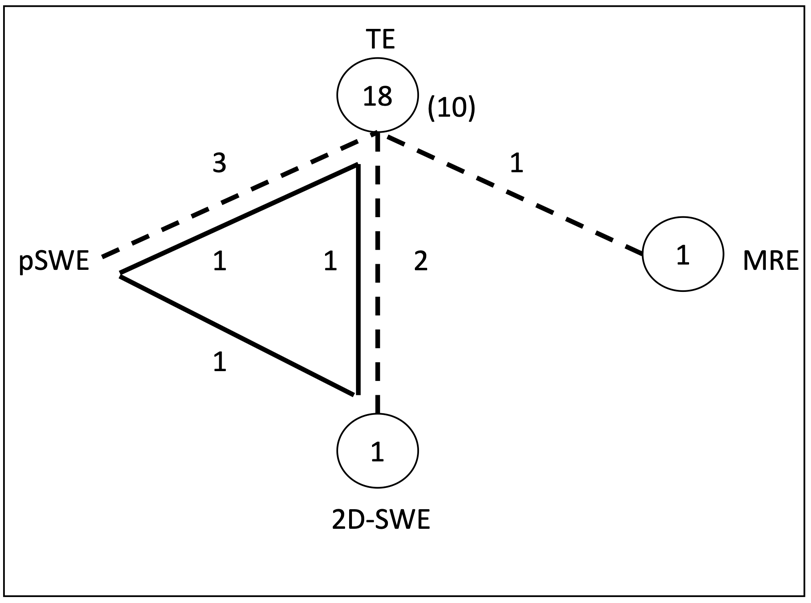


**Fig. S14: Network plots of the four elastographic methods. Nineteen studies with 36 observations for fibrosis stage** ≥**3.** Vertices represent different echoic methods. Diagnoses connected by lines indicate a comparison in 1 study. Dashed lines represent studies comparing 2 diagnoses in 1 study whereas solid lines represent studies comparing 3 diagnoses in 1 study. Numbers indicate the number of comparisons made between the 2 vertices connected by the line. The numbers in circles represent the number of observations as a single diagnosis.

Abbreviation: TE, transient elastography; pSWE, point shear wave elastography; 2D-SWE, two-dimensional shear wave elastography; and MRE, magnetic resonance elastography.


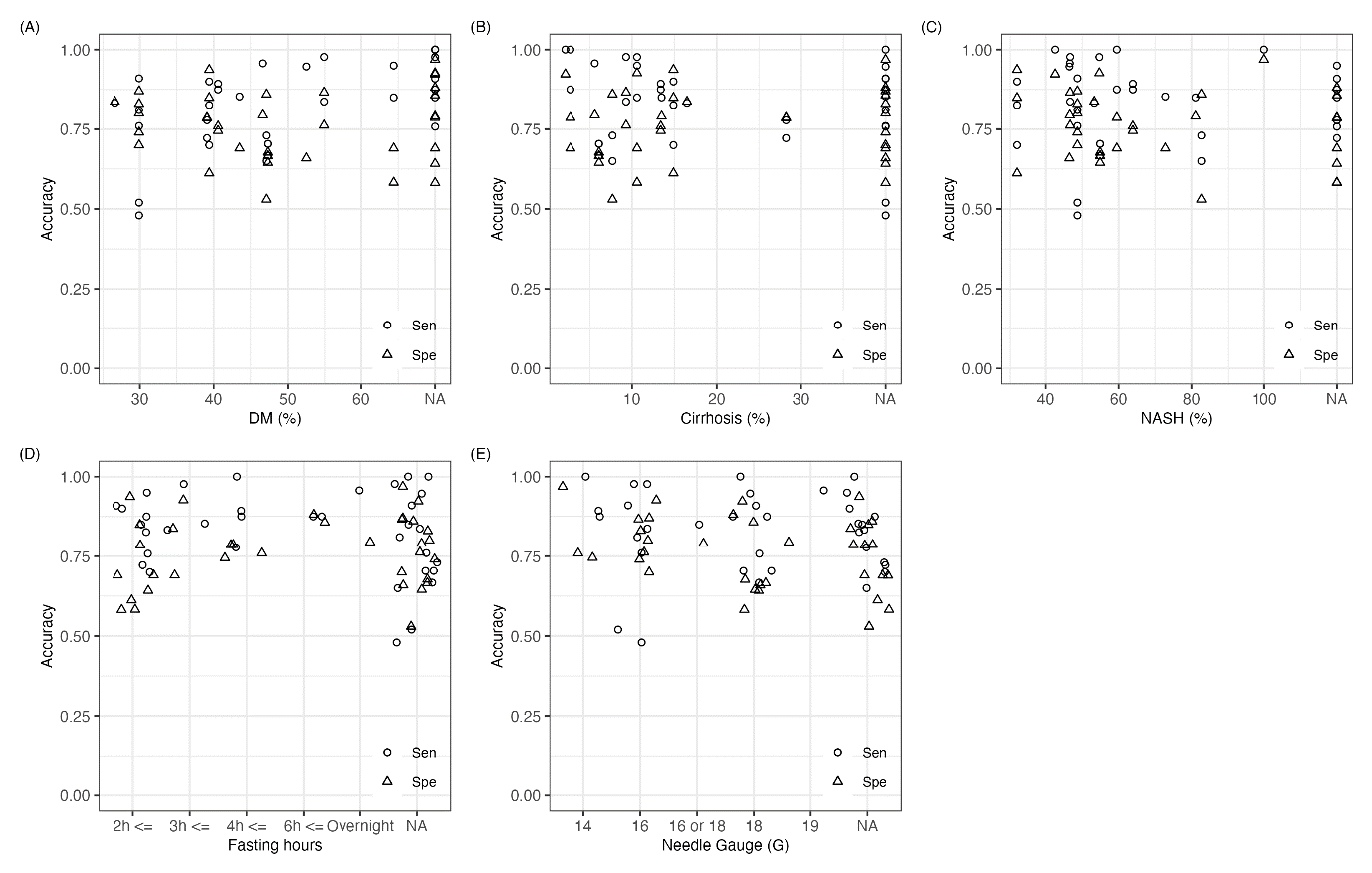


**Fig. S15:  Scattered plots of observed sensitivity and specificity of included studies over each characteristic (Fibrosis stage** ≥**3).**

Abbreviation: DM, diabetes mellitus; NASH, nonalcoholic steatohepatitis; h, hours; G, G needle gauge; Sen, sensitivity; Spe, specificity; and NA, not applicable.


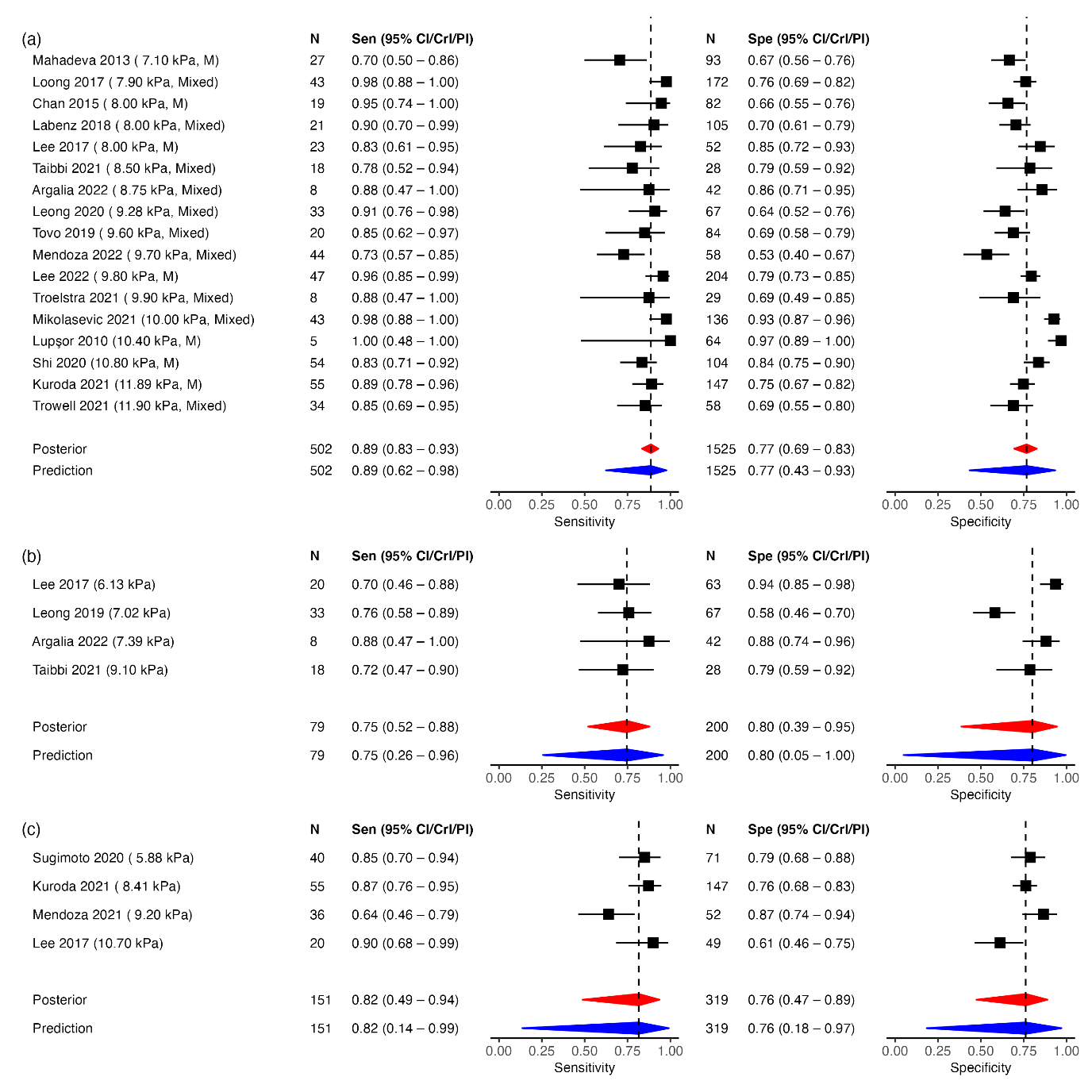


**Fig. S16: Forest plots of observed and pooled sensitivity and specificity values by separate meta-analysis (Fibrosis stage** ≥**3).** (A) TE (B) pSWE and (C) 2D-SWE. The left column represents first authors, published year (cutoff values, probe size [only for TE; medium (M) or mixed of different sizes (Mixed)]). The 95% confidence intervals for the include studies were re-calculated from the data using the exact binomial method. Red diamonds indicate 95% credible intervals and blue diamonds indicate 95% prediction intervals. The dashed line is set at a posterior median. In each method, the studies are sorted with lower cutoff values to those with higher.

Abbreviation: TE, transient elastography; pSWE, point shear wave elastography; 2D-SWE, two-dimensional shear wave elastography; Sen, sensitivity, Spe, specificity; ; CI, confidence interval; CrI, credible interval; and PI, prediction interval.


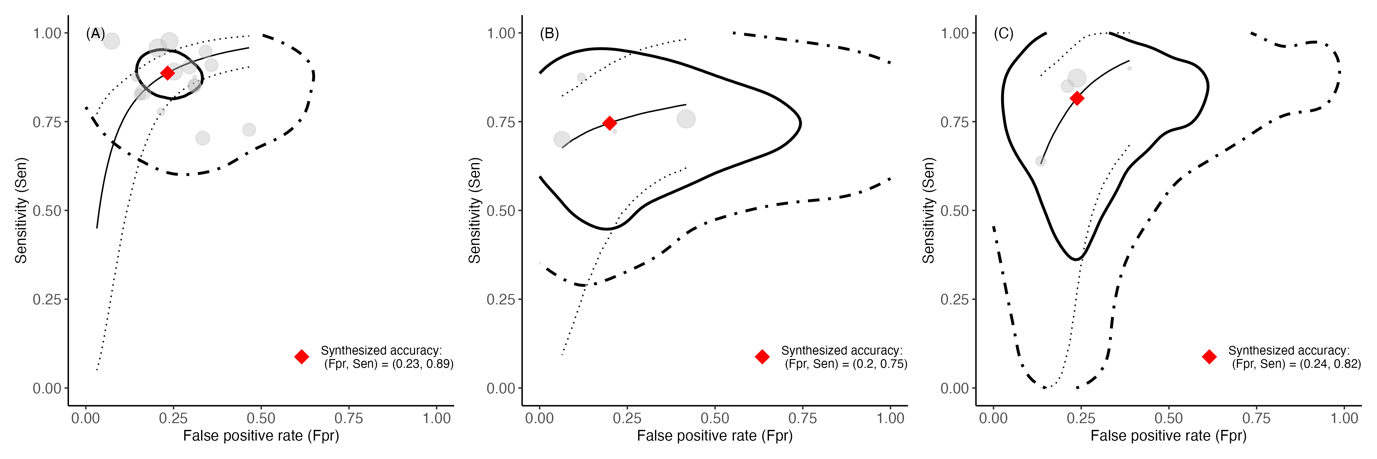


**Fig. S17: Hierarchical Summary Receiver Operator Characteristic (HSROC) curves with 95% credible and prediction intervals and regions from separate meta-analysis for each echoic method (Fibrosis stage** ≥**3).** (A) TE (B) pSWE and (C) 2D-SWE. The posterior estimates represent medians of sensitivity and false positive rate. The dotted gray bands show 95% credible intervals of the HSROC curve. The solid black lines show 95% credible regions. The dot-dashed lines show 95% prediction regions. The gray bubbles indicate observed data points with the bubble size proportional to the root of sample size of each study.

Abbreviation: TE, transient elastography; pSWE, point shear wave elastography; 2D-SWE, two-dimensional shear wave elastography; Sen, sensitivity, and Fpr, false positive rate.


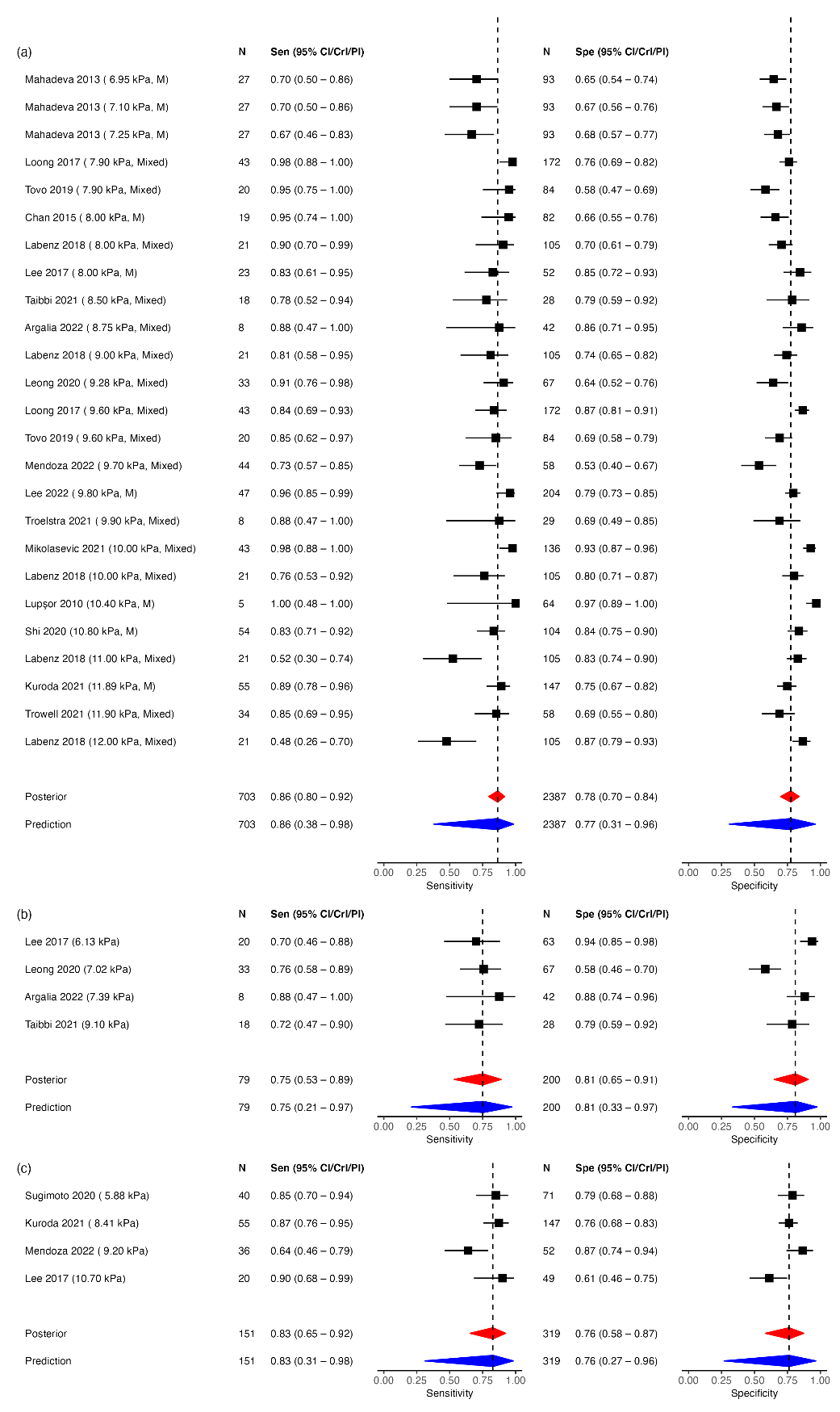


**Fig. S18: Forest plots of observed and pooled sensitivity and specificity values by the network meta-analysis (Fibrosis stage** ≥**3)**. (A) TE (B) pSWE and (C) 2D-SWE. The left column represents first authors, published year (cutoff values, probe size [only for TE; medium (M) or mixed of different sizes (Mixed)]). The 95% confidence intervals for the include studies were re-calculated from the data using the exact binomial method. Red diamonds indicate 95% credible intervals and blue diamonds indicate 95% predictive intervals. The dashed line is set at a posterior median. In each method, the studies are sorted with lower cutoff values to those with higher.

Abbreviation: TE, transient elastography; pSWE, point shear wave elastography; 2D-SWE, two-dimensional shear wave elastography; Sen, sensitivity, Spe, specificity; ; CI, confidence interval; CrI, credible interval; and PI, prediction interval.


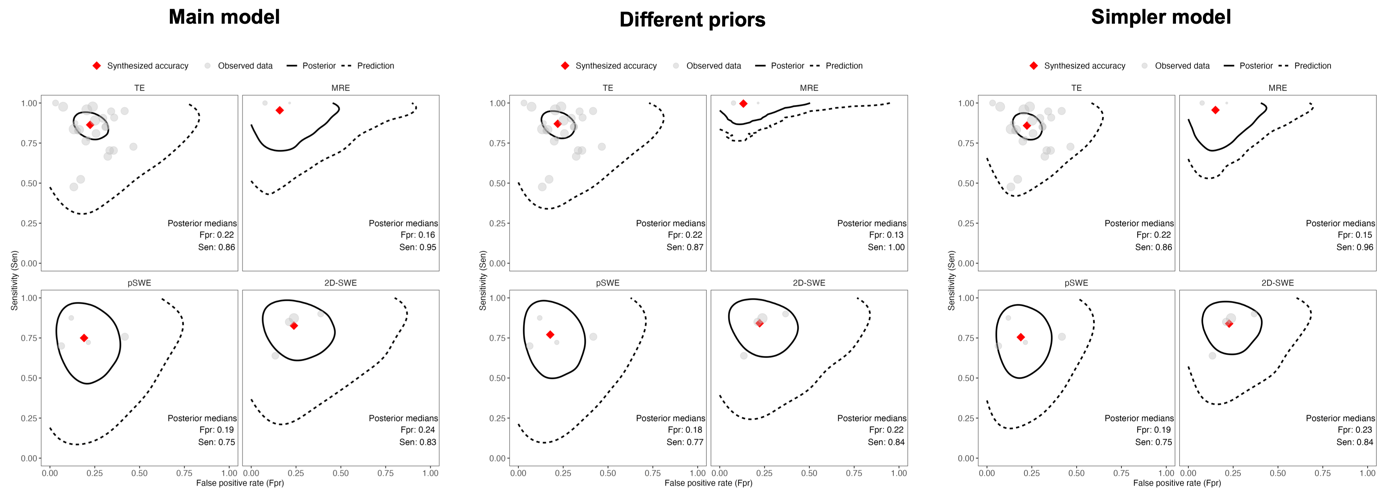


**Fig. S19: 95% credible and prediction regions from the sensitivity analysis of network meta-analysis for each echoic method (Fibrosis stage** ≥**3).** (Left) The main analysis (Middle) The different priors (Right) The simpler model. The posterior estimates represent the medians of sensitivity and false positive rate. The solid black lines show 95% credible regions. The dashed lines show 95% prediction regions. The gray bubbles indicate observed data points with the bubble size proportional to the root of sample size of each study.

Abbreviation: TE, transient elastography; MRE, magnetic resonance elastography; pSWE, point shear wave elastography; 2D-SWE, two-dimensional shear wave elastography; Sen, sensitivity, and Fpr, false positive rate.

**Fig. S20: Overlayed 95% credible and prediction regions of each echoic method from the sensitivity analysis of network meta-analysis (Fibrosis stage** ≥**3).** (Top) The main analysis (Middle) The different priors (Bottom) The simpler model. (A) 95% credible regions and (B) 95% prediction regions. The solid line is TE. The dashed line is MRE. The dotted line is pSWE. The dot-dashed line is 2D-SWE.

Abbreviation: TE, transient elastography; MRE, magnetic resonance elastography; pSWE, point shear wave elastography; 2D-SWE, two-dimensional shear wave elastography; Sen, sensitivity, and Fpr, false positive rate.

**
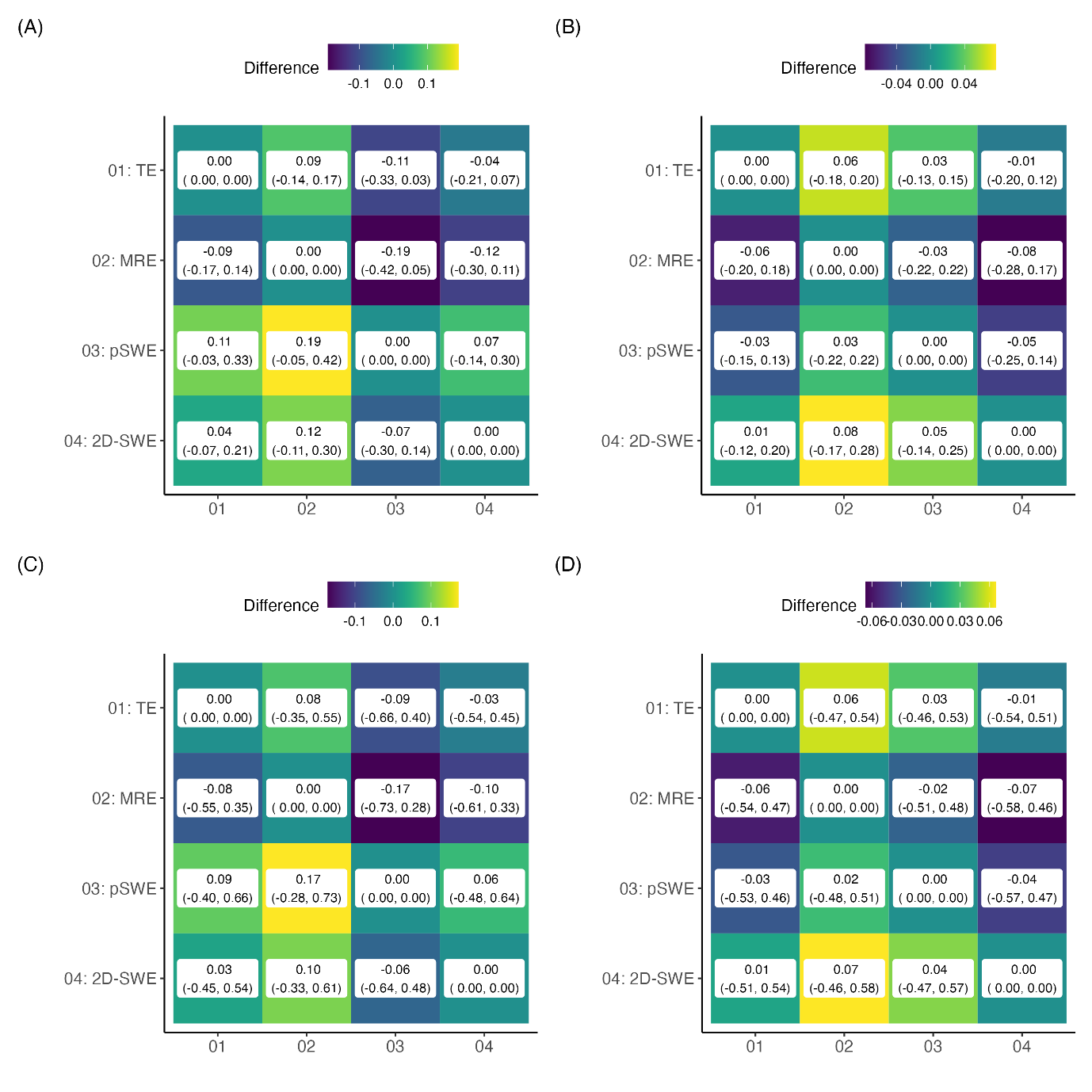
**

**Fig. S21: League tables of the pairwise differences in sensitivity and specificity in posterior and predictive distributions obtained from the main network meta-analysis model (Fibrosis stage** ≥**3).** Each cell reads the column method minus row method. (A) Posterior, sensitivity (B) Posterior, specificity (C) Prediction, sensitivity and (D) Prediction, specificity.

Abbreviation: TE, transient elastography; MRE, magnetic resonance elastography; pSWE, point shear wave elastography; and 2D-SWE, two-dimensional shear wave elastography.


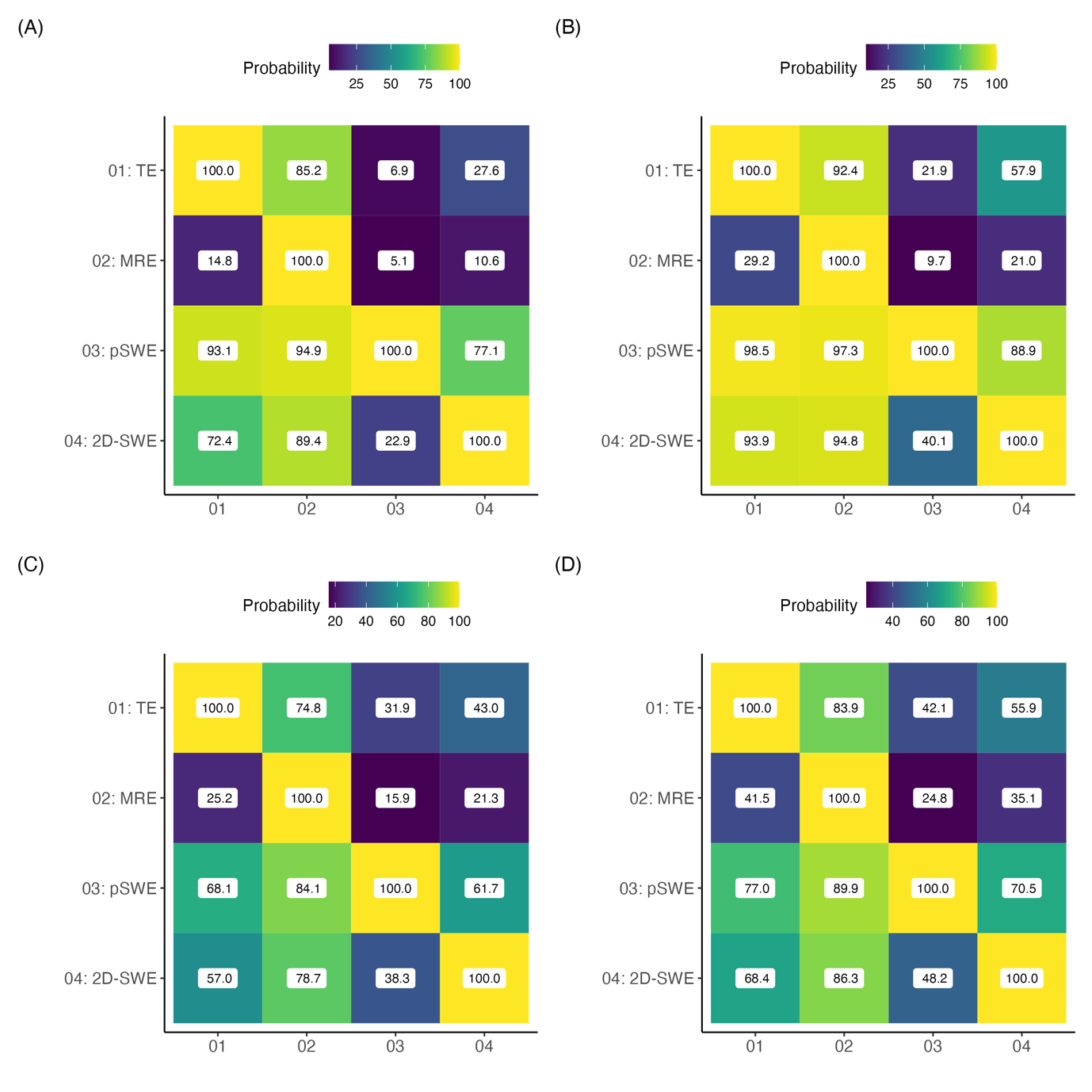


**Fig. S22: League tables of probabilities (%) being the pairwise differences are equal to or greater than a margin in sensitivity in posterior and predictive distributions obtained from the main network meta-analysis model (Fibrosis stage** ≥**3).** Each cell reads the column method minus row method. (A) Posterior, margin = 0% (B) Posterior, margin = -5% (C) Prediction, margin = 0% and (D) Prediction, margin = -5%.

Abbreviation: TE, transient elastography; MRE, magnetic resonance elastography; pSWE, point shear wave elastography; and 2D-SWE, two-dimensional shear wave elastography.


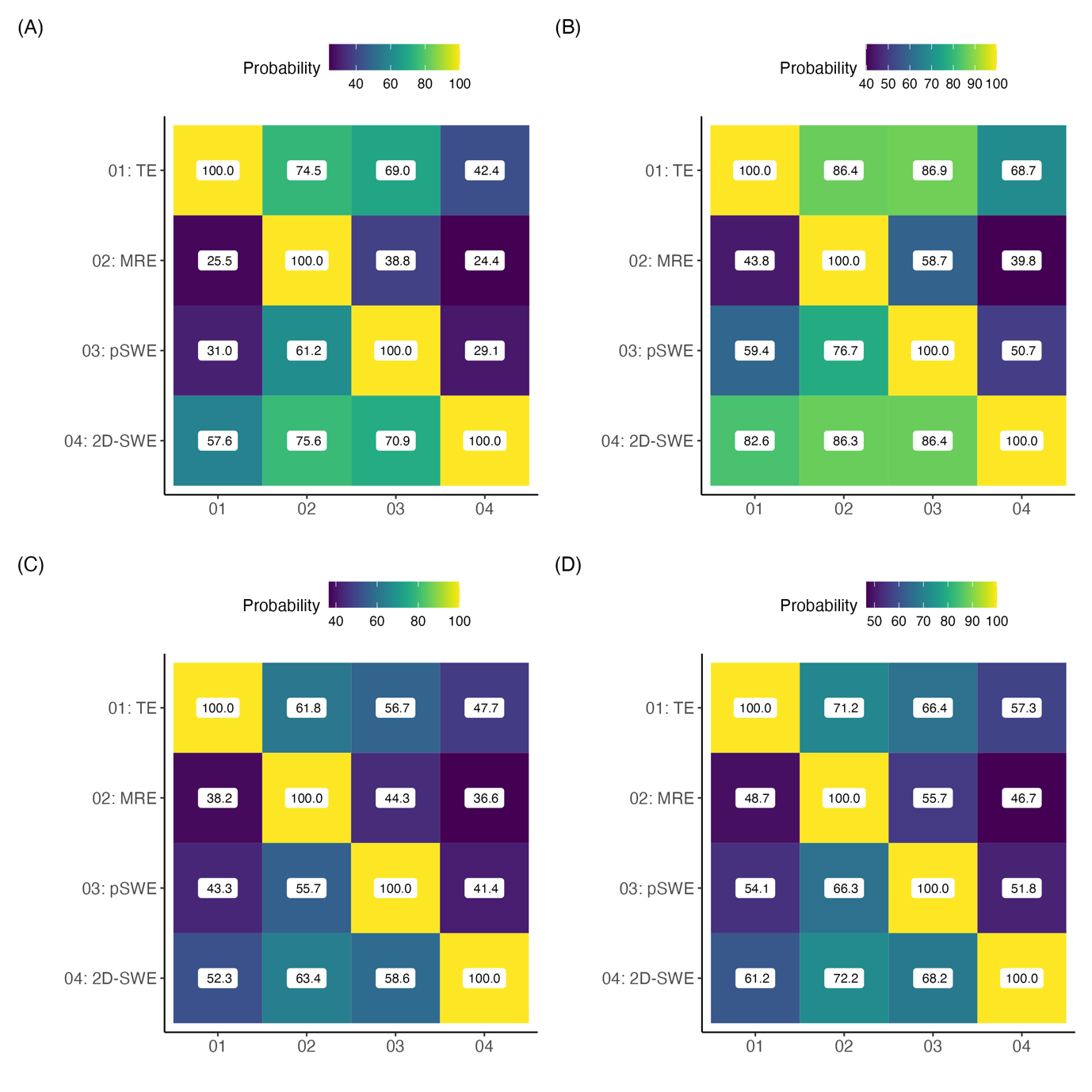


**Fig. S23: League tables of probabilities (%) being the pairwise differences are equal to or greater than a margin in specificity in posterior and predictive distributions obtained from the main network meta-analysis model (Fibrosis stage** ≥**3).** Each cell reads the column method minus row method. (A) Posterior, margin = 0% (B) Posterior, margin = -5% (C) Prediction, margin = 0% and (D) Prediction, margin = -5%.

Abbreviation: TE, transient elastography; MRE, magnetic resonance elastography; pSWE, point shear wave elastography; and 2D-SWE, two-dimensional shear wave elastography.


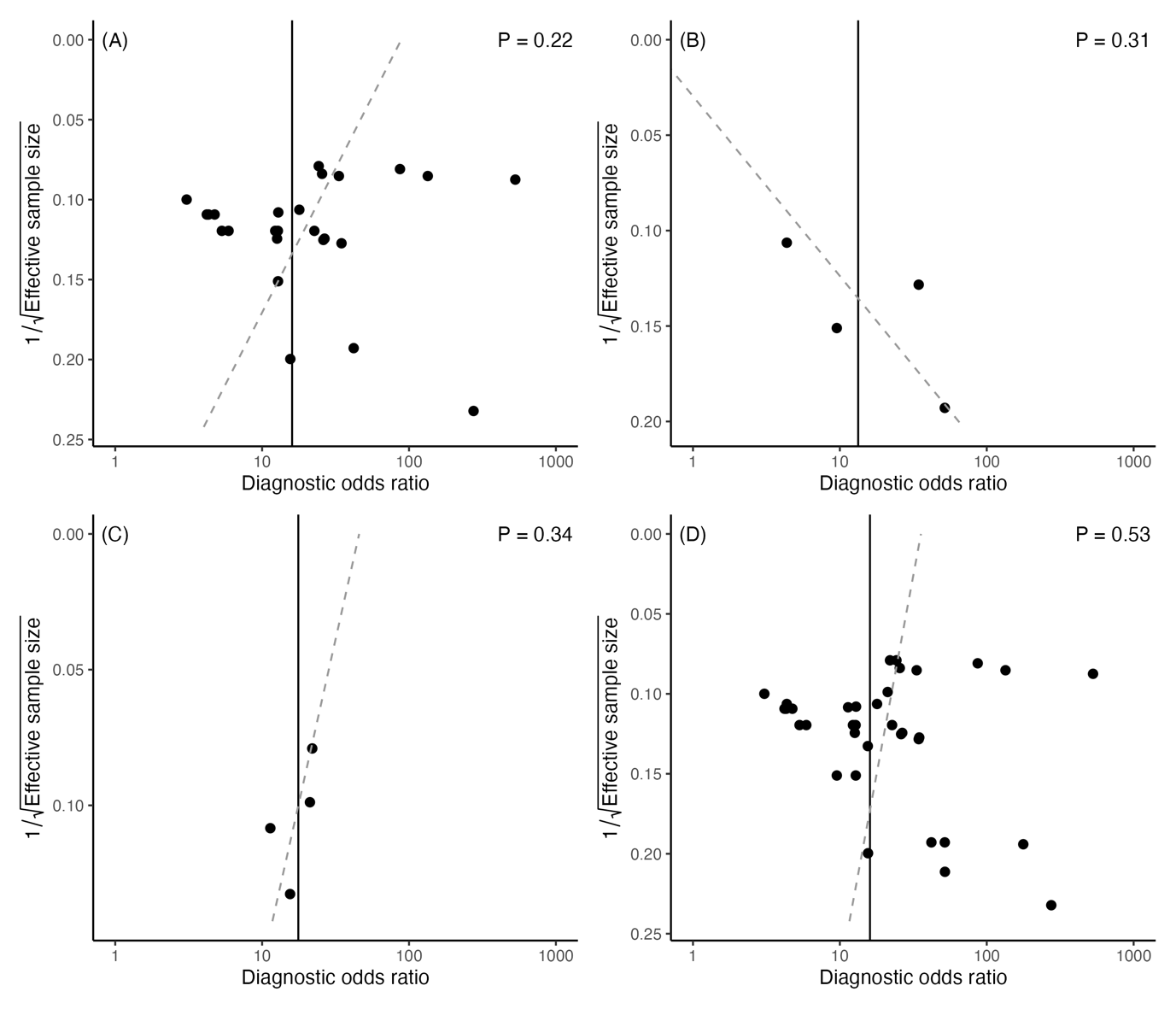


**Fig. S24: Deeks’ funnel plots with P-values from their asymmetry tests (Fibrosis stage** ≥**3).** (A) TE (B) pSWE (C) 2D-SWE and (D) All. The dot represents each observation. The vertical solid line represents the pooled means of a random-effects model. The dashed grey line represents the weighted regression line of log (diagnostic odds ratio) on the inverse of root of effective sample size (ESS) with ESS as weights. Continuity correction was used for all cell counts used. Two-sided P-value, which is calculated using the slope divided by its standard error following a t distribution with number of studies − 2 degrees of freedom, is noted at the top right in each graph.

Abbreviation: TE, transient elastography; pSWE, point shear wave elastography; and 2D-SWE, two-dimensional shear wave elastography.

## Results for ≥F1


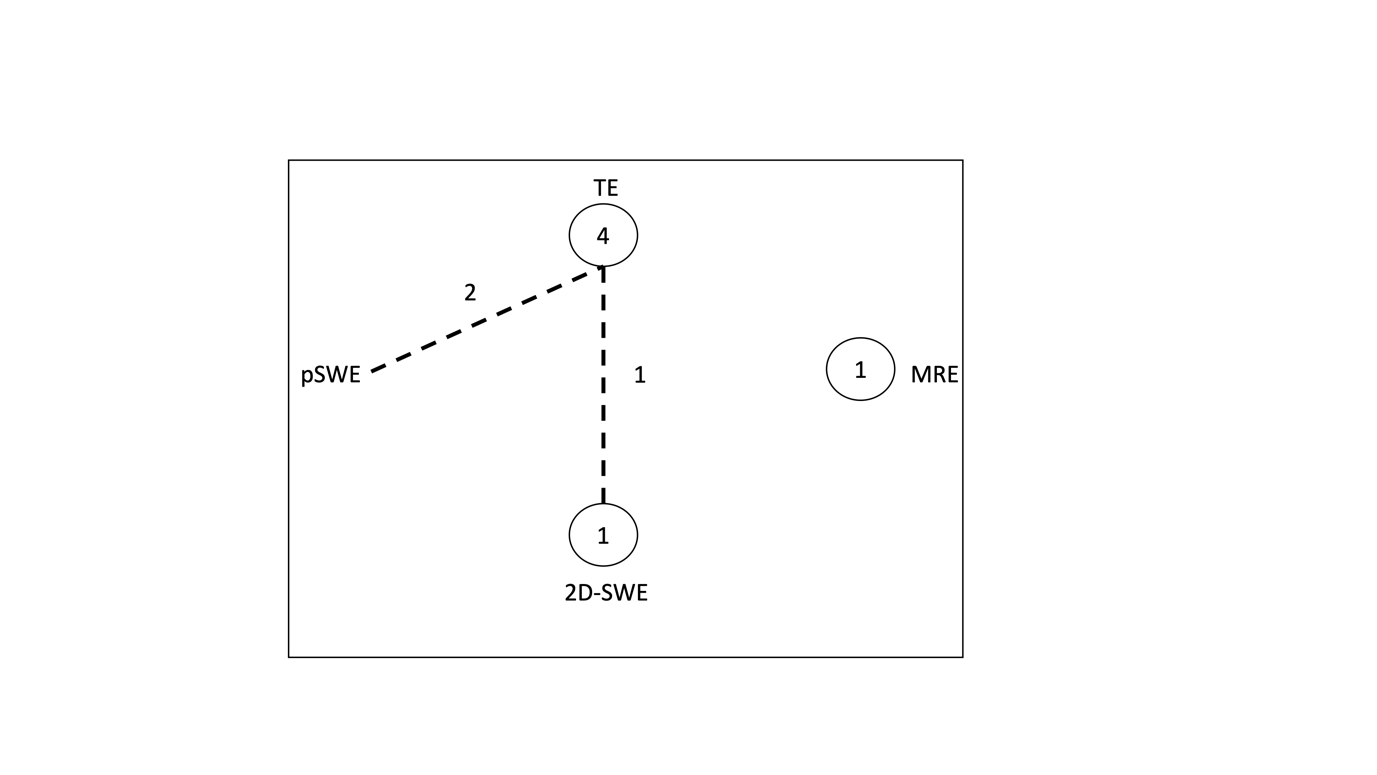


**Fig. S25: Network plots of the four elastographic methods. Nine studies with 12 observations for fibrosis stage** ≥**1.** Vertices represent different echoic methods. Diagnoses connected by lines indicate a comparison in 1 study. Dashed lines represent studies comparing 2 diagnoses in 1 study whereas solid lines represent studies comparing three diagnoses in 1 study. Numbers indicate the number of comparisons made between the 2 vertices connected by the line. The numbers in circles represent the number of observations as a single diagnosis.

Abbreviation: TE, transient elastography; pSWE, point shear wave elastography; 2D-SWE, two-dimensional shear wave elastography; and MRE, magnetic resonance elastography.


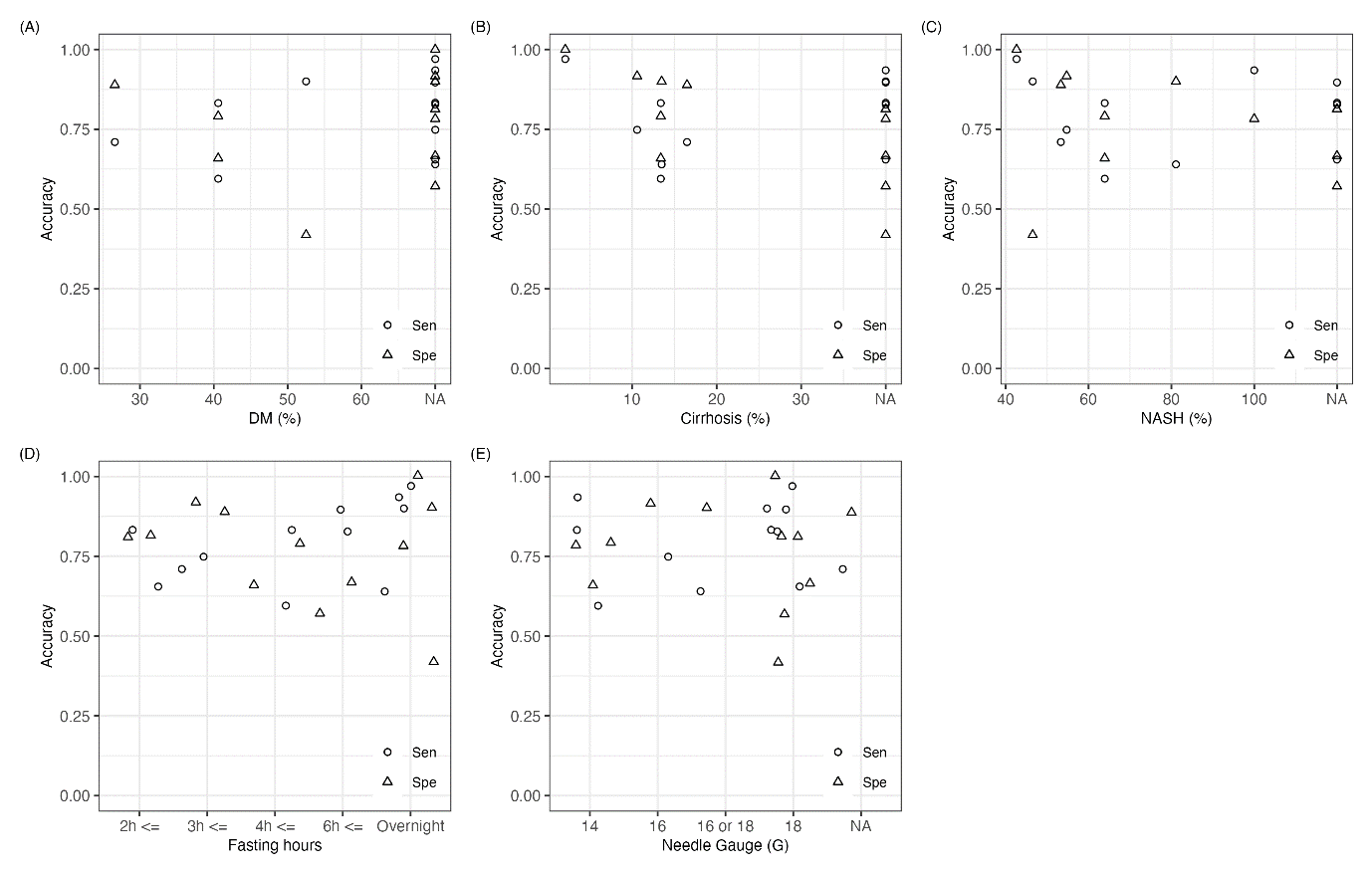


**Fig. S26:  Scattered plots of observed sensitivity and specificity of included studies over each characteristic (Fibrosis stage** ≥**1).**

Abbreviation: DM, diabetes mellitus; NASH, nonalcoholic steatohepatitis; h, hours; G, G needle gauge; Sen, sensitivity; Spe, specificity; and NA, not applicable.


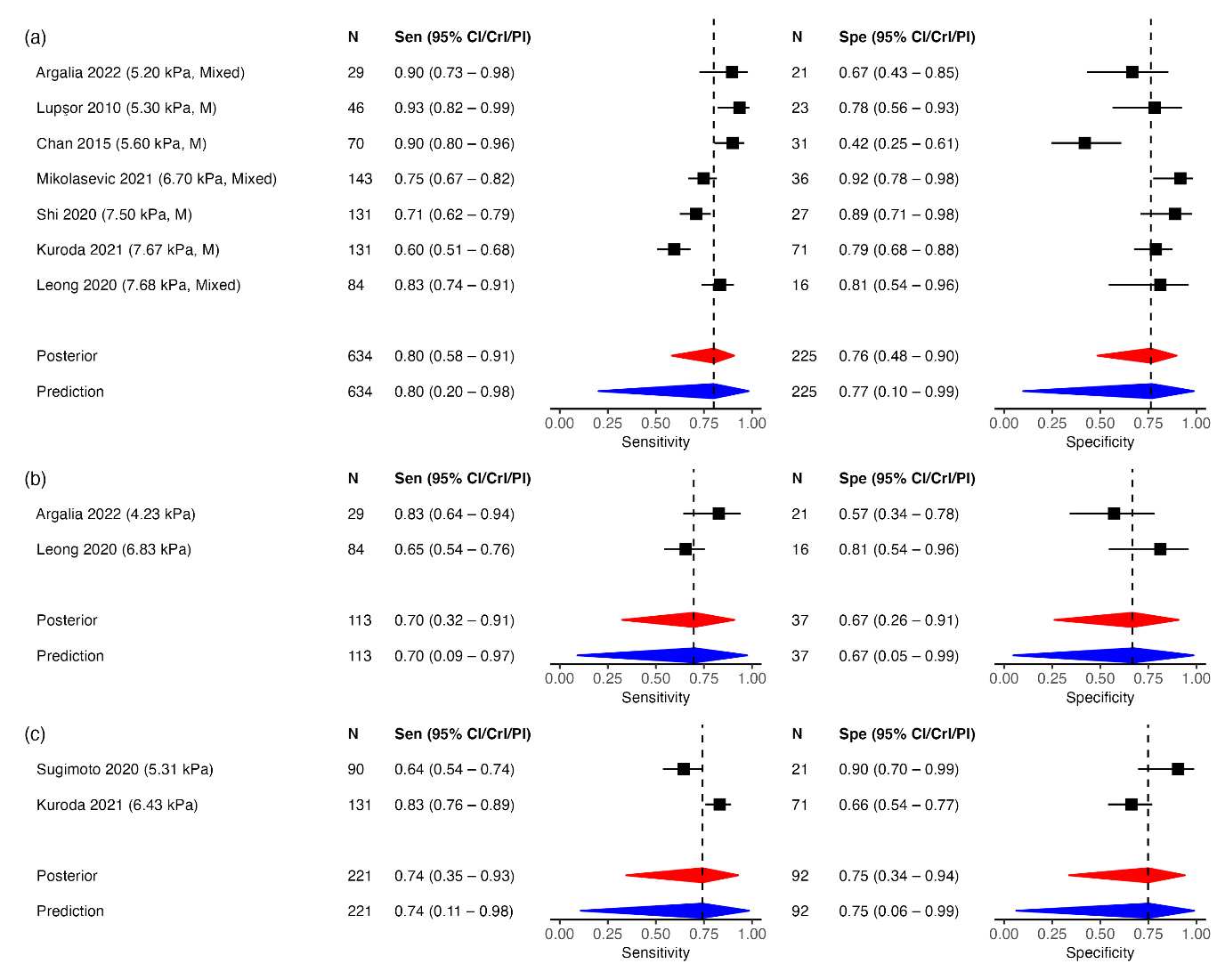


**Fig. S27: Forest plots of observed and pooled sensitivity and specificity values by the network meta-analysis using the simpler model (Fibrosis stage** ≥**1)**. (A) TE (B) pSWE and (C) 2D-SWE. The left column represents first authors, published year (cutoff values, probe size [only for TE; medium (M) or mixed of different sizes (Mixed)]). The 95% confidence intervals for the include studies were re-calculated from the data using the exact binomial method. Red diamonds indicate 95% credible intervals and blue diamonds indicate 95% predictive intervals. The dashed line is set at a posterior median. In each method, the studies are sorted with lower cutoff values to those with higher.

Abbreviation: TE, transient elastography; pSWE, point shear wave elastography; 2D-SWE, two-dimensional shear wave elastography; Sen, sensitivity, Spe, specificity; CI, confidence interval; CrI, credible interval; and PI, prediction interval.


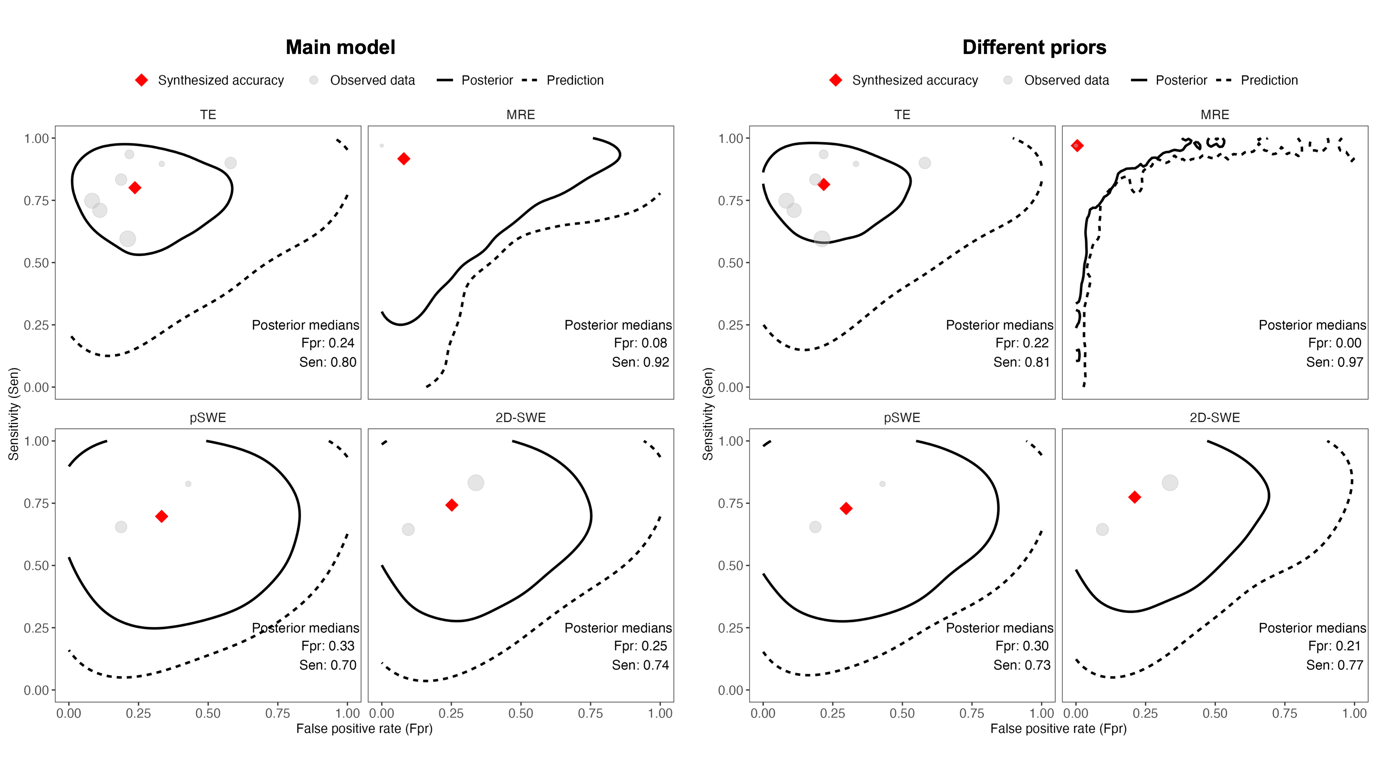
**Fig. S28: 95% credible and prediction regions from the network meta-analysis for each echoic method (Fibrosis stage** ≥**1).** (Left) The main analysis [simpler model] (Right) The different priors. The posterior estimates represent the medians of sensitivity and false positive rate. The solid black lines show 95% credible regions. The dashed lines show 95% prediction regions. The grey bubbles indicate observed data points with the bubble size proportional to the root of sample size of each study.

Abbreviation: TE, transient elastography; MRE, magnetic resonance elastography; pSWE, point shear wave elastography; 2D-SWE, two-dimensional shear wave elastography; Sen, sensitivity, and Fpr, false positive rate.

**Fig. S29: Overlayed 95% credible and prediction regions of each echoic method from the network meta-analysis (Fibrosis stage** ≥**1).** (Top) The main analysis [simpler model] (Bottom) The different priors. (A) 95% Credible regions and (B) 95% Prediction regions. The solid line is TE. The dashed line is MRE. The dotted line is pSWE. The dot-dashed line is 2D-SWE.

Abbreviation: TE, transient elastography; MRE, magnetic resonance elastography; pSWE, point shear wave elastography; 2D-SWE, two-dimensional shear wave elastography; Sen, sensitivity, and Fpr, false positive rate.


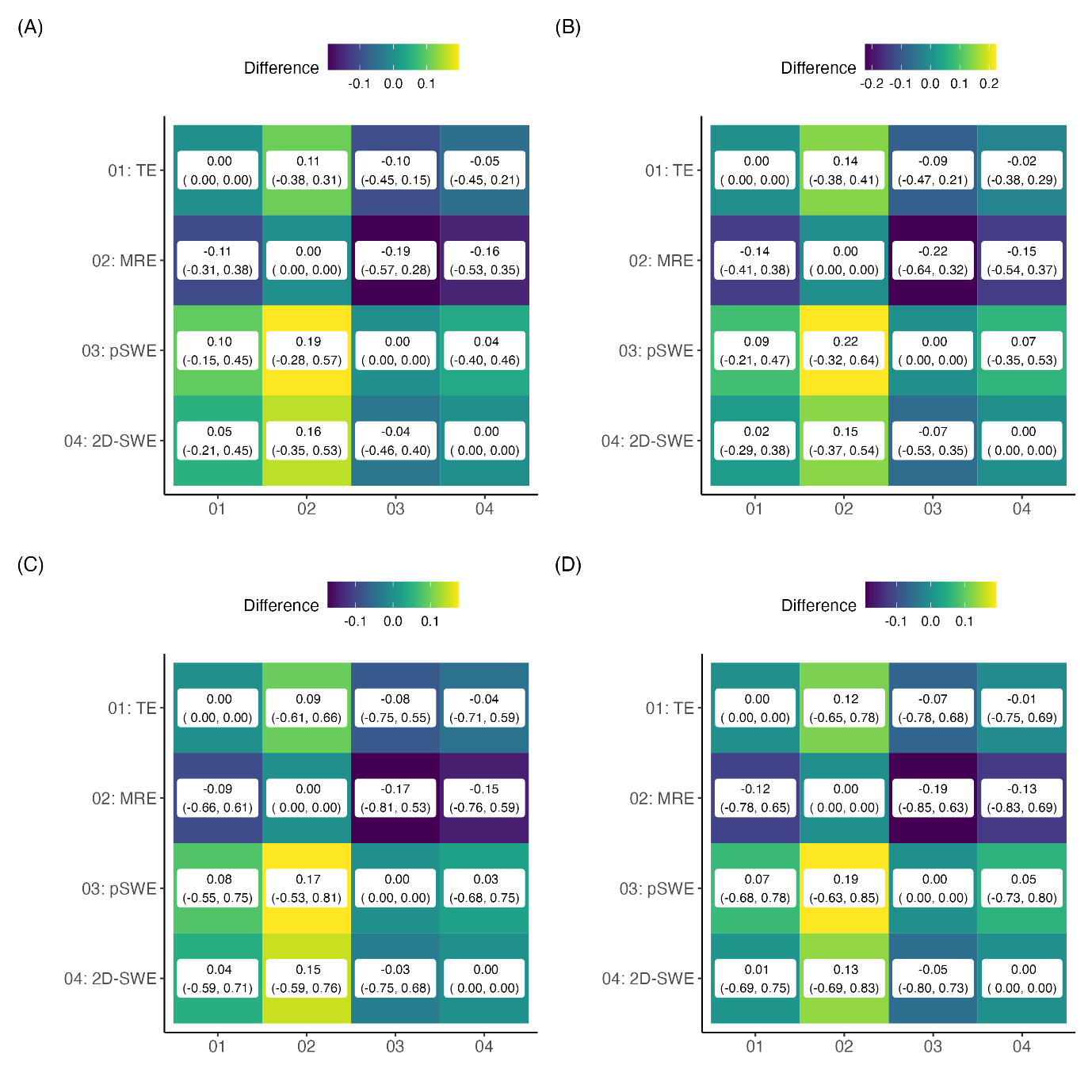


**Fig. S30: League tables of the pairwise differences in sensitivity and specificity in posterior and predictive distributions obtained from the main (simpler) network meta-analysis model (Fibrosis stage** ≥**1):** Each cell reads the column method minus row method. (A) Posterior, sensitivity (B) Posterior, specificity (C) Prediction, sensitivity and (D) Prediction, specificity.

Abbreviation: TE, transient elastography; MRE, magnetic resonance elastography; pSWE, point shear wave elastography; and 2D-SWE, two-dimensional shear wave elastography.


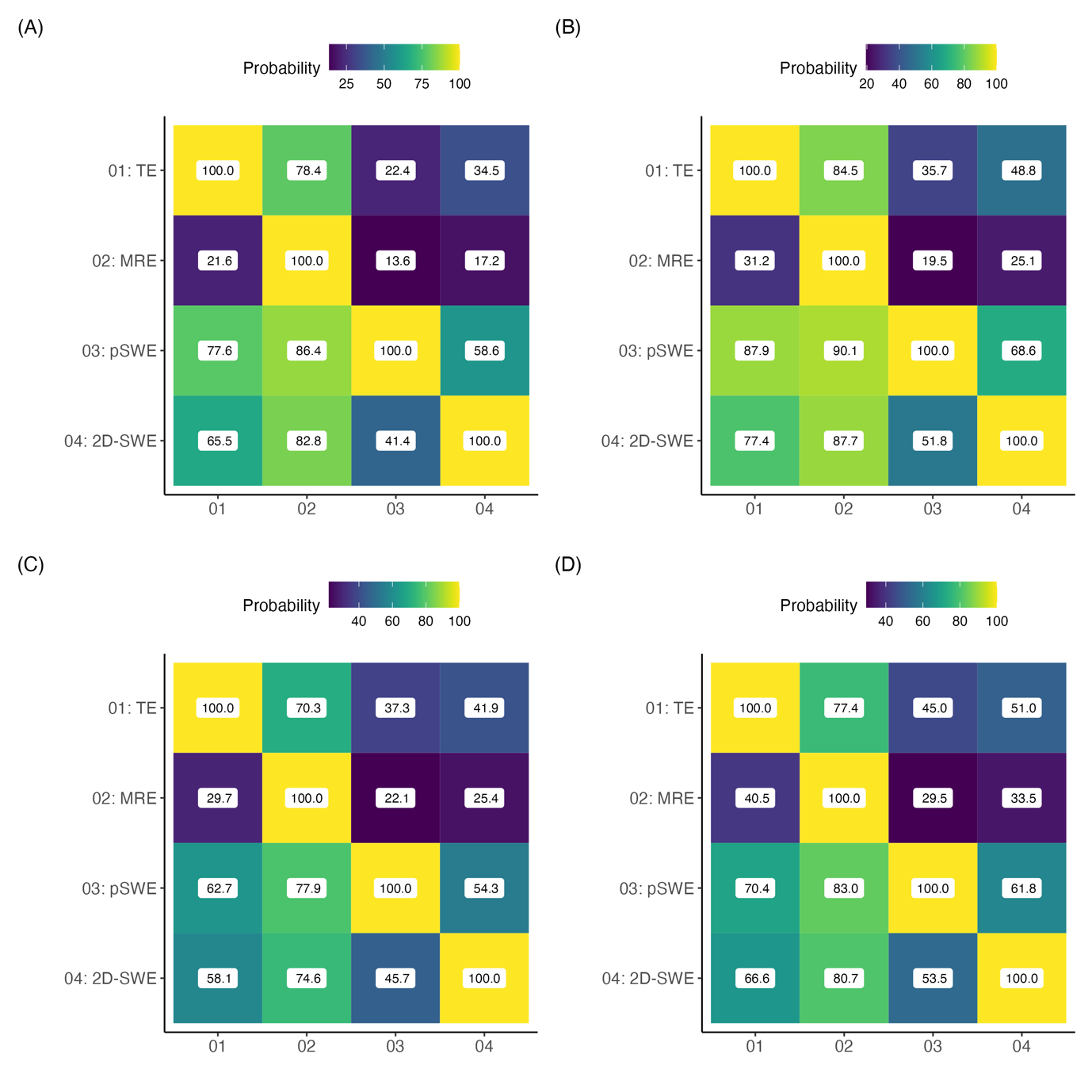


**Fig. S31: League tables of probabilities (%) being the pairwise differences are equal to or greater than a margin in sensitivity in posterior and predictive distributions obtained from the main (simpler) network meta-analysis model (Fibrosis stage** ≥**1):** Each cell reads the column method minus row method. (A) Posterior, margin = 0% (B) Posterior, margin = -5% (C) Prediction, margin = 0% and (D) Prediction, margin = -5%.

Abbreviation: TE, transient elastography; MRE, magnetic resonance elastography; pSWE, point shear wave elastography; and 2D-SWE, two-dimensional shear wave elastography.


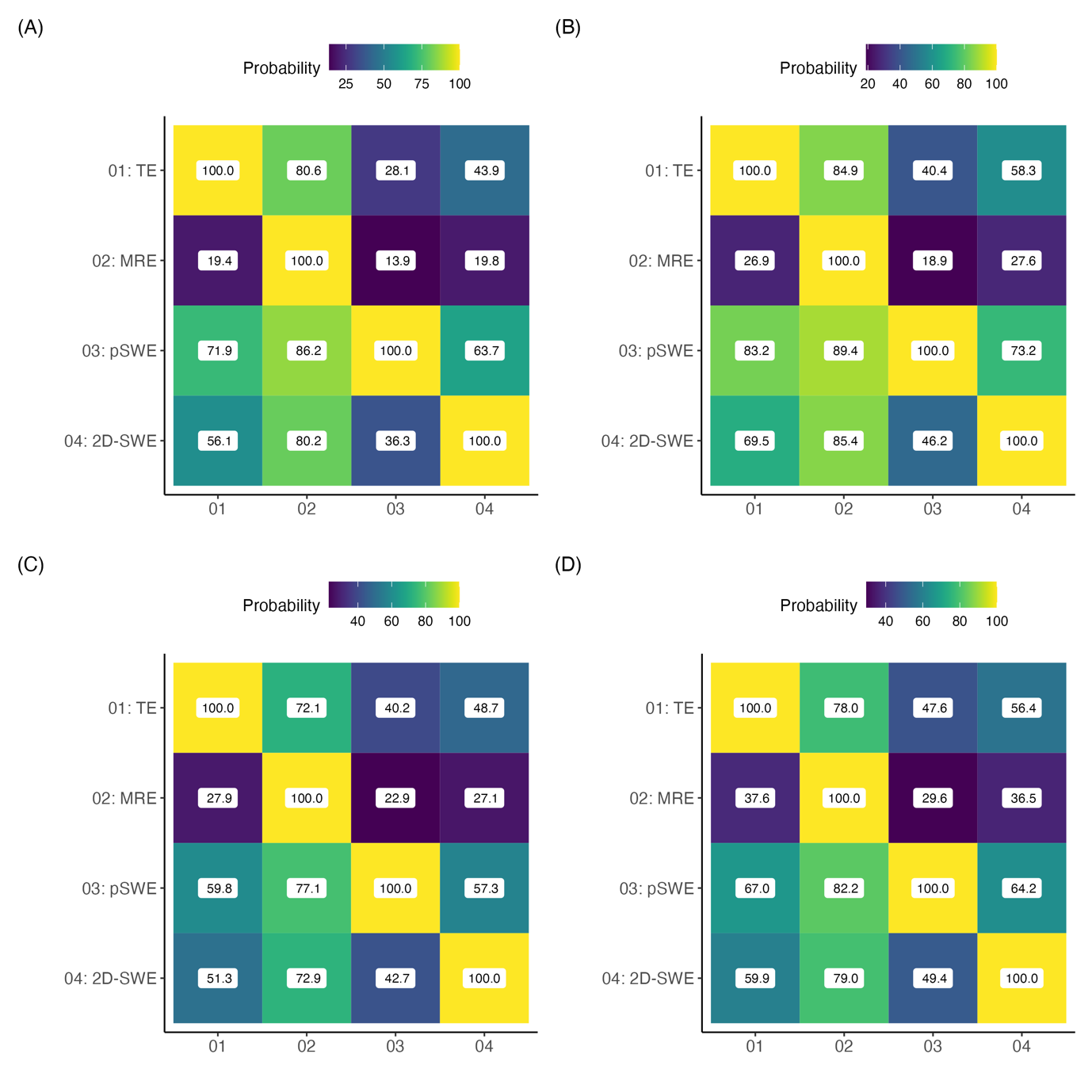


**Fig. S32: League tables of probabilities (%) being the pairwise differences are equal to or greater than a margin in specificity in posterior and predictive distributions obtained from the main (simpler) network meta-analysis model (Fibrosis stage** ≥**1):** Each cell reads the column method minus row method. (A) Posterior, margin = 0% (B) Posterior, margin = -5% (C) Prediction, margin = 0% and (D) Prediction, margin = -5%.

Abbreviation: TE, transient elastography; MRE, magnetic resonance elastography; pSWE, point shear wave elastography; and 2D-SWE, two-dimensional shear wave elastography.


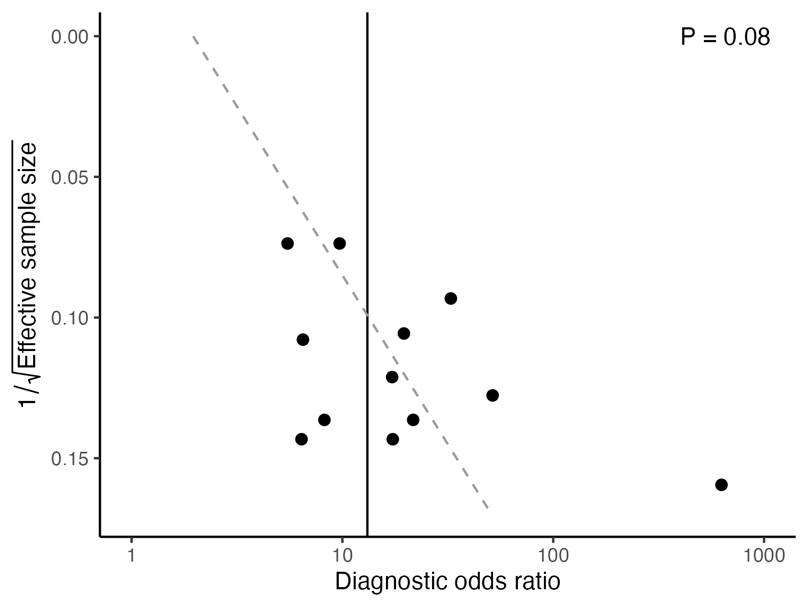


**Fig. S33: Deeks’ funnel plots with P-values from their asymmetry tests (Fibrosis stage** ≥**1).** The dot represents each observation. The vertical solid line represents the pooled means of a random-effects model. The dashed grey line represents the weighted regression line of log (diagnostic odds ratio) on the inverse of root of effective sample size (ESS) with ESS as weights. Continuity correction was used for all cell counts used. Two-sided P-value, which is calculated using the slope divided by its standard error following a t distribution with number of studies - 2 degrees of freedom, is noted at the top right in the graph.
