# Supplementary material for: Comparison of the diagnostic accuracy of shear wave elastography with transient elastography in adult nonalcoholic fatty liver disease: a systematic review and network meta-analysis of diagnostic test accuracy
Source: Abdom Radiol (NY). 2024 Sep 6;50(2):734–46. doi: 10.1007/s00261-024-04546-8 (PMC11794403; doi:10.1007/s00261-024-04546-8)
Supplement: Supplementary file 3 — Supplementary file3 (PDF 6698 KB) [file 261_2024_4546_MOESM3_ESM.pdf]

# Fitting and convergence diagnostics of NMA main analysis

```r
fibrosis_grade = "F2"
prior_type = 1 # main prior
```

```r
library(tidyverse)
library(here)
library(rstan)
source(here("scripts","98_for_source","create_datasets_supp.R"))

# -----------------------------------------------
# Fitting
# -----------------------------------------------

# Read data
d <- readxl::read_excel(here("data", "NMA_data_supp.xlsx"),sheet = 1)

d2<-
  d %>%
  # Include studies whose cutoffs are not defined to get 90 % of sen or spe
  filter(Flag_Threshold_sesp_90 == 0)

df <- create_dataset_NMA(d2, fibrosis_grade)

# data for stan
data_stan <- list(N = nrow(df),
                  J = 2,
                  S = length(unique(df$`Rayyan ID`)),
                  T = length(unique(df$Test)),
                  Test = df$Test_Num,
                  Study = df$Study_Num,
                  Threshold = df$Threshold_Num,
                  TP = df$TP,
                  FP = df$FP,
                  FN = df$FN,
                  TN = df$TN,
                  prior_type = prior_type
)


model <- stan_model(file = here("scripts","4_3_NMA",
                                "nma_fit_main_supp.stan"))
```

```
## Running /Library/Frameworks/R.framework/Resources/bin/R CMD SHLIB foo.c
## clang -mmacosx-version-min=10.13 -I"/Library/Frameworks/R.framework/Resources/include" -DNDEBUG   -I
## In file included from <built-in>:1:
## In file included from /Users/tetsuroda/Library/Caches/org.R-project.R/R/renv/cache/v5/R-4.1/x86_64-ap
```

```
## In file included from /Users/tetsuroda/Dropbox/NMA_Liver_Diagnosis/nma_nash/renv/library/R-4.1/x86_64
## In file included from /Users/tetsuroda/Dropbox/NMA_Liver_Diagnosis/nma_nash/renv/library/R-4.1/x86_64
## /Users/tetsuroda/Dropbox/NMA_Liver_Diagnosis/nma_nash/renv/library/R-4.1/x86_64-apple-darwin17.0/Rcpp
## namespace Eigen {
## ^
## /Users/tetsuroda/Dropbox/NMA_Liver_Diagnosis/nma_nash/renv/library/R-4.1/x86_64-apple-darwin17.0/Rcpp
## namespace Eigen {
##                ^
##                ;
## In file included from <built-in>:1:
## In file included from /Users/tetsuroda/Library/Caches/org.R-project.R/R/renv/cache/v5/R-4.1/x86_64-ap
## In file included from /Users/tetsuroda/Dropbox/NMA_Liver_Diagnosis/nma_nash/renv/library/R-4.1/x86_64
## /Users/tetsuroda/Dropbox/NMA_Liver_Diagnosis/nma_nash/renv/library/R-4.1/x86_64-apple-darwin17.0/Rcpp
## #include <complex>
##          ^~~~~~~~~
## 3 errors generated.
## make: *** [foo.o] Error 1
```

```
options(mc.cores = parallelly::availableCores()-1)
rstan_options("auto_write" = TRUE)
rstan_options(javascript = FALSE)

iter_val <- 30000
warmup_val <- 5000
chains_val <- 4
thin_val <- 10

# mcmc
fit <- sampling(
  model,
  data = data_stan,
  seed = 9999,
  iter = iter_val,
  warmup = warmup_val,
  chains = chains_val,
  thin = thin_val,
  control = list(adapt_delta = 0.99,
                 max_treedepth = 12)
)

# store large outputs
parms <- c("muSe","muSp","sigSe","sigSp","tau0_se",
           "tau1_se","tau0_sp","tau1_sp")

ms <- rstan::extract(fit)
s_params <- summary(fit, pars = parms)
s_p <- summary(fit, pars = c("sepool","sppool"))
s_pred <- summary(fit, pars = c("sesp_pred"))

s_params$summary
```

```
##              mean     se_mean        sd        2.5%        25%       50%
## muSe[1] 1.3298340 0.002381227 0.2398349  0.863589946 1.17210002 1.3240133
## muSe[2] 2.3840455 0.010526918 1.0691371  0.329176308 1.68674375 2.3658143
```

```
## muSe[3] 0.7560181 0.004245671 0.4297309 -0.099887234 0.47466123 0.7570629
## muSe[4] 1.6994473 0.004462234 0.4393424  0.833316285 1.41732243 1.7015604
## muSp[1] 0.9866840 0.002559933 0.2603465  0.470808711 0.82105549 0.9874364
## muSp[2] 3.2944772 0.013008846 1.2631442  0.977210394 2.44642923 3.2376736
## muSp[3] 1.0761897 0.004884832 0.4898935  0.100967922 0.75831417 1.0809132
## muSp[4] 0.9184504 0.005021293 0.4805269 -0.033619815 0.60875422 0.9204484
## sigSe   0.6071556 0.001826238 0.1830956  0.298766479 0.48244927 0.5899561
## sigSp   0.7854083 0.001728316 0.1749424  0.501146656 0.66280912 0.7654502
## tau0_se 0.3039087 0.002557508 0.2435831  0.010993379 0.11621724 0.2523742
## tau1_se 0.2834358 0.002202958 0.2123505  0.009782682 0.11548727 0.2469336
## tau0_sp 0.2709211 0.002417841 0.2335909  0.009538348 0.09763652 0.2144395
## tau1_sp 0.2663329 0.002184337 0.2139158  0.009330613 0.10158469 0.2165887
##               75%     97.5%     n_eff      Rhat
## muSe[1] 1.4798725 1.8169282 10144.331 1.0000552
## muSe[2] 3.0676029 4.5691980 10314.882 1.0002083
## muSe[3] 1.0341225 1.6158105 10244.730 1.0001169
## muSe[4] 1.9875926 2.5624657  9693.968 0.9997182
## muSp[1] 1.1504478 1.5111407 10342.991 0.9998602
## muSp[2] 4.0960213 5.9057117  9428.191 0.9999429
## muSp[3] 1.3951538 2.0358245 10057.825 0.9997108
## muSp[4] 1.2349599 1.8487259  9158.076 0.9999005
## sigSe   0.7151938 1.0211410 10051.733 0.9997495
## sigSp   0.8848916 1.1925212 10245.750 1.0000637
## tau0_se 0.4318700 0.8936228  9071.109 0.9997452
## tau1_se 0.4063978 0.7778997  9291.683 1.0005131
## tau0_sp 0.3842310 0.8619557  9333.761 0.9997852
## tau1_sp 0.3817143 0.7915721  9590.611 0.9999071
```

```
s_p$summary
```

```
##                 mean      se_mean         sd      2.5%       25%       50%
## sepool[1] 0.7880908 0.0003933741 0.03957567 0.7034101 0.7635244 0.7898486
## sepool[2] 0.8809449 0.0010705895 0.10883059 0.5815589 0.8437955 0.9141830
## sepool[3] 0.6737810 0.0008990743 0.09099292 0.4750489 0.6164864 0.6807157
## sepool[4] 0.8370420 0.0006116981 0.06022114 0.6970557 0.8049183 0.8457384
## sppool[1] 0.7254486 0.0005027744 0.05120413 0.6155751 0.6944603 0.7285813
## sppool[2] 0.9365901 0.0007841601 0.07628113 0.7265543 0.9202999 0.9622276
## sppool[3] 0.7354999 0.0009210618 0.09242274 0.5252206 0.6809876 0.7466668
## sppool[4] 0.7055423 0.0010068489 0.09626116 0.4915958 0.6476566 0.7151335
##                 75%     97.5%     n_eff      Rhat
## sepool[1] 0.8145533 0.8601971 10121.506 1.0001379
## sepool[2] 0.9555364 0.9897401 10333.704 0.9998975
## sepool[3] 0.7377143 0.8342165 10242.927 1.0001137
## sepool[4] 0.8794882 0.9284065  9692.229 0.9997222
## sppool[1] 0.7595927 0.8192302 10372.026 0.9998624
## sppool[2] 0.9836336 0.9972836  9462.910 0.9999860
## sppool[3] 0.8014137 0.8845074 10068.856 0.9997013
## sppool[4] 0.7746855 0.8639774  9140.575 0.9999378
```

```
s_pred$summary
```

```
##                      mean     se_mean        sd      2.5%       25%       50%
## sesp_pred[1,1] 0.7579897 0.001562331 0.1557944 0.3624581 0.6784169 0.7922614
```

```
## sesp_pred[1,2] 0.6900982 0.001866995 0.1886558 0.2307504 0.5782194 0.7242946
## sesp_pred[2,1] 0.8594951 0.001553221 0.1565380 0.3998366 0.8139029 0.9163095
## sesp_pred[2,2] 0.9164691 0.001263171 0.1236760 0.5313036 0.9023753 0.9629178
## sesp_pred[3,1] 0.6518639 0.001898809 0.1904416 0.2259390 0.5307443 0.6789362
## sesp_pred[3,2] 0.7082086 0.001935869 0.1916692 0.2421005 0.5955971 0.7473260
## sesp_pred[4,1] 0.8049785 0.001512323 0.1482651 0.3977382 0.7425113 0.8446022
## sesp_pred[4,2] 0.6800041 0.001991188 0.2007960 0.2018135 0.5581417 0.7180181
##                      75%     97.5%     n_eff      Rhat
## sesp_pred[1,1] 0.8724815 0.9626177  9943.914 0.9998033
## sesp_pred[1,2] 0.8375166 0.9562484 10210.661 0.9997814
## sesp_pred[2,1] 0.9645399 0.9941284 10157.180 0.9998479
## sesp_pred[2,2] 0.9873189 0.9985589  9586.201 0.9999387
## sesp_pred[3,1] 0.8005820 0.9395154 10059.148 1.0002414
## sesp_pred[3,2] 0.8573252 0.9662380  9802.852 0.9998557
## sesp_pred[4,1] 0.9105550 0.9754820  9611.444 0.9999922
## sesp_pred[4,2] 0.8376089 0.9593570 10169.172 0.9998957
```

```
s_sediff <- summary(fit, pars = c("se_diff"))
s_spdiff <- summary(fit, pars = c("sp_diff"))

s_sepreddiff <- summary(fit, pars = c("sepred_diff"))
s_sppreddiff <- summary(fit, pars = c("sppred_diff"))

save.image(file=here("data","tmp",
                     paste0("4_3_stanfit_NMA_main_supp",
                            fibrosis_grade,"_",prior_type,".RData")))


# ----------------------------------------------
# Diagnostics
# ----------------------------------------------
pairs(fit, pars = c("muSe[1]", "muSp[1]","sigSe","sigSp"))
```

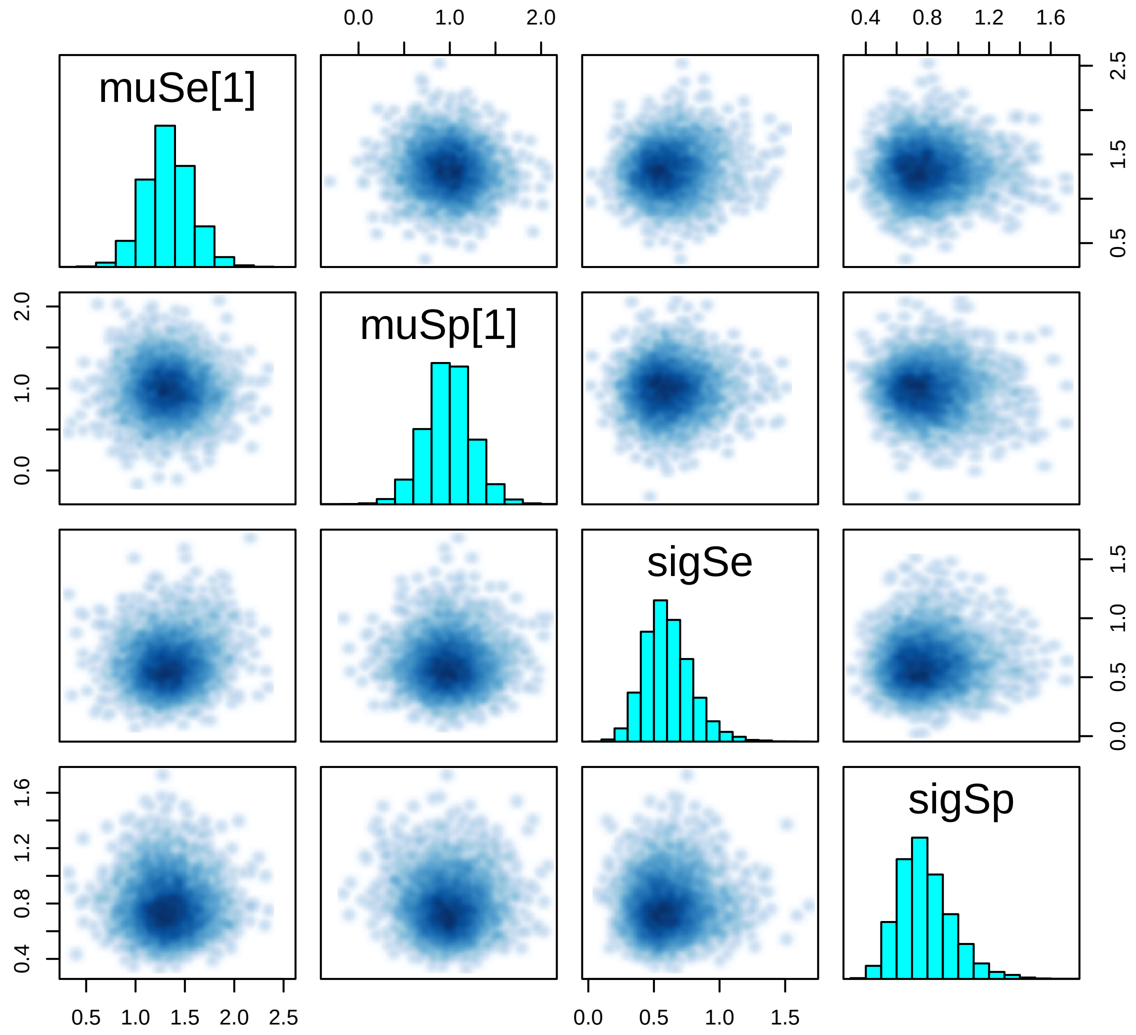


```
pairs(fit, pars = c("tau0_se","tau1_se","tau0_sp","tau1_sp"))
```

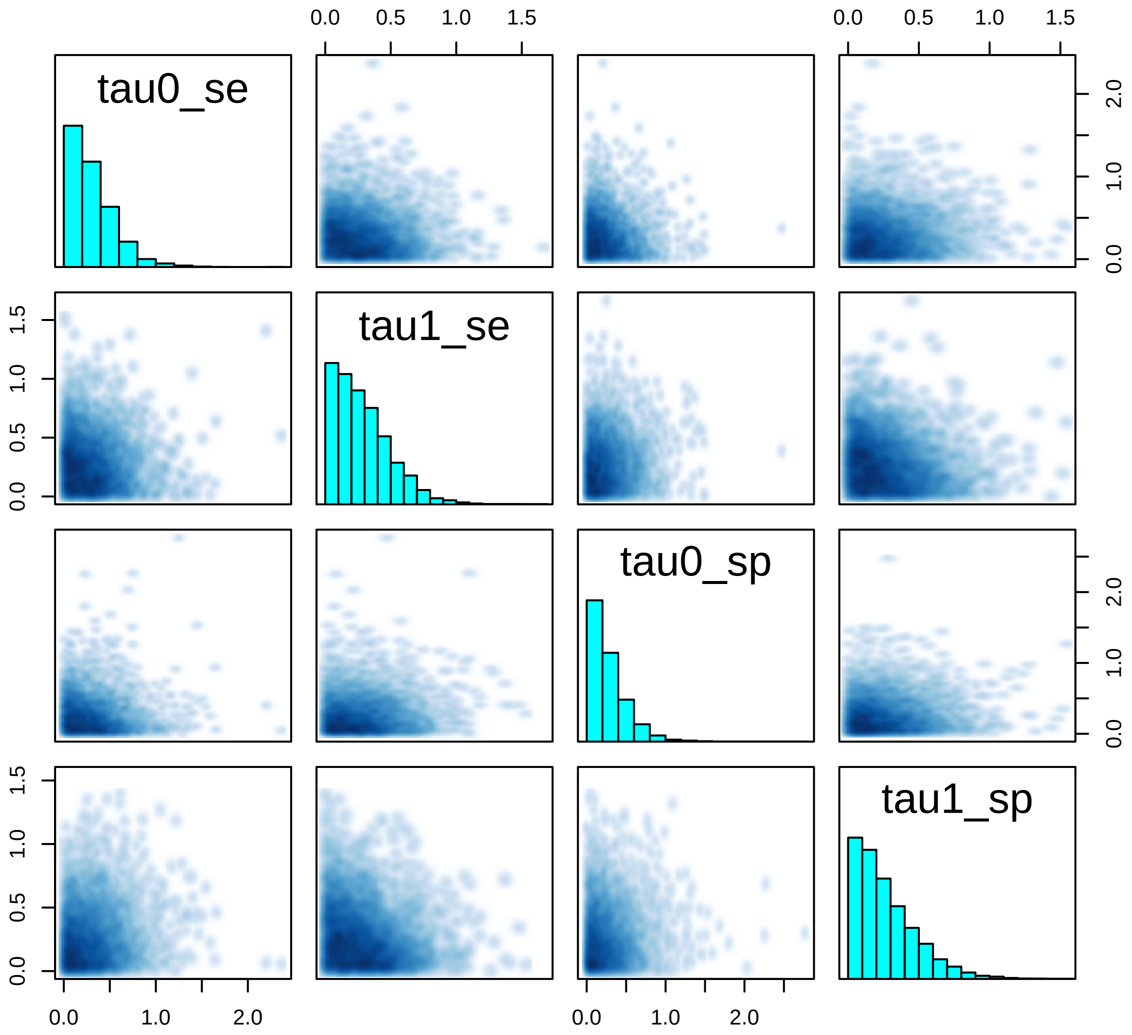


```
pairs(fit, pars = c("gamma0_se[1]","gamma0_sp[1]",
                    "gamma0_se[2]","gamma0_sp[2]",
                    "gamma1_se[1,2]","gamma1_sp[2,3]"))
```

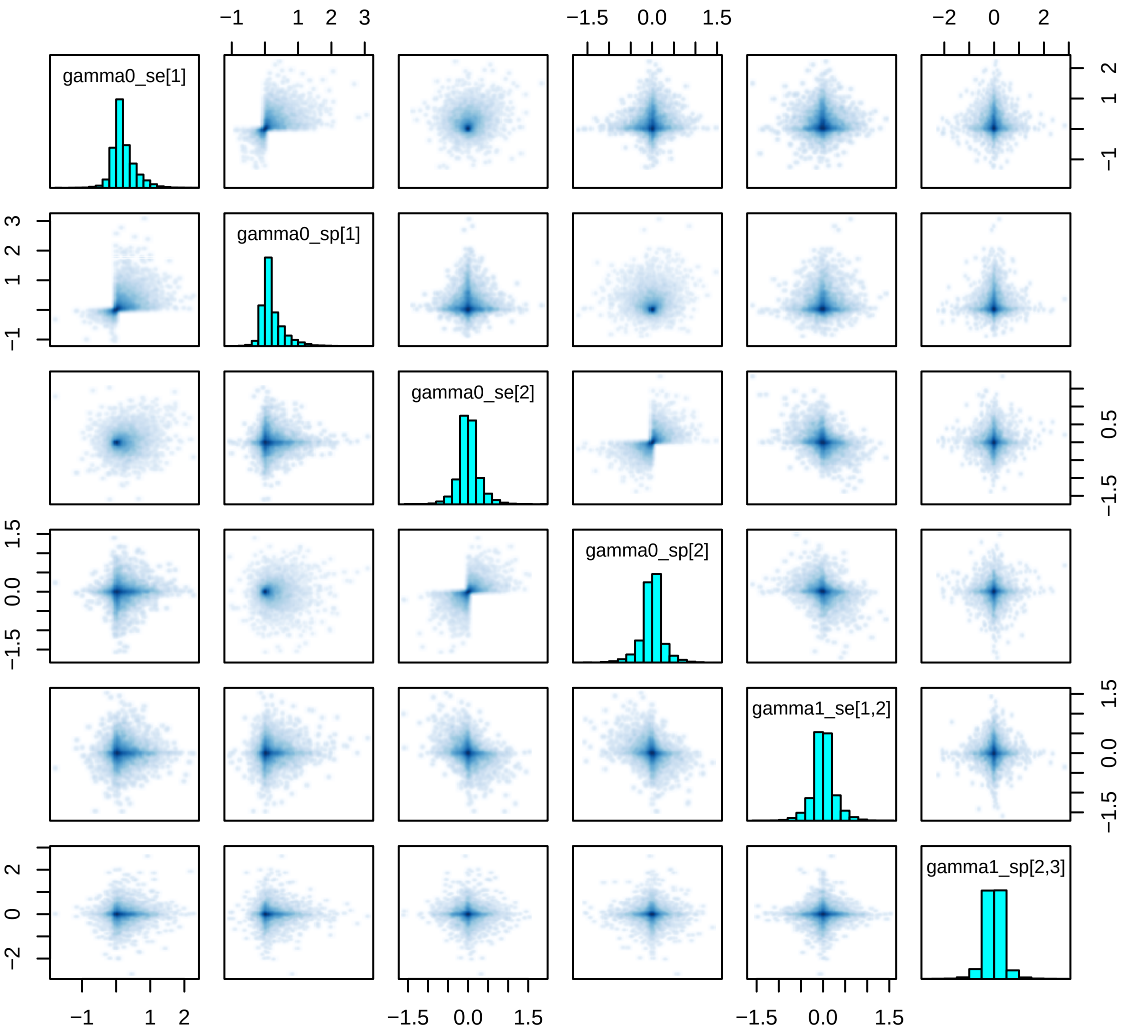


```r
pairs(fit, pars = "sepool")
```

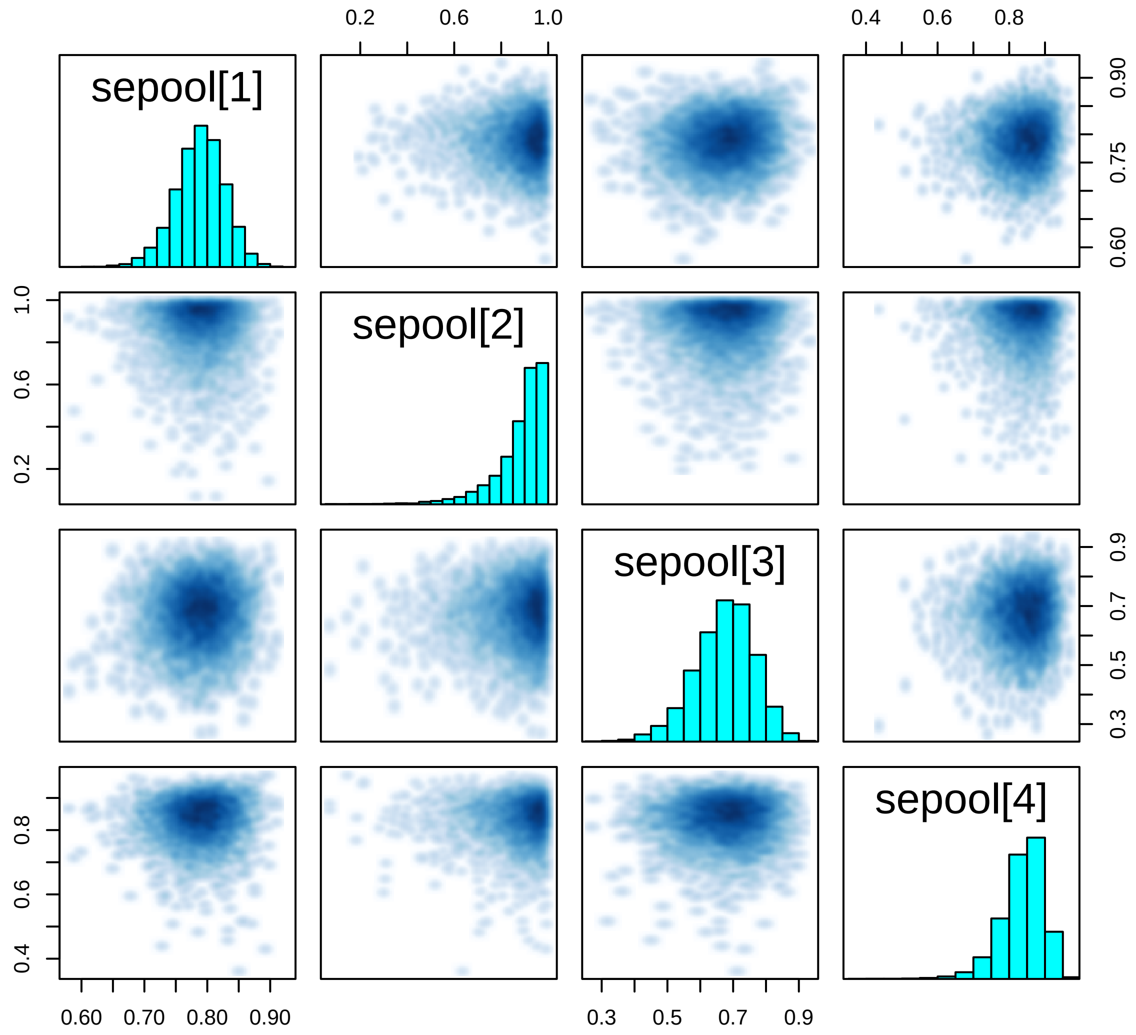


```
pairs(fit, pars = "sppool")
```

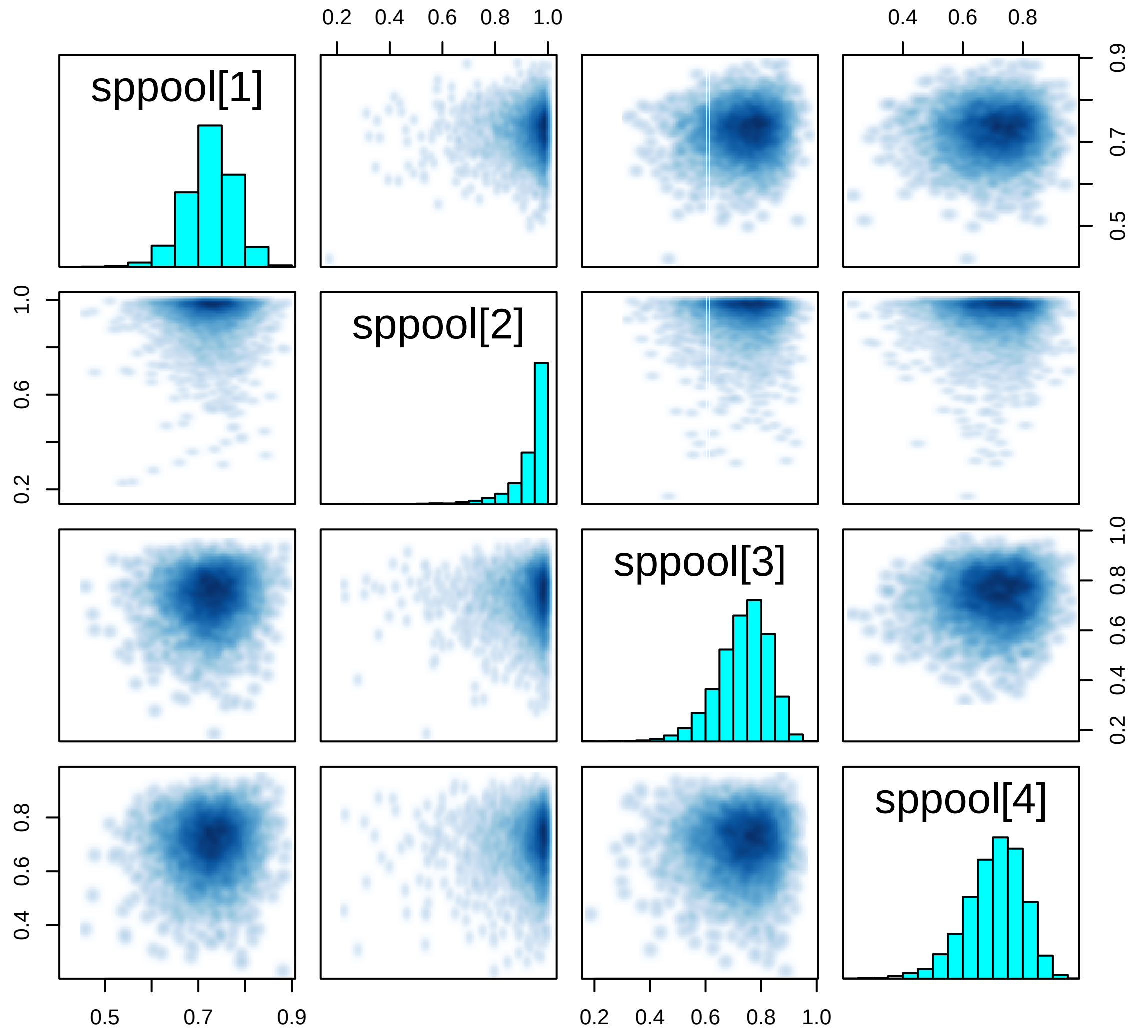


```
# autocorrelations
stan_ac(fit, pars = c("gamma0_se[1]","gamma0_sp[1]",
                      "gamma0_se[2]","gamma0_sp[2]",
                      "gamma1_se[1,2]","gamma1_sp[2,3]"))
```

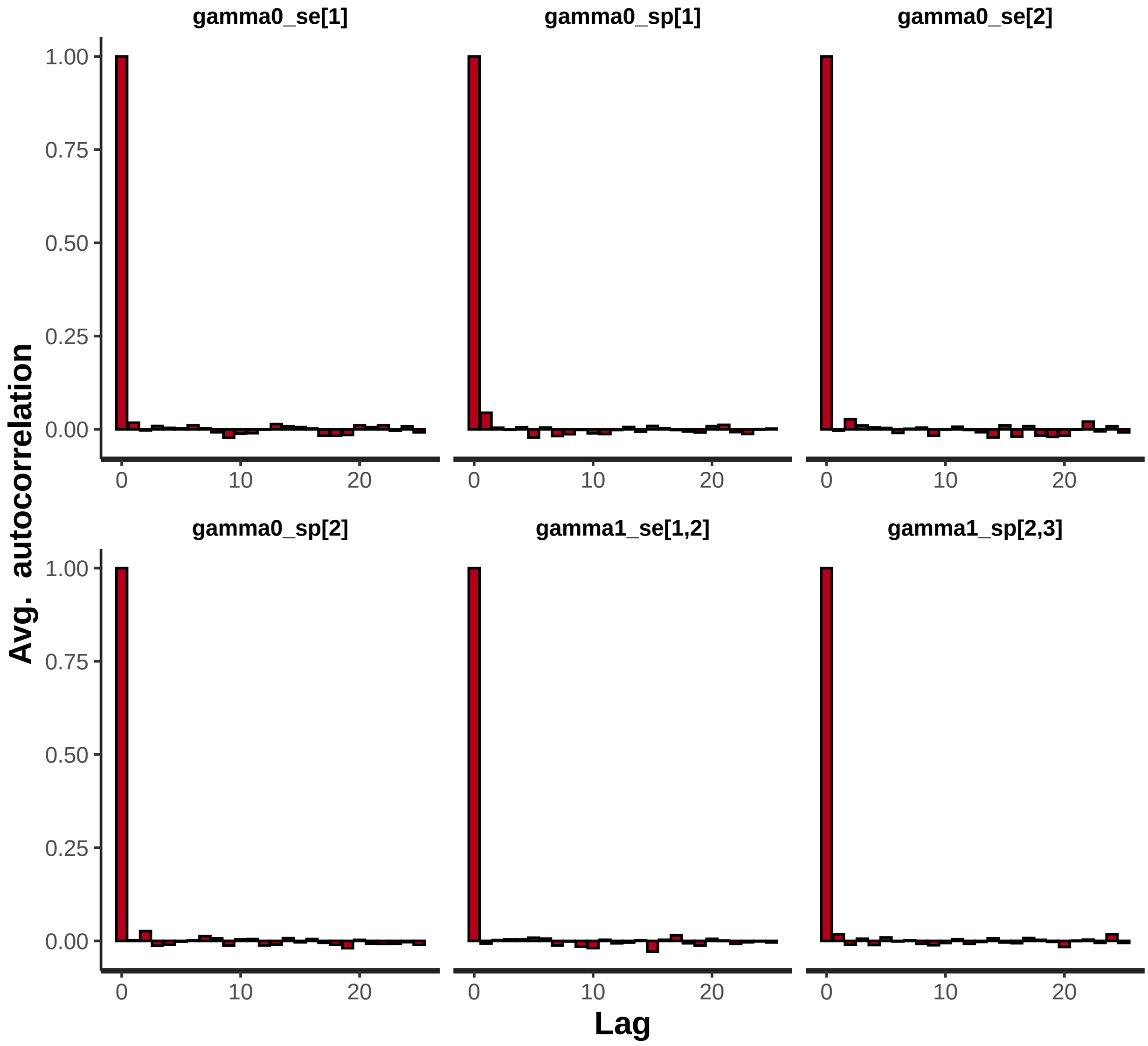


```
stan_ac(fit, pars = parms)
```

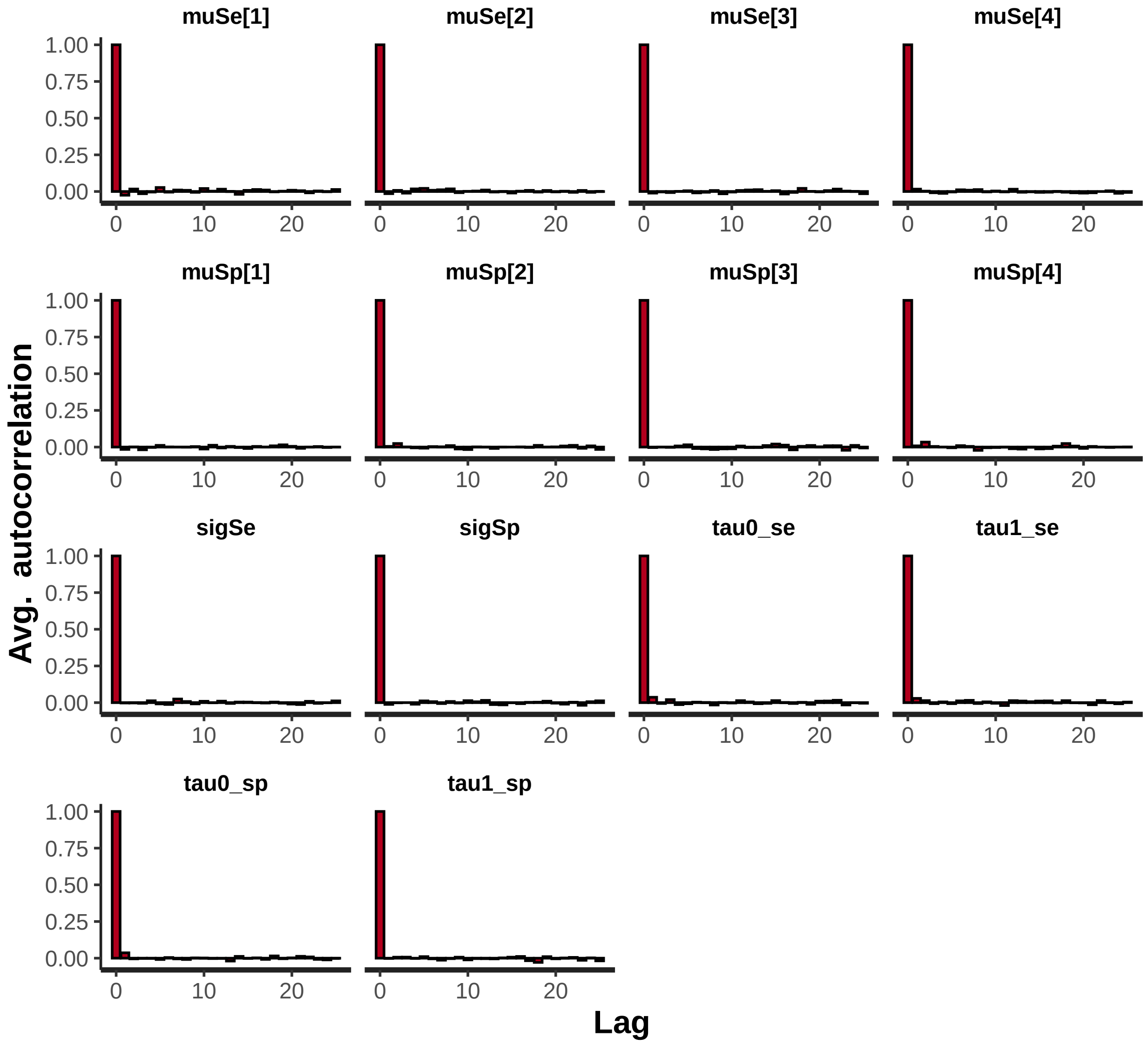


```
# density plots
stan_dens(fit, pars = c("gamma0_se[1]","gamma0_sp[1]",
                        "gamma0_se[2]","gamma0_sp[2]",
                        "gamma1_se[1,2]","gamma1_sp[2,3]"),
          separate_chains = T)
```

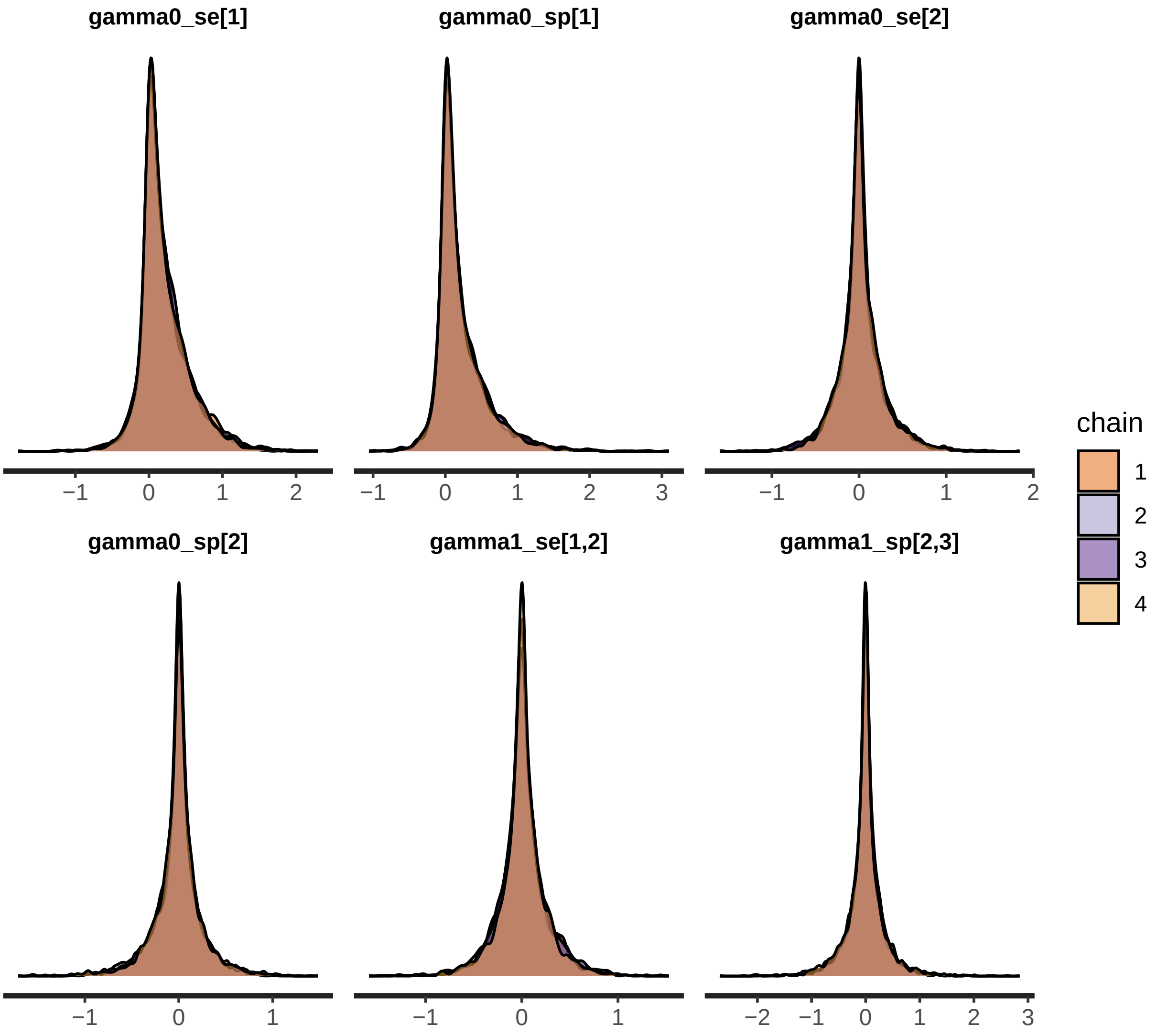


```r
# trace plots
stan_trace(fit, pars = c("gamma0_se[1]","gamma0_sp[1]",
                         "gamma0_se[2]","gamma0_sp[2]",
                         "gamma1_se[1,2]","gamma1_sp[2,3]"))
```

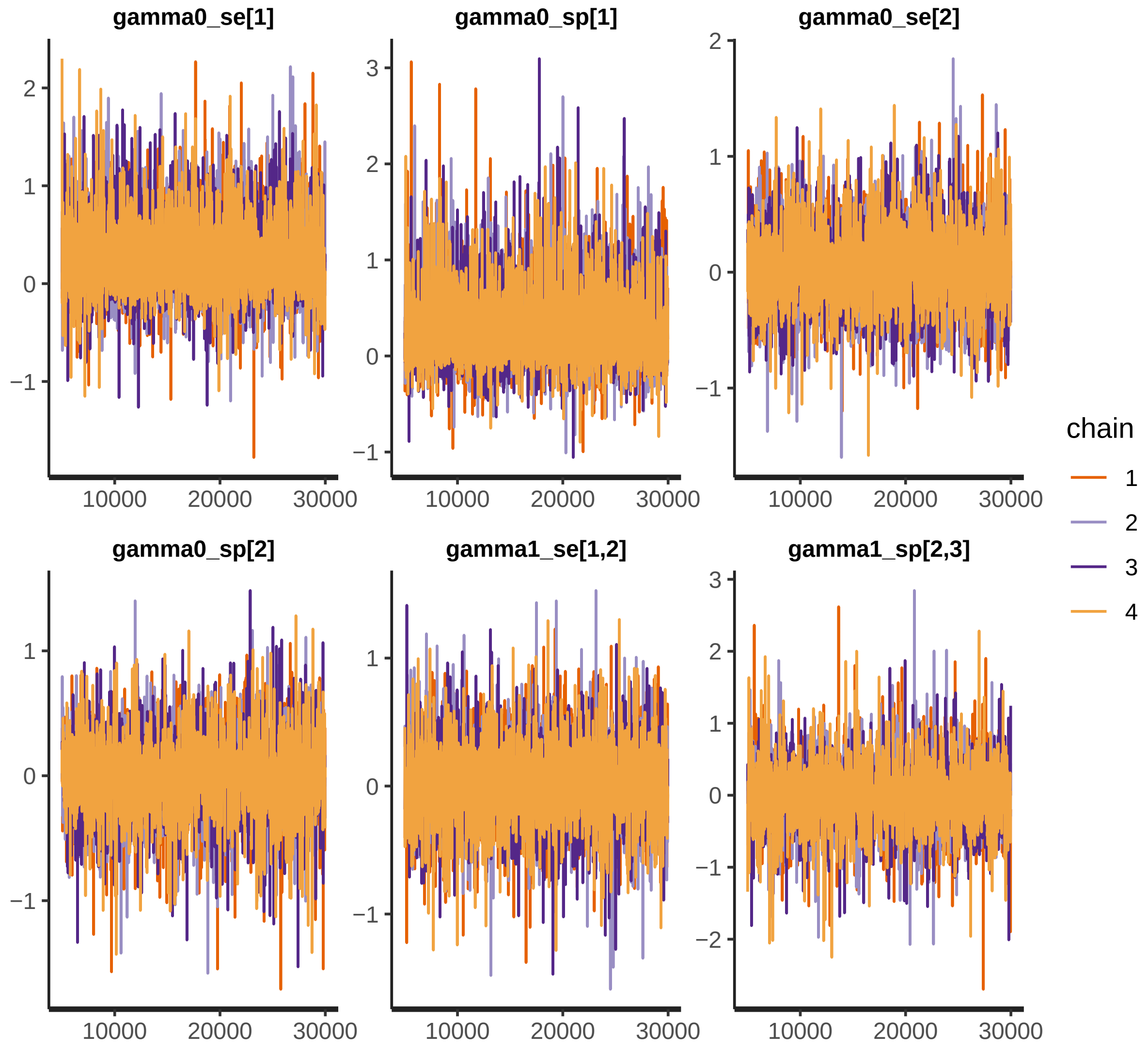


```
# stan diagnosis
stan_diag(fit)
```

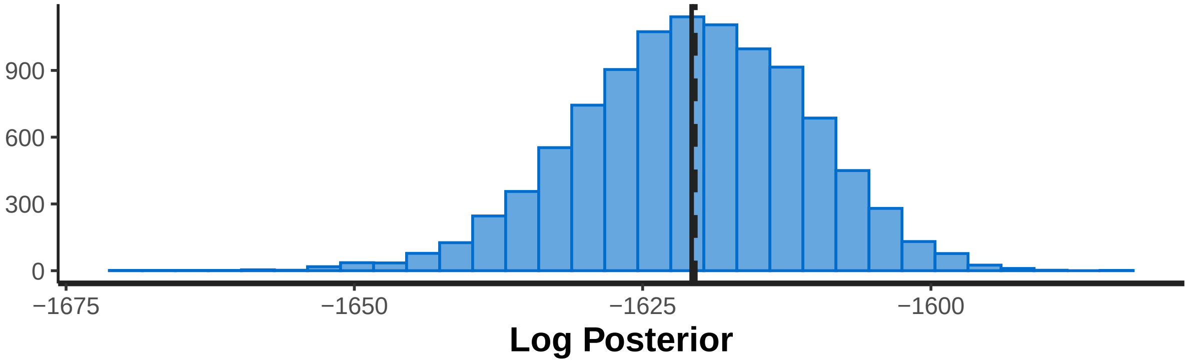


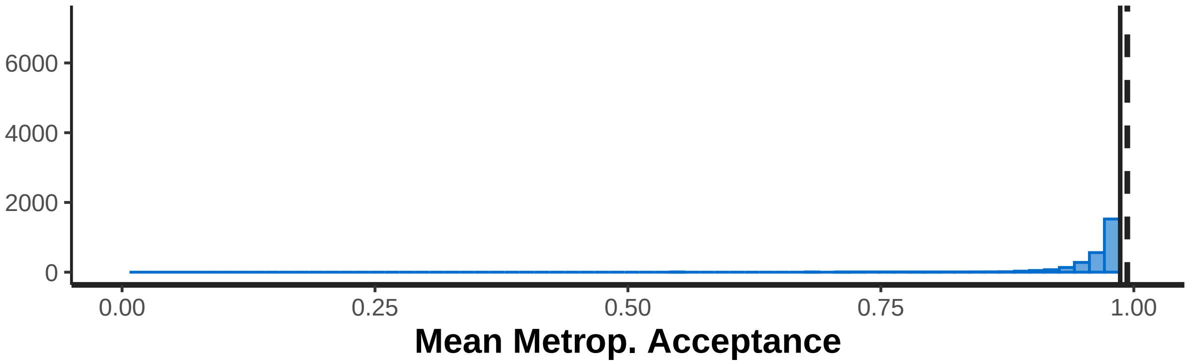


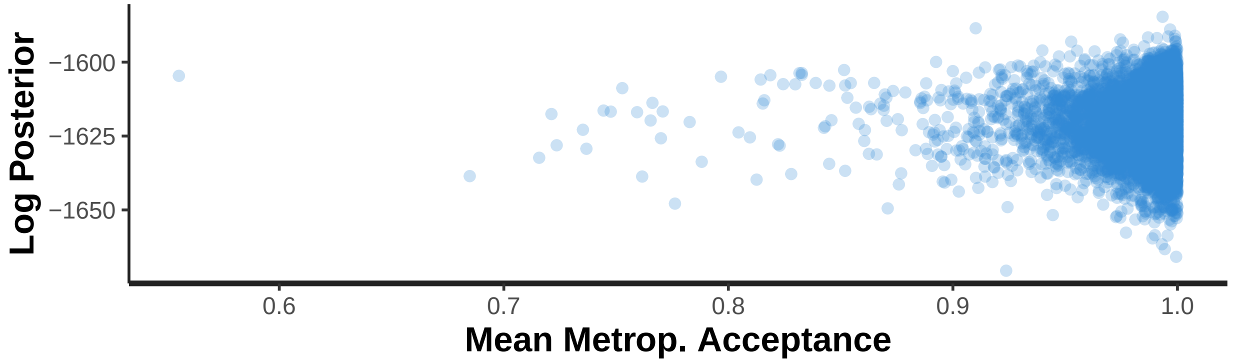


```
# Rhat
sum(stan_rhat(fit)$data > 1.01, na.rm = TRUE)
```

```
## [1] 0
```

```
# DIC
deviance <- -2*rowSums(ms$log_lik)
(dic <- mean(deviance) + var(deviance)/2)
```

```
## [1] 292.4283
```

```
# LooIC
loo2 <-
  loo::loo(ms$log_lik,
           cores = 1,
```

```
           r_eff = loo::relative_eff(
             exp(ms$log_lik),
             chain_id = rep(1:chains_val,
                            each = (iter_val - warmup_val)/thin_val),
             cores = 1
           )
  )

print(loo2)
```

```
##
## Computed from 10000 by 25 log-likelihood matrix
##
##          Estimate  SE
## elpd_loo   -151.3 4.0
## p_loo        37.4 1.9
## looic       302.5 8.1
## ------
## Monte Carlo SE of elpd_loo is NA.
##
## Pareto k diagnostic values:
##                          Count Pct.    Min. n_eff
## (-Inf, 0.5]   (good)      0     0.0%   <NA>
##  (0.5, 0.7]   (ok)        0     0.0%   <NA>
##    (0.7, 1]   (bad)      20    80.0%   32
##    (1, Inf)   (very bad)  5    20.0%   17
## See help('pareto-k-diagnostic') for details.
```

```
plot(loo2)
```

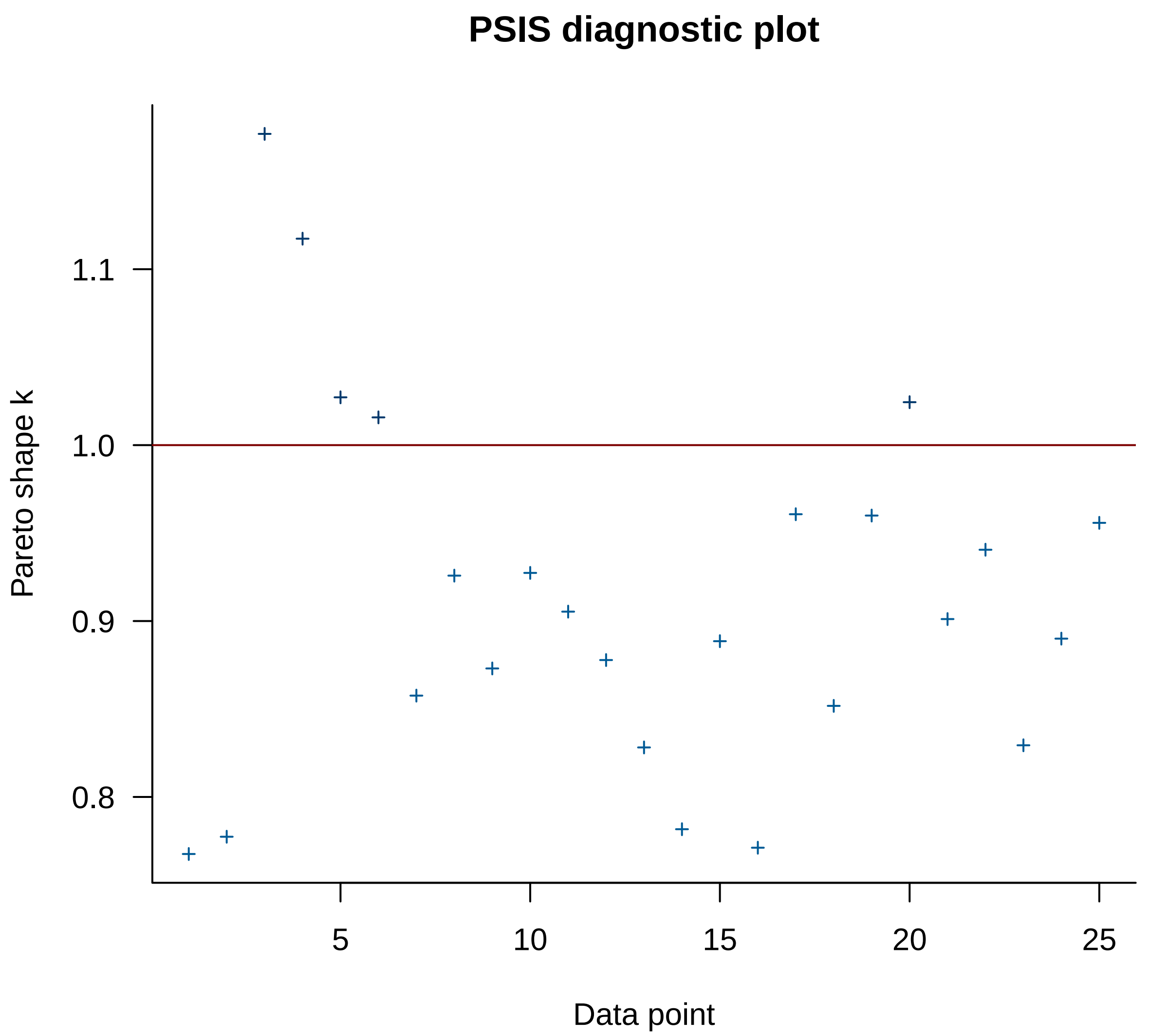


```
loo::pareto_k_influence_values(loo2)
```

```
##  [1] 0.7675808 0.7773874 1.1769281 1.1173616 1.0271775 1.0157811 0.8576152
##  [8] 0.9258189 0.8730562 0.9273665 0.9053538 0.8778324 0.8281478 0.7816760
## [15] 0.8885563 0.7711484 0.9607245 0.8517875 0.9599721 1.0244164 0.9011238
## [22] 0.9405422 0.8293710 0.8900163 0.9558233
```

```
loo::psis_n_eff_values(loo2)
```

```
##  [1]  76.24609 126.88569  23.93887  18.42223  19.60022  29.41779 157.68391
##  [8]  40.54522 126.48864  33.78246 131.34865  89.74964  66.41947 125.15102
## [15]  61.51991 157.33665  40.10287 109.57505  64.68558  16.68975  50.58170
## [22]  31.83699 150.48084 141.65694  40.83336
```

```r
# WAIC
loo::waic(ms$log_lik)
```

```
##
## Computed from 10000 by 25 log-likelihood matrix
##
##           Estimate  SE
## elpd_waic   -138.0 3.8
## p_waic        24.2 1.3
## waic         276.0 7.7
##
## 25 (100.0%) p_waic estimates greater than 0.4. We recommend trying loo instead.
```
