# Supplementary material for: Comparison of the diagnostic accuracy of shear wave elastography with transient elastography in adult nonalcoholic fatty liver disease: a systematic review and network meta-analysis of diagnostic test accuracy
Source: Abdom Radiol (NY). 2024 Sep 6;50(2):734–46. doi: 10.1007/s00261-024-04546-8 (PMC11794403; doi:10.1007/s00261-024-04546-8)
Supplement: Supplementary file 4 — Supplementary file4 (PDF 6326 KB) [file 261_2024_4546_MOESM4_ESM.pdf]

Fitting and convergence diagnostics of NMA analysis with the incosistency model

```r
# ########################################################################
# - This is a R script to fit a NMA inconsistency model.
# - Created by Kengo Nagashima
# ########################################################################

# ---------------------------------------------------
# Preparation
# ---------------------------------------------------

library(tidyverse)
library(rstan)
source("create_datasets_supp.R")

fibrosis_grade <- "F2"
prior_type <- 1

# Read data
d <- readxl::read_excel("NMA_data_inconsistency.xlsx", sheet = 1)

# Include studies whose cutoffs are not defined to get 90 % of sens or spe
d2<-
  d %>%
  filter(Flag_Threshold_sesp_90 == 0)

# Create a dataset for network meta-analysis
df <- create_dataset_NMA(d2, fibrosis_grade)

# Further add flags for inconsistenct parameters
d_inconsistency <-
  df %>%
  mutate(
    fTE = case_when(Test == "TE" ~ 1, TRUE ~ 0),
    fpSWE = case_when(Test == "pSWE" ~ 1, TRUE ~ 0),
    ftSWE = case_when(Test == "2D-SWE" ~ 1, TRUE ~ 0),
    fMRE = case_when(Test == "MRE" ~ 1, TRUE ~ 0)
  ) %>%
  group_by(`Rayyan ID`) %>%
  summarize(TE = max(fTE), pSWE = max(fpSWE),
            tSWE = max(ftSWE), MRE = max(fMRE)) %>%
  mutate(
    id = `Rayyan ID`,
    wtype = case_when(
      TE == 1 & pSWE == 1 & tSWE == 1 & MRE == 0 ~ 1,
      TE == 1 & pSWE == 1 & tSWE == 0 & MRE == 0 ~ 2,
```

```r
      TE == 0 & pSWE == 1 & tSWE == 1 & MRE == 0 ~ 3,
      TE == 0 & pSWE == 1 & tSWE == 0 & MRE == 1 ~ 4
    )
  )

df <-
  df %>% mutate(id = `Rayyan ID`)

df <-
  left_join(df, d_inconsistency, by = "id") %>%
  mutate(
    wtype = case_when(
      Test == "pSWE" ~ 0,
      TRUE ~ wtype
    ))

# Create a dataset for stan
data_stan <- list(N = nrow(df),
                  J = 2,
                  S = length(unique(df$id)),
                  T = length(unique(df$Test)),
                  Test = df$Test_Num,
                  Study = df$Study_Num,
                  Threshold = df$Threshold_Num,
                  TP = df$TP*10^3,
                  FP = df$FP*10^3,
                  FN = df$FN*10^3,
                  TN = df$TN*10^3,
                  prior_type = prior_type,
                  W = df$wtype
)

# ---------------------------------------------------
# Fitting
# ---------------------------------------------------

# Compile a model
model <- stan_model("nma_fit_main_inconsistency_supp.stan")

# Set some options
options(mc.cores = parallelly::availableCores() - 1)
rstan_options("auto_write" = TRUE)
rstan_options(javascript = FALSE)

# Set values for some arguments in sampling()
iter_val <- 30000
warmup_val <- 5000
chains_val <- 4
thin_val <- 10

# MCMC
fit <- sampling(
  model,
```

```r
  data = data_stan,
  seed = 9999,
  iter = iter_val,
  warmup = warmup_val,
  chains = chains_val,
  thin = thin_val,
  control = list(adapt_delta = 0.99,
                 max_treedepth = 12)
)

# Store large outputs
parms <- c("muSe", "muSp", "sigSe", "sigSp",
           "tau0_se", "tau1_se", "tau0_sp", "tau1_sp",
           "omegaSe[1]", "omegaSe[2]", "omegaSp[1]", "omegaSp[2]",
           "sepool[1]", "sepool[2]", "sepool[3]", "sepool[4]",
           "sppool[1]", "sppool[2]", "sppool[3]", "sppool[4]")

ms <- rstan::extract(fit)
s_params <- summary(fit, pars = parms)
s_params$summary[,c("mean","2.5%","50%","97.5%")]
```

```
##                   mean        2.5%        50%     97.5%
## muSe[1]      0.8800937 -0.06801982  0.8824390 1.8213443
## muSe[2]      2.3263008  0.21813043  2.3201635 4.4806481
## muSe[3]      0.7412012 -0.17360403  0.7456580 1.6201245
## muSe[4]      1.7890893  0.76083535  1.7922163 2.7758874
## muSp[1]      1.1349729  0.03445385  1.1407083 2.2239285
## muSp[2]      3.2452297  0.79652277  3.2147633 5.8620085
## muSp[3]      1.0678985  0.06851379  1.0685274 2.0704305
## muSp[4]      0.6766777 -0.39203206  0.6766845 1.7483166
## sigSe        0.6083610  0.29118729  0.5876011 1.0438214
## sigSp        0.7881980  0.49384462  0.7654261 1.2118787
## tau0_se      0.3389147  0.01271708  0.2826977 0.9833567
## tau1_se      0.3167272  0.01220411  0.2734759 0.8735991
## tau0_sp      0.3047391  0.01014488  0.2396210 0.9681960
## tau1_sp      0.2862146  0.01076167  0.2325192 0.8697885
## omegaSe[1]   0.5983843 -0.44755149  0.5914043 1.6848182
## omegaSe[2]  -0.5714446 -2.35597845 -0.5807277 1.2599282
## omegaSp[1]  -0.1809644 -1.39696015 -0.1854135 1.0449797
## omegaSp[2]   0.9077070 -1.08631275  0.9151619 2.8835996
## sepool[1]    0.6979511  0.48300160  0.7073274 0.8607274
## sepool[2]    0.8744351  0.55431740  0.9105333 0.9888008
## sepool[3]    0.6702315  0.45670767  0.6782319 0.8348123
## sepool[4]    0.8459567  0.68153507  0.8571988 0.9413588
## sppool[1]    0.7436133  0.50861261  0.7578097 0.9023778
## sppool[2]    0.9324992  0.68923017  0.9613861 0.9971626
## sppool[3]    0.7333304  0.51712175  0.7443168 0.8879958
## sppool[4]    0.6534465  0.40322822  0.6629983 0.8517403
```

```r
parms <- c(
  "d_Se_TE_pSWE_direct", "d_Sp_TE_pSWE_direct",
  "d_Se_2dSWE_pSWE_direct", "d_Sp_2dSWE_pSWE_direct",
  "d_Se_TE_pSWE_indirect", "d_Sp_TE_pSWE_indirect",
  "d_Se_MRE_pSWE_indirect", "d_Sp_MRE_pSWE_indirect",
```

```r
  "d_Se_2dSWE_pSWE_indirect", "d_Sp_2dSWE_pSWE_indirect"
)

s_params <- summary(fit, pars = parms)
s_params$summary[,c("mean","2.5%","50%","97.5%")]
```

```
##                                 mean       2.5%         50%    97.5%
## d_Se_TE_pSWE_direct       0.13889250 -1.1240385  0.13708026 1.404300
## d_Sp_TE_pSWE_direct       0.06707433 -1.3953939  0.07098286 1.508217
## d_Se_2dSWE_pSWE_direct    1.04788809 -0.2573843  1.04849991 2.322640
## d_Sp_2dSWE_pSWE_direct   -0.39122087 -1.8279644 -0.39593007 1.068371
## d_Se_TE_pSWE_indirect     0.73727677 -0.2435103  0.72672533 1.779397
## d_Sp_TE_pSWE_indirect    -0.11389008 -1.2316205 -0.11573023 1.013498
## d_Se_MRE_pSWE_indirect    1.58509953 -0.6947143  1.58460555 3.859636
## d_Sp_MRE_pSWE_indirect    2.17733116 -0.4591201  2.14955525 4.970919
## d_Se_2dSWE_pSWE_indirect  0.47644349 -1.3055997  0.46085224 2.334416
## d_Sp_2dSWE_pSWE_indirect  0.51648615 -1.5588278  0.51412449 2.566401
```

```r
save.image(file=paste0("stanfit_NMA_main_supp_inconsistency",
                       fibrosis_grade,"_",prior_type,".RData"))

# ----------------------------------------------------
# Check MCMC samples
# ----------------------------------------------------
parms <- c(parms, c("gamma0_se[1]", "gamma0_sp[1]",
                    "gamma0_se[2]", "gamma0_sp[2]",
                    "gamma1_se[1,2]", "gamma1_sp[2,3]"))

pairs(fit, pars = c("muSe[1]", "muSp[1]", "sigSe", "sigSp"))
```

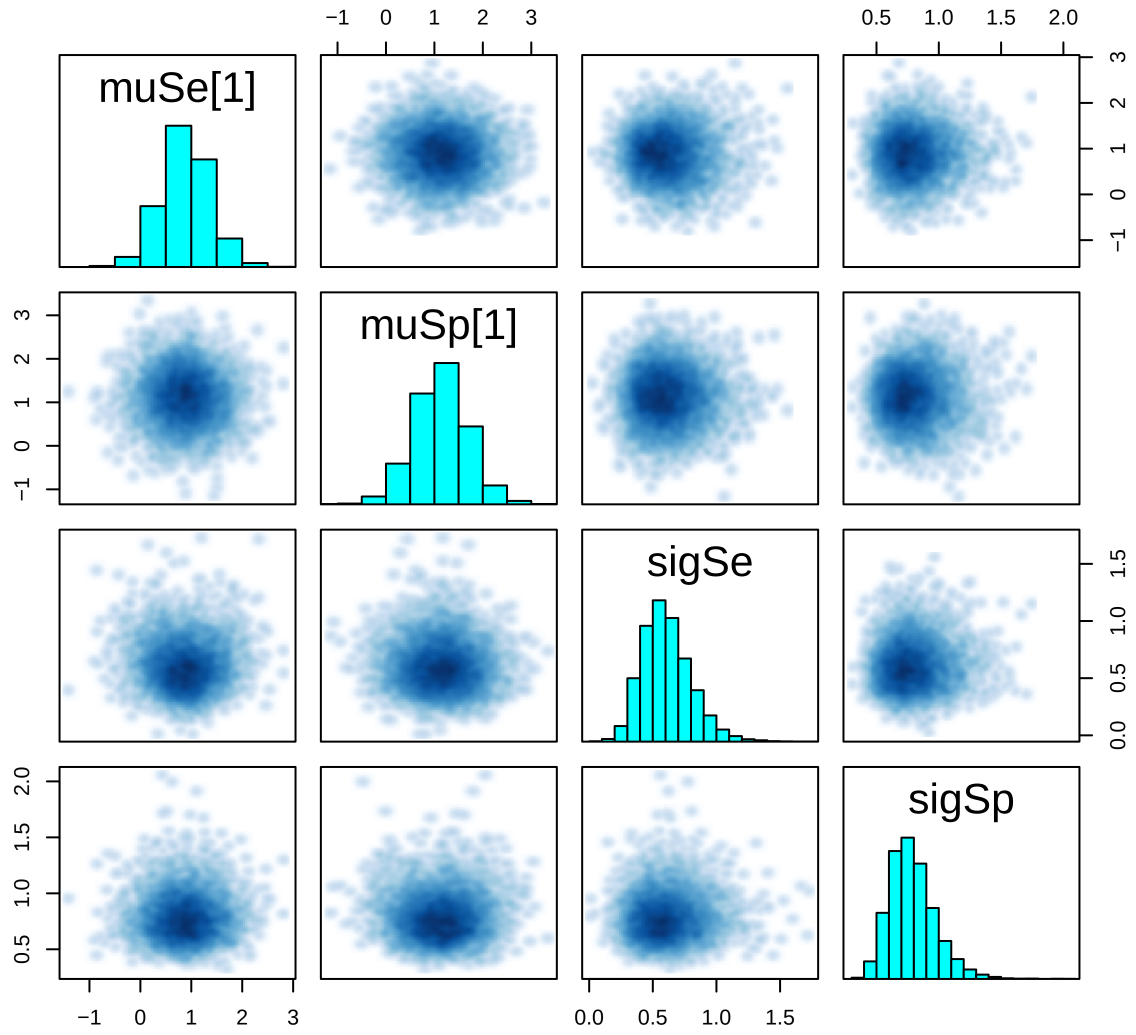


```r
pairs(fit, pars = c("tau0_se", "tau1_se", "tau0_sp", "tau1_sp"))
```

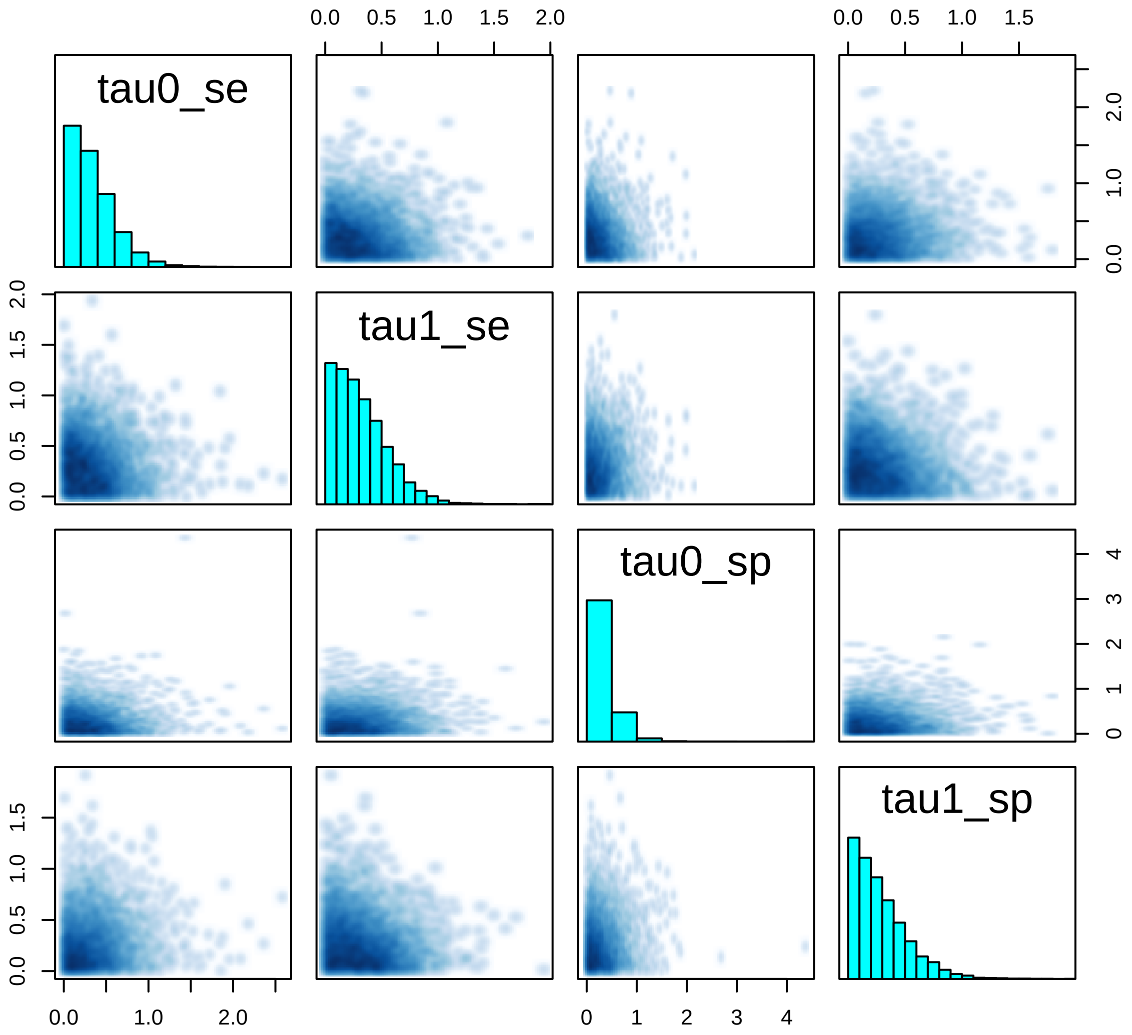


```r
pairs(fit, pars = c("gamma0_se[1]", "gamma0_sp[1]",
                    "gamma0_se[2]", "gamma0_sp[2]",
                    "gamma1_se[1,2]", "gamma1_sp[2,3]"))
```

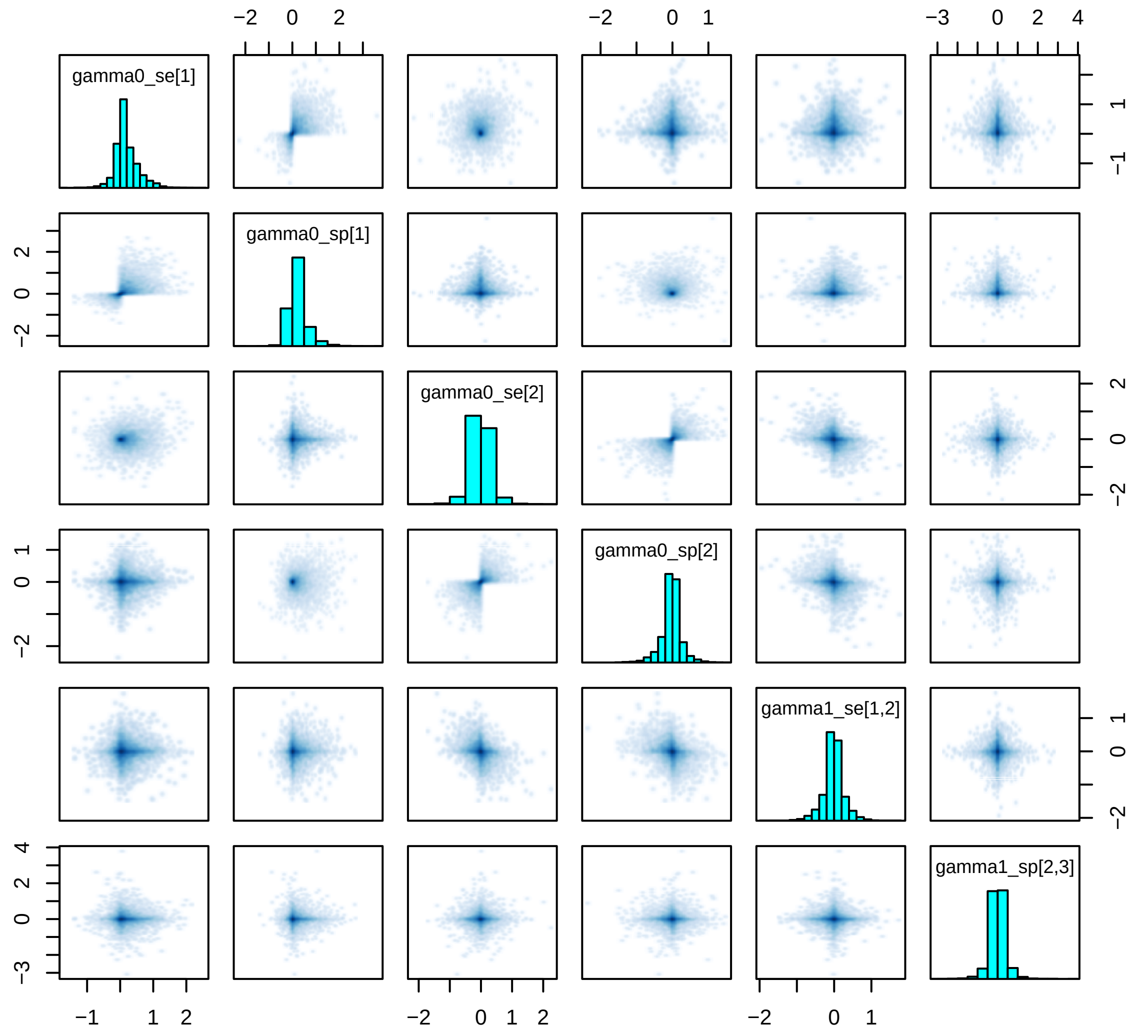


```r
pairs(fit, pars = c("omegaSe[1]", "omegaSe[2]",
                    "omegaSp[1]", "omegaSp[2]"))
```

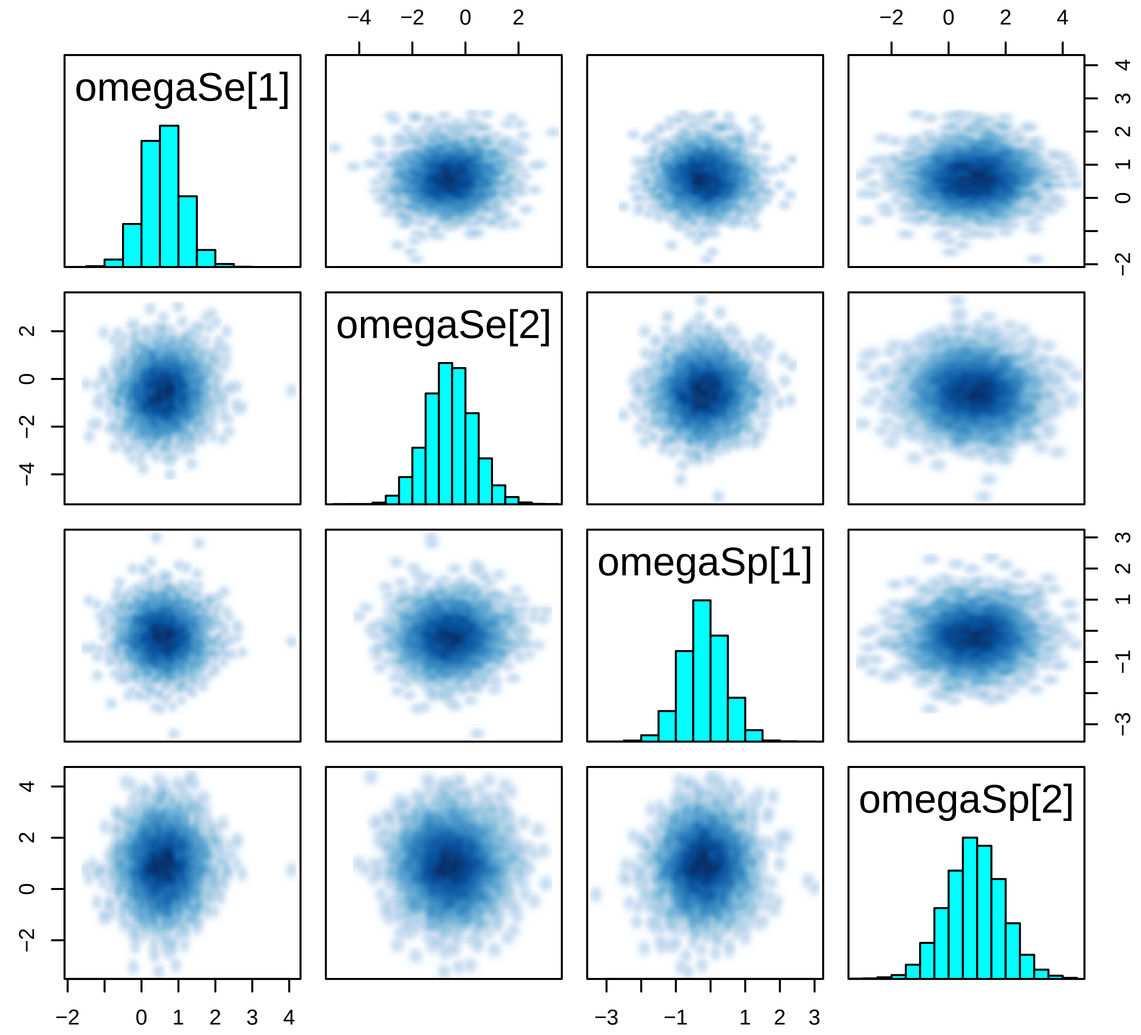


```r
# Autocorrelations
stan_ac(fit, pars = parms)
```

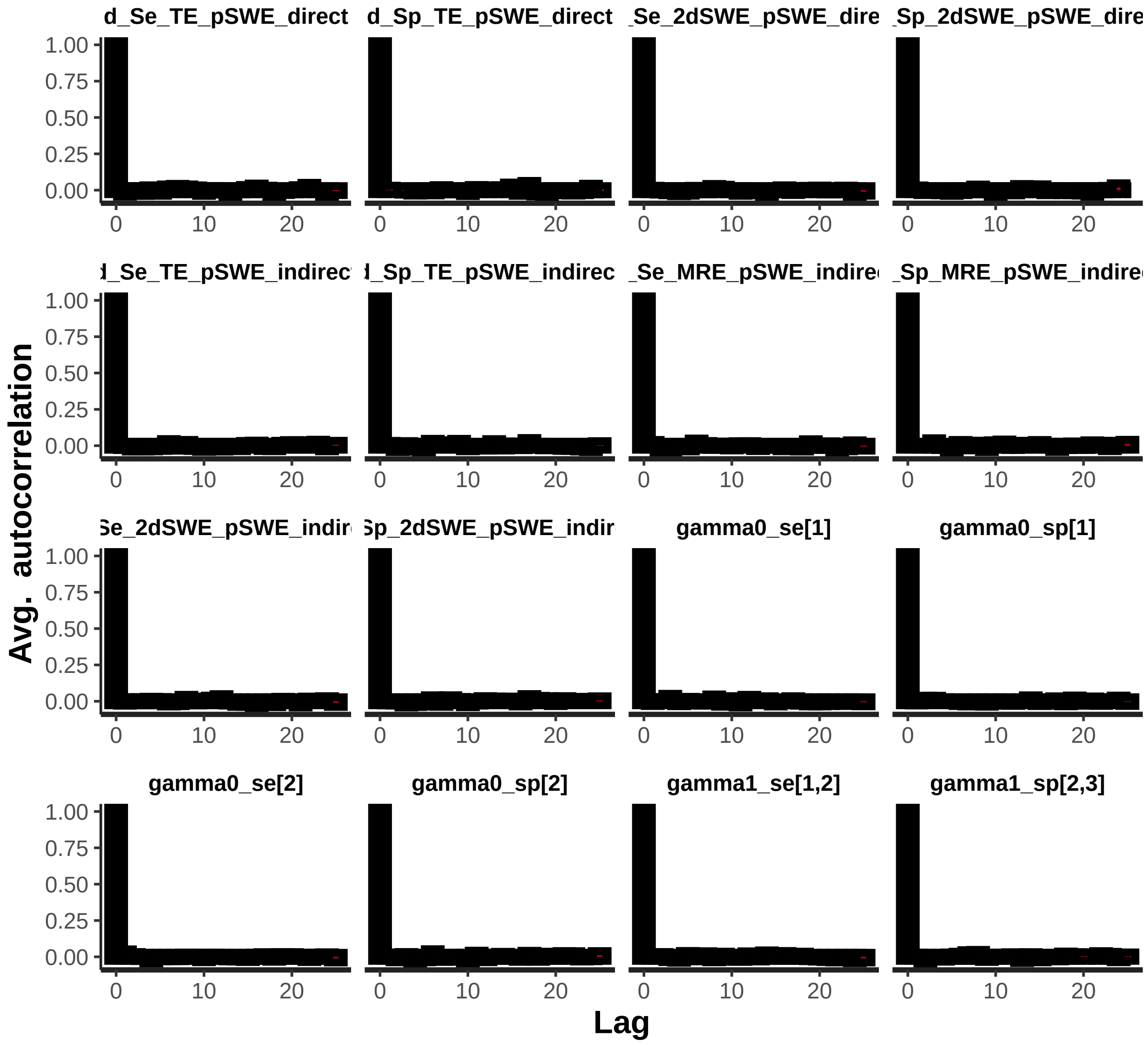


```r
# Density plots
stan_dens(fit, pars = parms, separate_chains = T)
```

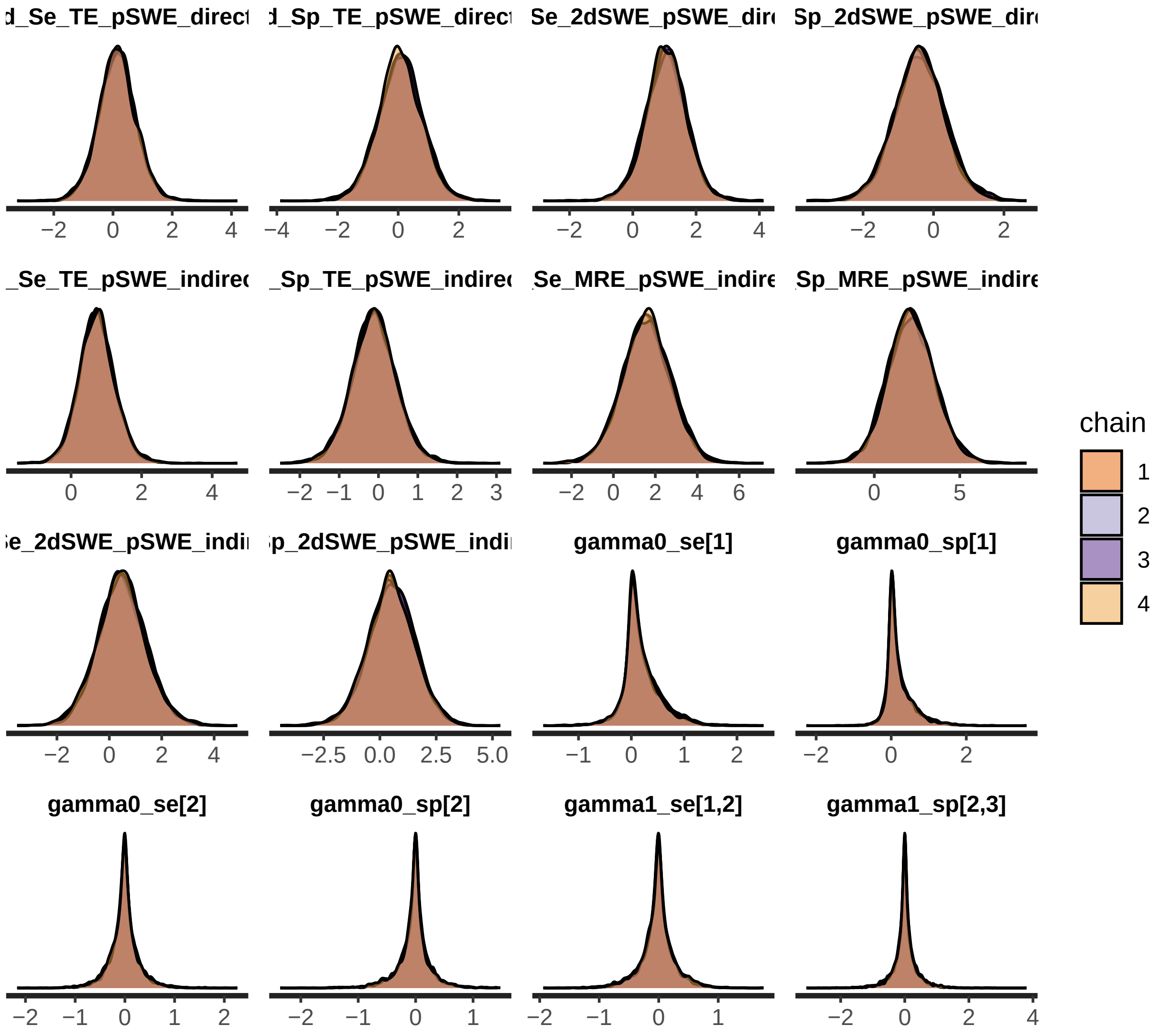


```r
# Trace plots
stan_trace(fit, pars = parms)
```

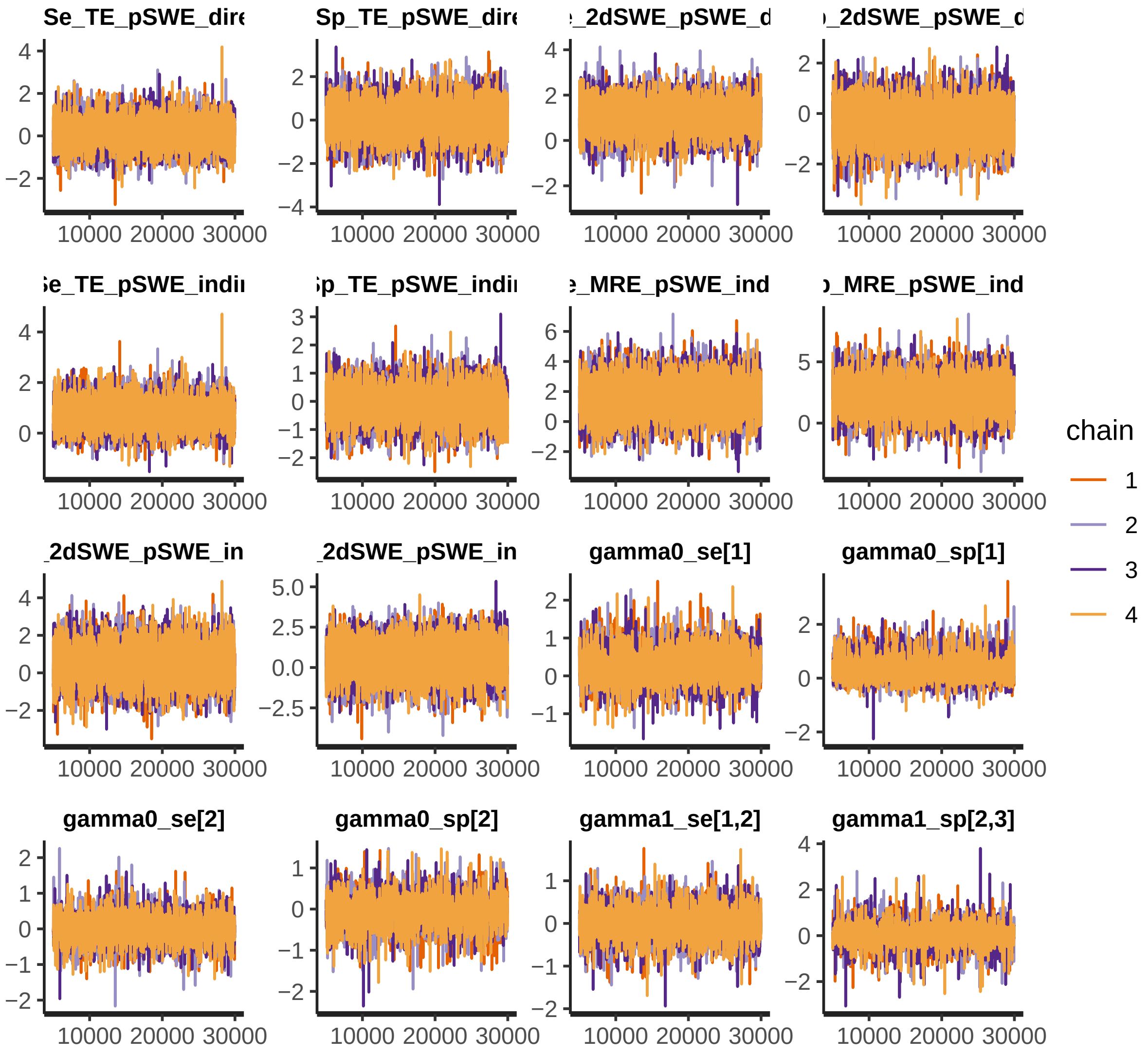


```
# Stan diagnosis
stan_diag(fit)
```

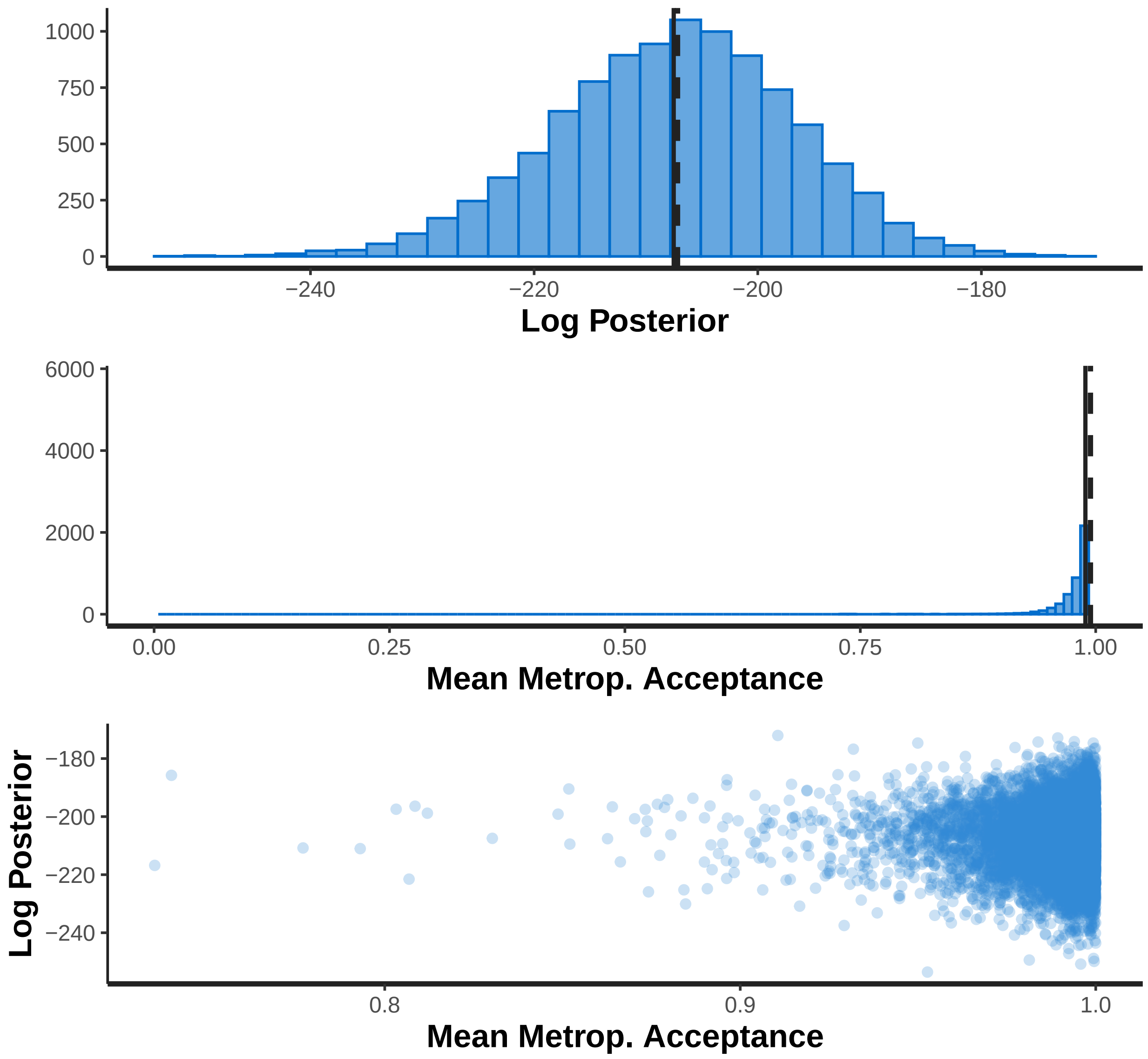


```
# Rhat
sum(stan_rhat(fit)$data > 1.01, na.rm = TRUE)
```

```
## [1] 0
```
